# Exploring the activity of the putative Δ6-desaturase and its role in bloodstream form life-cycle transitions in *Trypanosoma brucei*

**DOI:** 10.1371/journal.ppat.1012691

**Published:** 2025-02-18

**Authors:** Michela Cerone, Terry K. Smith

**Affiliations:** Schools of Chemistry and Biology, Biomedical Sciences Research Complex, North Haugh, University of St Andrews, St Andrews, Scotland; San Diego State University, UNITED STATES OF AMERICA

## Abstract

Trypanosomatids have been shown to possess an exclusive and finely regulated biosynthetic pathway for *de novo* synthesis of fatty acids (FAs) and particularly of polyunsaturated fatty acids (PUFAs). The key enzymes for the process of unsaturation are known as desaturases. In this work, we explored the biocatalytic activity of the putative Δ6-desaturase (Tb11.v5.0580) in the native organism *T. brucei*, whose expression level varies dramatically between life cycle stages. Utilising FA analysis *via* GC-MS, we were able to elucidate i) *via* genetic manipulation of the level of expression of Δ6-desaturases in both procyclic (PCF) and bloodstream (BSF) forms of *T. brucei* and ii) *via* supplementation of the media with various levels of FA sources, that docosahexaenoic acid (22:6) and/or docosapentaenoic acid (22:5) are the products, while arachidonic acid (20:4) and/or docosatetraenoic acid (22:4) are the substrates of this Δ6-desaturase. Surprisingly, we were able to observe, *via* lipidomic analysis with ESI-MS/MS, an increase in inositol-phosphoryl ceramide (IPC) in response to the overexpression of Δ6-desaturase in low-fat media in BSF. The formation of IPC is normally only observed in the stumpy and procyclic forms of *T. brucei*. Therefore, the expression levels of Δ6-desaturases, which increases between BSF, stumpy and PCF, might be involved in the cascade(s) of metabolic events that contributes to these remodelling of the lipid pools and ultimately morphological changes, which are key to the transition between these life-cycle stages. We were in fact able to show that the overexpression of Δ6-desaturase is indeed linked to the expression of protein associated with differentiation (PAD1) in stumpy, and of the upregulation of some proteins and metabolites which are normally upregulated in stumpy and PCF.

## Author summary

*Trypanosoma brucei* is a unicellular parasite that causes human and animal African trypanosomiasis, which is transmitted by the bite of the Tsetse fly. These parasites have the special ability to make their own pool of fatty molecules by assembling and modifying the fatty acid building blocks that they take up from their mammalian and insect hosts. In this study, we investigated the unknown activity of a desaturase enzyme. By modulating its activity, we showed that it can make different levels of long chain polyunsaturated fatty acids (LC-PUFAs), often known as omega-6 and omega-3. If we increase or reduce the fat sources available from the environment, the cells respond by making i) more or less LC-PUFAs and ii) by forming different type of lipids and sphingolipids for their cellular membranes. We highlighted that by tuning the level of activity of the desaturase enzyme and varying the type and amounts of fat sources available to the cells, *T. brucei* can alter their morphology. This is key for the parasites to adapt to the various environments and the nutrients they are exposed to when changing between hosts. Hence, these important shifts between different life-cycle stages get the parasites ready and adapted for survival as they go from a mammalian host to an insect vector and back again.

## Introduction

*Trypanosoma brucei* is an extracellular kinetoplastid parasite and the causative agent of Human and Animal African Trypanosomiasis (HAT, AAT) [1–3]. *T. brucei* is transmitted by the Tsetse fly to humans and livestock animals when it takes a blood meal. In the insect vector, the parasites proliferate as procyclic form (PCF) and differentiate into epimastigotes, and finally into the infective metacyclic trypomastigotes within the salivary glands of the Tsetse fly [1,2]. Once in the bloodstream, the parasites transform into slender bloodstream form (BSF) and circulate in the bloodstream, but also invade the extracellular space of many organs including skin, adipose tissue, lymph and central nervous system [2,4]. Some of the BSF *T. brucei* responding to a quorum sensing mechanism routinely differentiate into the pre-adaptive and non-replicative stumpy form, which is pre-adapted to survive when taken up by the fly with a blood meal [1,5–7]. Throughout this complex life cycle, *T. brucei* showcase unique biochemical mechanisms of adaptation that have been acquired during evolution as early divergent eukaryote [8]. These efficient adaptation mechanisms are key to the parasites’ survival during the life-cycle progression from the mammalian host to the insect vector and back [9]. During this process, *T. brucei* find not only different levels of different nutrients to scavenge and use as metabolic building blocks, but also a considerable variation in temperature from the mammalian host (37˚C) to the insect vector (25-27˚C), which requires membrane fluidity adaptions and remodelling [9–12]. These are some of the reasons why we observe very significant differences in both morphology at a cellular and organellar level, and in the lipid requirements to facilitate these changes between different life cycle stages [9–12]. This is evident in the biosynthesis and uptake of fatty acids (FAs) and in lipid metabolism between life-cycle stages [11,13]. Previous studies in our and others’ groups have shown that BSF and PCF have in fact different FA cellular content and consequently different lipid profiles [14]. In order to allow for FA and lipid remodelling, trypanosomes rely upon both the host for the supply of some of the FA building blocks, and on two essential and highly specialised and tuneable FA synthetic machineries; a mitochondrial fatty acid synthase type II (FASII) and FA elongase-like ketoacyl synthase (ELO) located into the endoplasmic reticulum [8,13,15,16].

Alongside ELO and FASII, *T. brucei* have also evolved a vast repertoire of desaturases and elongases for the *de novo* biosynthesis and remodelling of unsaturated fatty acids (UFAs) and polyunsaturated fatty acids (PUFAs) [17]. Most of these UFAs and PUFAs are normally synthesised from the FAs and lipids that *T. brucei* take up from the host *via* a series of receptor- and/or endocytosis-mediated mechanisms [11,13,18–20]. These include formation of a large amount of linoleic acid (LA) (18:2) and docosapentaenoic acid (DPA) (22:5) and docosahexaenoic acid (DHA) (22:6), and to lesser amounts oleic acid (OA) (18:1) found in BSF, compared to the host environment [13]. The synthesis of these PUFAs is possible through the combined activity of either an initial desaturase, i.e., Δ9-desaturase [21], or front-end desaturases, i.e., Δ6-, Δ5- and Δ4-desaturases [22], or methyl-end desaturases, i.e., Δ12-desaturase [23], as well as elongases (ELO1-4), that are required to work in a concerted and alternating manner with the desaturases to form both omega-6 (ω-6) and omega-3 (ω-3) PUFAs [24,25]. Some of these enzymes have been biochemically characterised (Tb927.7.4180, Tb927.7.4170, Tb927.7.4160, Tb927.5.4530, Tb927.2.3080, Tb927.8.6000) [16,17,21–25], however the way in which *T. brucei* uptake, synthesise and use very long chain PUFAs (VLC-PUFAs) remains not fully understood. RNA-seq studies by Siegel *et al.* and Naguleswaran *et al.* have shown that some of these desaturases are overexpressed both in PCF and stumpy forms compared to BSF of *T. brucei*, including the putative Δ6-desaturase (Tb11.v5.0580) (Tb-Δ6) [26,27]. Kabani *et al* and Silvester *et al* showed that Δ6-desaturase is also overexpressed in the stumpy form of *T. brucei*, alongside the sphingomyelin synthase 1 (SLS-1) [28], deputed to the production of the sphingolipid inositol-phosphoryl ceramide (IPC) [28–31]. SLS-1 is also expressed and observed in PCF, but not in the BSF *T. brucei* [28–30].

Intrigued by these pieces of information, we decided to explore the function and role of Tb-Δ6 in both *T. brucei* PCF and BSF to understand whether the activity of this desaturase might be linked to other lipid/membrane/metabolic changes between these life cycle stages, including the synthesis of IPC. In this study, we discover that this Tb-Δ6 desaturase has a key role in the synthesis of 20C and 22C PUFAs, and that its activity is indeed influenced by the type of environmental FAs available to the parasites. Moreover, we show that by increasing the level of expression of this Δ6-desaturase we can trigger the parasites’ lipid remodelling in response to environmental fat-sources availability, including inducing IPC formation, and other metabolic and proteomic changes, giving rise to cellular morphology changes. Collectively, altering the expression of one gene, namely the Tb-Δ6 desaturase gives new insights on the plasticity of the FA/lipid pathways, which forms some of the essential cascade of metabolic adaptation events during *T. brucei* life-cycle progression between their hosts [2].

## Results

### The fatty acid profile of *T. brucei* PCF and BSF wild type

**Fig 1:**
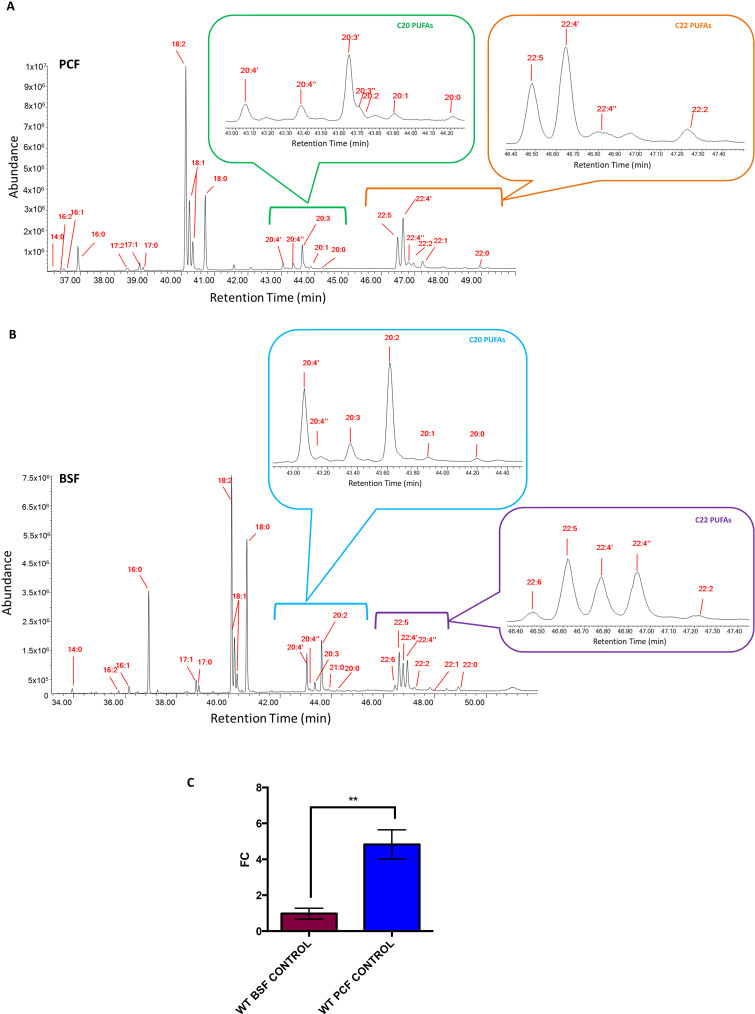
(A, B) GC-MS analysis of the FAMEs in *T. brucei* PCF and BSF. GC-MS chromatogram analysis of *T. brucei* PCF WT cultured for 48 h in SDM-79 with 10% FBS (A) and *T. brucei* BSF WT cultured in HMI-11 with 10% FBS (B). Peaks eluted at different retention times (X axis) and with different abundance (Y axis), are assigned to specific FAs. The C20 (green (A) and light blue (B) brackets and inserts) and C22 PUFAs (orange (A) and purple (B) bracket and insert) are expanded. The red lines indicate the major species of FAs. Note: ‘= first eluted isomer; “= second eluted isomer; first and second eluted isomers are FAs with same number of C on the alkyl chain, and the same number of double bonds in different position along the alkyl chain. The chromatograms are representative of experiment results conducted in three independent biological replicates (n = 3). C) **qRT-PCR of *Tb-*Δ6 *T. brucei* PCF and BSF.** The bar charts show qRT-PCR analysis of *Tb-*Δ*6* expression levels in *T. brucei* PCF and BSF wild type (WT). Values are expressed as fold change (FC) (y axis) calculated from 2^(-ΔΔCt) values and normalized using actin as housekeeping gene. Values are the mean of four technical replicates (n = 4). Error bars represent the standard deviation of each mean. Statistical analysis was performed by GraphPad PRISM 6.0 using One-way ANOVA multiple comparisons based on a Dunnet t-test with a 95% confidence interval, where ** is **p** ≤ 0.01. (S5 Appendix).

It is important to highlight that, when cultured in flasks, trypanosomes rely exclusively on the FBS contained within the media as the primary and only extracellular source of FAs, which are present as free FAs, as well as neutral lipids, phospholipids, and sterol-esters, available for uptake and for *de novo* synthesis. Largely in keeping with previous studies [13], we showed *via* GC-MS that the FA cellular content in *T. brucei* varies significantly between the two main life cycle (BSF and PCF) stages of *T. brucei* (Fig 1A and B respectively). This is remarkable when considering that all the parasites used for our experiments were cultured in media containing the same source of FBS (S1 Table). The most interesting differences were noted in some of the 20C and 22C PUFAs. A different ratio of 20:4, 20:3, 20:2, 22:5, 22:4 and 22:3 was detected between PCF and BSF. Furthermore, the presence of 22:6 was only detected in BSF cells and totally absent in PCF (Fig 1A and B). When reducing the level of FBS in the culture media from a standard level of 10% to 5% for BSF and to 1.25% for PCF, hence reducing the FA availability for uptake, there was no detrimental impact on the growth, however, slightly lower amounts of the 20C and 22C PUFAs were found in both *T. brucei* PCF and BSF (S1 Fig). This suggests that desaturases and elongases are differently expressed and/or used for FA remodelling at different life cycle stages and in response to the level of fat sources available in the environment for uptake. (For detailed analysis, see S2–S7 Figs).

### *T. brucei* desaturase gene sequence identification and level of expression in wild type

To test our hypothesis, we decided to elucidate the role of the unexplored Tb-Δ6 desaturase (Tb11.v5.0580). This enzyme is supposed to be involved in the synthesis of UFAs and/or PUFAs in the two life cycle stages of *T. brucei* [32,33]. The sequence of the gene of interest was retrieved from the Kinetoplastid genome database TriTrypDB [33]. The level of expression of *Tb-*Δ*6* in the PCF and BSF of *T. brucei* was determined by qRT-PCR on RNA extracted from PCF and BSF WT cells used in this study. We were able to confirm that, as reported in the RNAseq data set by Siegel *et al.* and Naguleswaran *et al.* [26,27], the level of expression of *Tb-*Δ*6* is ~5-fold higher (p = 0.0016) in PCF than in BSF WT (Fig 1C).

### Down- and up-regulation of Tb-Δ6 desaturase in procyclic and bloodstream forms of *T. brucei*

**Fig 2:**
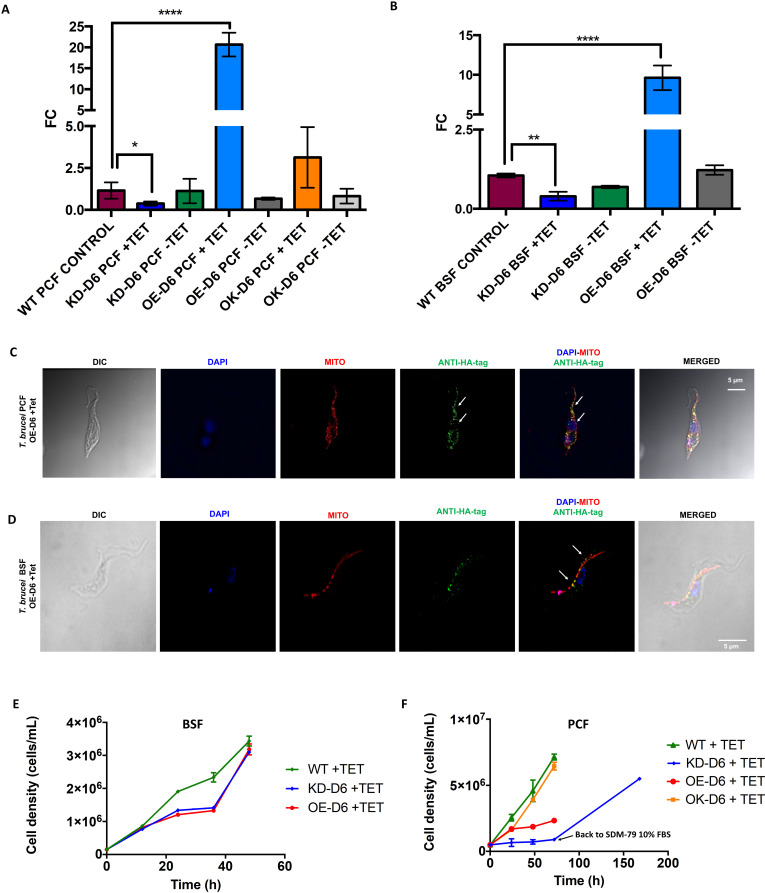
(A, B) qRT-PCR of *Tb-*Δ*6 T. brucei* PCF and BSF confirms the change in the level of expression of *Tb-*Δ*6* in the mutants. The bar charts show qRT-PCR analysis of *Tb-*Δ*6* expression levels in *T. brucei* PCF (A) and BSF (B) wild type (WT), OE-D6, KD-D6 and OK-D6. Values are expressed as fold change (FC) (y axis) calculated from 2^(-ΔΔCt) values and normalized using actin as housekeeping gene. Values are the mean of four technical replicates (n = 4). Error bars represent the standard deviation of each mean. Statistical analysis was performed by GraphPad PRISM 6.0 using One-way ANOVA multiple comparisons based on a Dunnet t-test with a 95% confidence interval, where **** is p ≤ 0.0001, ** is p ≤ 0.01 and * is p ≤ 0.05. (S5 Appendix) **C, D) Tb-Δ6 cellular localisation in *T. brucei* PCF and BSF**. Immunofluorescence microscopy images of *T. brucei* PCF OE-D6 (C) and *T. brucei* BSF OE-D6 (D) fixed on poly-lysine coated slides stained with DAPI (blue signal), MitoTracker Red (red signal), anti-HA tag (green signal) and imaged with DeltaVision Imaging System confocal microscope. The cells were grown for 48 h in HMI-11 with 10% FBS in the presence of tetracycline. The white arrows show that Δ6-desaturase (anti-HA tag green dots) is only partially localised/associated with the mitochondrial compartment (red signal, white arrows). Images were processed using softWoRx Explorer 1.3. **E, F) Growth curves of *T. brucei* BSF and PCF**. The graphs represent the growth curves over 48 h of *T. brucei* BSF (E) and over 72 hours of PCF (F) WT controls and *T. brucei* BSF (E) and PCF (F) Tb-Δ6 knock-down (KD-D6), Tb-Δ6 overexpression (OE-D6) and Tb-Δ6 add-back (OK-D6) cells, when they are cultured in HMI-11 supplemented with 5% FBS (E) or in SDM-79 supplemented with 1.25% FBS (F) in the presence of tetracycline as shown in the legend. For all growth curves values are the mean of three independent biological replicates (n = 3). Error bars represent the standard deviation of each mean. Statistical analysis was performed by GraphPad PRISM 6.0 using One-way ANOVA multiple comparisons based on a Tukey t-test with a 95% confidence interval.

To investigate how the level of expression of Tb-Δ6 impacts the FA cellular content in different life cycle stages of *T. brucei*, the overexpression (OE-D6) and the knock-down (KD-D6) cell lines were generated in both *T. brucei* PCF and BSF. Tb-Δ6 expression was down-regulated *via* the tetracycline (Tet)-inducible RNA interference (RNAi) p2T7-177 vector (KD-D6) [34,35], and upregulated *via* the Tet-inducible pLew100 vector (OE-D6) [36,37]. Alongside these cell lines, an add-back (OK-D6) cell line was also generated in PCF, by transfecting KD-D6 cells with the pLew100 vector. The stable integration of the transfected DNA in the genomic DNA was confirmed by PCR (S8A–D and S9A–F Figs). Furthermore, the expected changes in the *Tb-*Δ*6* mRNA levels for the overexpression, knock-down and add-back (only for PCF) of Tb-Δ6 with and without Tet-induction, compared to the WT (BSF and PCF) controls, were observed by qRT-PCR (Fig 2A and 2B). Particularly, the level *Tb-*Δ*6* mRNA in Tet-induced OE-D6 was ~20-fold and ~10-fold higher than the WT in PCF and BSF, respectively (p < 0.0001) (Fig 2A–B). On the other hand, *Tb-*Δ*6* mRNA level in Tet-induced KD-D6 was ~0.4-fold lower than the WT in both PCF (p = 0.0148) and BSF (p = 0.0011) (Fig 2A and B). As expected, no significant difference was observed for both induced and non-induced OK-D6 PCF compared to the WT (Fig 2A and B). No differences were observed in any of the non-induced cell lines, as expected (Fig 2A and B).

### Tb-Δ6 is associated with the mitochondrial compartment of *T. brucei* PCF and BSF

The cellular location of Tb-Δ6 was also investigated using the Tet-inducible overexpression vector pLew100, which expresses a C-terminal-HA-tag on the Tb-Δ6 protein. OE-D6 and WT cells for both PCF and BSF were harvested after 48 h of Tet-induction. Initial analysis by immunoblotting showed that Tb-Δ6 is expressed in both PCF (S10A Fig) and BSF (S10B Fig) and migrated according to the predicted size (~49 kDa). The Tb-Δ6-HA protein was then detected by immunofluorescence microscopy using an anti-HA secondary antibody in the presence of MitoTracker Red and DAPI stains. The green, fluorescent punctate signals from Tb-Δ6-HA and the red fluorescence of the stained mitochondria showed a partial overlapping distribution in both PCF (Figs 2C and S10C) and BSF (Figs 2D and S10D) cells. It is possible to speculate that Tb-Δ6 may be partially localised/associated with parts of the mitochondria.

### Knock-down and overexpression of Tb-Δ6 alter the growth rate depending upon the level of fatty acids available from Foetal Bovine Serum (FBS) in the media

Growth curves were performed to evaluate any significant effect of Tb-Δ6 genetic manipulation on *T. brucei* PCF and BSF cell proliferation. After 48 h in HMI-11 supplemented with 10% FBS, BSF WT and mutants showed no significant differences in growth rate either in the presence (S11A Fig) or absence (S11B Fig) of Tet. The same was observed for PCF WT and mutants cultured for 72 h in SDM-79 supplemented with 10% FBS (S11C and S11D Fig). However, significant differences were observed between the WT and Tb-Δ6 desaturase mutants in PCF and BSF in low-fat media (5% FBS for BSF and 1.25% FBS for PCF). Both the OE-D6 and KD-D6 BSF mutants revealed a slower cell growth over the first 24 h ( ~2-fold slower than the WT) in the presence of Tet (Fig 2E). At this point, the growth reached a plateau, that was maintained for 12 h (Fig 2E). This lag and static growth phases were reproducibly observed numerous times. After a further 12 h, these cells returned to a growth rate similar to those of the WT (Fig 2E). The differences in the growth rate were even more evident in PCF. OE-D6 PCF showed a slower growth rate ( ~3-fold) compared to the WT (Fig 2F). While KD-D6 PCF mutants showed a very slow and almost stationary growth ( ~8-fold lower than WT, p < 0.0001) over the observed time period (Fig 2F). No significant differences were observed between the cell growth of WT control and OK-D6 PCF under induction of Tet (Fig 2F), suggesting that the WT phenotype had been efficiently rescued from the initial knock-down of Tb-Δ6. This phenomenon was also observed when KD-D6 PCF cells were transferred back to SDM-79 with 10% FBS (Fig 2F). The cells were able to reach a final cell density similar to the one of OK-D6 and WT control after 4 days (Fig 2F). No significant differences were observed for both BSF (S11E Fig) and PCF (S11F Fig) cell lines in absence of Tet in low-fat media.

### The PUFAs content of *T. brucei* PCF and BSF changes depending upon the level of Tb-Δ6-desaturase activity and the fatty acid sources in the media

**Fig 3:**
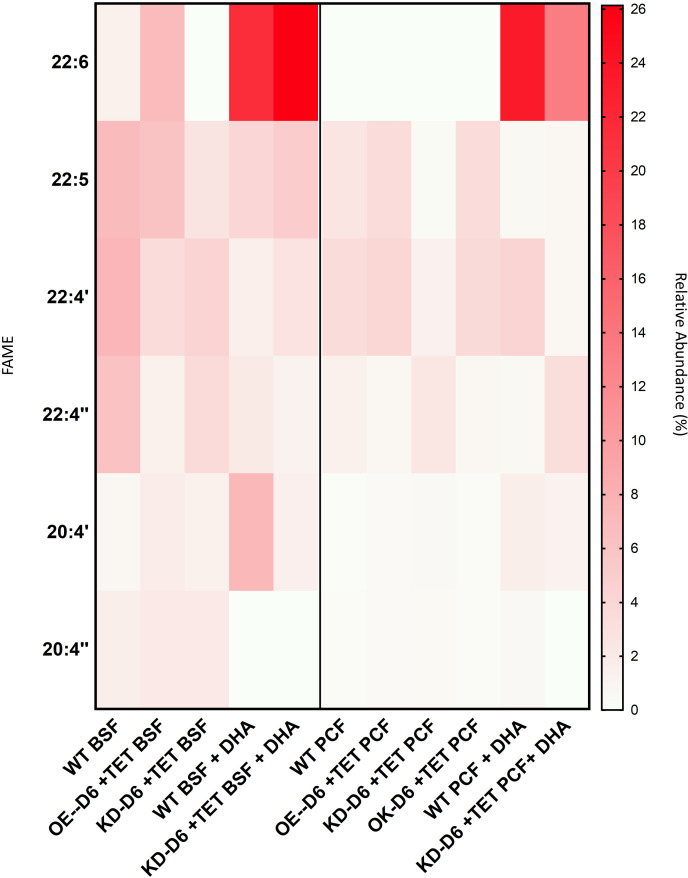
Comparison of 20C and 22C PUFAs between Tb-Δ6 genetically manipulated *T. brucei*PCF and BSF in high- and low-fat media and under DHA supplementation. The heat map aims to give a visual summary of the difference in 22:6, 22:5, 22:4 and 20:4 PUFAs comparing the total relative abundance of each FA between Tb-Δ6 genetically modified *T. brucei* BSF (KD-D6 and OE-D6) (left) and *T. brucei* PCF (KD-D6, OE-D6, OK-D6) (right) and WT controls, when the cells are cultured for 48 h in low-fat media or in low-fat media supplemented with 10 µM DHA (22:6), in the presence of Tet. All values are taken from S3, S6, S13 and S14 Figs. Values are the mean of three independent biological replicates (n = 3). Note: ‘ = first eluted isomer; “ = second eluted isomer. (S1 and S2 Appendices).

To investigate the effect on total FA cellular content in the Tb-Δ6 knock-down and overexpression cells, GC-MS analysis was carried out on fatty acid methyl esters (FAMEs) from lipid extracts of KD-D6, OE-D6, OK-D6 *T. brucei* PCF and KD-D6 and OE-D6 *T. brucei* BSF grown in both high- and low-fat media in the presence and absence of Tet (S2–S7 Figs). The most significant changes were observed in the relative abundance of 20:4, 22:4, 22:5 and 22:6 PUFAs, when comparing the WT control with the mutants in both *T. brucei* PCF and BSF, and especially in low-fat (1.25% FBS for PCF or 5% FBS for BSF) media in the presence of Tet (Figs 3, S3 and S5). In PCF KD-D6 mutants, we detected increasing amounts of 20:4’ (4-fold, ns) and of 22:4” (1.7-fold, p = 0.0300) (Figs 3, S3B and S3C), as well as a decrease of 22:5 (6.8-fold, p = 0.0293) compared to the WT control (Figs 3 and S3C). Oppositely, PCF OE-D6 mutants showed a slight increase of 22:5 compared to the WT (1.4-fold, ns) (Figs 3 and S3C), and a consequent decrease of 22:4” (2.4-fold, ns) (Figs 3 and S3C). As expected, OK-D6 mutants did not show any significant difference compared to the WT control (Figs 3 and S3C). There were no significant differences between the WT control and the mutants in the absence of Tet (S6B Fig).

In BSF KD-D6 increasing levels of 22:4” (2.6-fold, p = 0.0251) were observed compared to OE-D6 cells (Figs 3 and S5C). Decreasing amounts of 22:5 in BSF KD-D6 mutants were observed compared to OE-D6 cells and the WT control (2.5-fold, p = 0.0251; 2.6-fold, p = 0.0046), respectively (Figs 3 and S5D). Meanwhile, both isomers of 22:4 were lower (4.3-fold, p < 0.0001; 2-fold, p = 0.0004) in BSF OE-D6 mutants than in the WT control (Figs 3 and S5C) but showed an increase of 22:6 (5.0-fold, p = 0.0003) compared to the WT control (Figs 3 and S5E). Interestingly, 22:6 was not detected in KD-D6 BSF mutants compared to the WT control and OE-D6 mutants (p < 0.0001) (Figs 3 and S5E).

### The ratio of product versus substrate of Tb-Δ6 desaturase changes with the level of expression of Tb-Δ6

**Fig 4:**
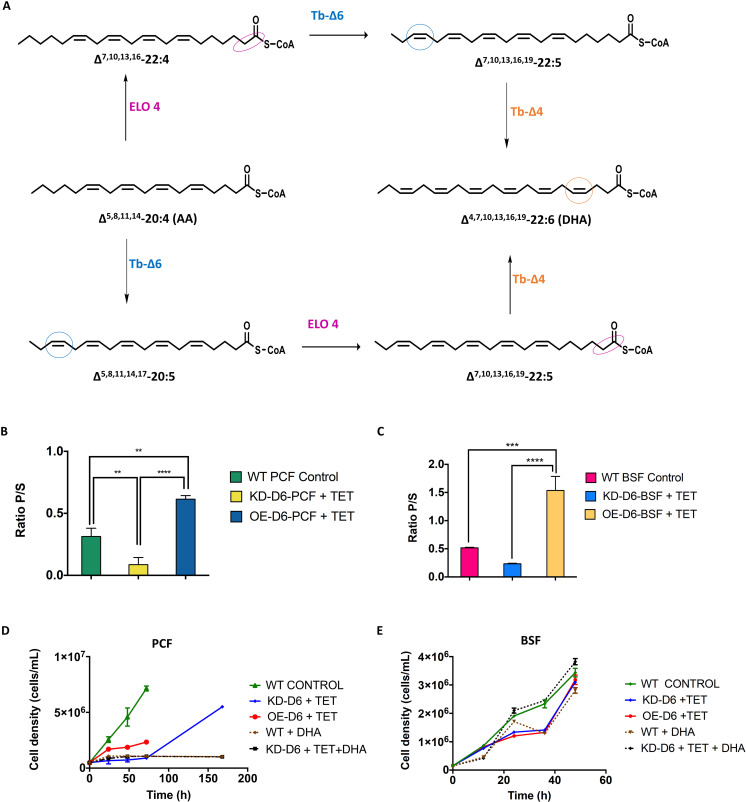
A) Biotransformation of 20C and 22C PUFAs involving Tb-Δ6. The reaction scheme depicts the biotransformation pathway for the synthesis of 20C and 22C PUFAs in *T. brucei*. The blue circles highlight the insertion of the double bond by Tb-Δ6 (blue). The orange circle highlights the insertion of the double bond by Tb-Δ4 (orange). The magenta circles highlight the elongation of two methylene groups by Tb-ELO4 (ELO 4 magenta). **B, C) The ratio between products (P) and substrates (S) of the biotransformation of 20C and 22C PUFAs in Tb-Δ6 genetically modified *T. brucei* PCF and BSF in low-fat media.** The bar charts show the ratio between the total amount of hypothetical products (P) 22:5 and 22:6 PUFAs versus the total amount of the substrates (S) 20:4 and 22:4 PUFAs produced by Tb-Δ6 genetically modified *T. brucei* PCF (B) and BSF (C) (KD-D6 and OE-D6) and WT controls cultured for 48 h in SDM-79 with 1.25% FBS and HMI-11 with 5% FBS, respectively. All values are the mean of three independent biological replicates (n = 3). Error bars represent the standard deviation of each mean (±). All FAs were identified using GC-MS based upon retention time, fragmentation, and comparison with standards. Statistical analysis was performed by GraphPad PRISM 6.0 using 2-ways or One-way ANOVA multiple comparisons based on a Tukey t-test with a 95% confidence interval, where **** is p ≤ 0.0001, *** is p ≤ 0.001, ** is p ≤ 0.01 and * is p ≤ 0.05. **D, E) Growth curves of Tb-Δ6 genetically modified *T. brucei* PCF and BSF supplemented with DHA.** The graphs represent the growth curves over 72 h of *T. brucei* PCF (D) and over 48 h of BSF (E) WT control and *T. brucei* PCF Δ6-desaturase knock down (KD-D6), when they are cultured in SDM-79 supplemented with 1.25% FBS (D) and HMI-11 supplemented with 5% FBS (E) (solid lines), and in SDM-79 supplemented with 1.25% FBS (D) and HMI-11(E) supplemented with 5% FBS added with 10 µM DHA (22:6) (dotted/dashed lines), in the presence of tetracycline as shown in the legend. For all growth curves values are the mean of three independent biological replicates (n = 3). Error bars represent the standard deviation of each mean. Statistical analysis was performed by GraphPad PRISM 6.0 using One-way ANOVA multiple comparisons based on a Tukey t-test with a 95% confidence interval. Note: the solid lines represent data taken from Fig 2E and 2F used here for a more complete comparison.

The trend of increasing amounts of 20:4 and 22:4 in the knock-down cell lines, and of increasing amounts of 22:5 and 22:6 in the overexpression mutants, and the vice versa, led to the hypothesis that 22:5 and/or 22:6 might be the products (P) and 20:4 and/or 22:4 the substrates (S) of a multistep elongation-desaturation biotransformation, involving the Tb-Δ6 (Fig 4A). To test this hypothesis, the ratio of products (sum of 22:5 and 22:6) (P) and substrates (sum of 20:4 and 22:4) (S) was calculated for PCF and BSF in low-fat media (Fig 4B and 4C).

These results clearly show that in both PCF and BSF to a  higher level of expression of Tb-Δ6 corresponds a higher level of products, i.e., 22:5 and 22:6, (2-fold, p = 0.0011; 3-fold, p = 0.0004) respectively; whereas to a lower level of expression of Tb-Δ6 corresponds a higher level of substrates, i.e., 20:4 and 22:4 (3.2-fold, p = 0.0049; 2.2-fold, ns respectively). This observation was supported by the significant increase in P/S detected in both PCF and BSF OE-D6 cells compared to KD-D6 cells (6.2-fold, p = 0.0001; 6.7-fold, p < 0.0001 respectively) (Fig 4B and 4C).

### Growth rate of *T. brucei* PCF and BSF after supplementation with docosahexaenoic acid

In order to elucidate the substrate(s) versus product(s) ratio, we decided to supplement the low-fat media with the potential final product involving the Tb-Δ6 transformation, i.e., docosahexaenoic acid (Δ4,7,10,13,16,19-22:6) (DHA), to try and rescue the growth defects observed in both PCF and BSF KD-D6 mutants (Figs 4D, 4E, S12A and S12B). After 72 h with DHA supplementation, both in the presence and absence of Tet, KD-D6 PCF mutants and the PCF WT control showed an unexpected total lack of growth (Figs 4D and S12A). The cells were not able to restore a normal growth rate even after 6 days of being cultured under these conditions. This was in contrast with what observed in the initial experiments, where KD-D6 PCF cells restored their growth when transferred back into a fat-rich media after several days (Fig 2F), noting that the FBS does not contain DHA (serum does contains 20:4, but none with a higher degree of unsaturation nor longer fatty acids). This clearly indicates that PCF growth is incompatible with relative minor concentrations of DHA.

In contrast, KD-D6 BSF supplemented with DHA rapidly reached a cell density equal to the one of the non-supplemented WT control, after dividing for 12 h at a slower rate (Fig 4E). KD-D6 mutants maintained this growth for a further 24 h (Fig 4E). After a 48-hour period of adaptation, all BSF cell lines were able to reach a very similar cell density (Figs 4E and S12B).

### The PUFA content in *T. brucei* PCF and BSF after supplementation with docosahexaenoic acid

The PUFA content of the various cells were also analysed after DHA supplementation. Despite the lag in growth registered for PCF WT and KD-D6 cells, they internalised DHA (Figs 3 and S13). The relative abundance of 22:6 in the WT cells was ~23% (p < 0.0001), compared to the ~13% in KD-D6 cells in the presence of Tet (p < 0.0001) and 8% in the absence of Tet (p < 0.0001) (Figs 3 and S13B). Due to the high amount of DHA internalised, both WT and KD-D6 PCF showed a reduction of 22:5 compared to non-supplemented OE-D6 and WT (p = 0.0201; p = 0.0027), respectively (Figs 3 and S13E). For the same reason also 22:4’ decreased in KD-D6 PCF compared to WT control (ns) (Fig 3 and S13D). The WT PCF supplemented with DHA showed very similar levels of both isomers of 22:4 compared to non-supplemented OE-D6 PCF, as well as an increase of 20:4’ (Figs 3 and S13C).

Both WT and KD-D6 BSF internalised DHA, which was detected at a relative abundance between ~ 20-25% (Figs 3 and S14B). Once again, due to the excess of DHA both WT and KD-D6 BSF showed a reduction of 22:5 compared to OE-D6 and WT (Figs 3 and S14E) (p = 0.0171; p = 0.0305), but this was less significant for the knock down than the one observed in PCF. This was also true for both species of 22:4, however the reduction was more evident in KD-D6 BSF cells supplemented with DHA (Figs 3 and S14D). Interestingly, the WT and KD-D6 BSF cells supplemented with DHA had a higher level of 20:4’ compared to non-supplemented OE-D6 and WT (p < 0.0001), respectively, which is in keeping with the presence of lower level of longer chain PUFAs (22:4 and 22:5) (Figs 3 and S14C). On the other hand, 20:4” was not detected (p < 0.0001) (Figs 3 and S14C). It is also important to highlight that, under DHA supplementation, PCF and BSF WT had lower level of 18:1 (p < 0.0001; p = 0.0202) and 18:2 (ns in BSF; p = 0.0096 for PCF), while presenting higher levels of 18:0 compared to PCF and BSF WT cells cultured in 10% FBS, particularly in PCF (ns in BSF; p = 0.0135 for PCF) (S15 Fig). This is a clear mechanism adopted by the cells to compensate for the high amount of PUFAs, not normally present in FBS (S1 Table), but available under DHA supplementation, when Tb-Δ6 is expressed at normal levels.

This suggests that both BSF and PCF cells are able to internalise the DHA, causing subsequent alteration in the relative abundances of monounsaturated fatty acids (MUFAs) and PUFAs, causing detrimental effects to PCF cell growth, but not that of BSF, which normally contains DHA.

### Overexpression of Tb-Δ6 desaturase causes unexpected production of IPC in BSF*T. brucei*

**Fig 5:**
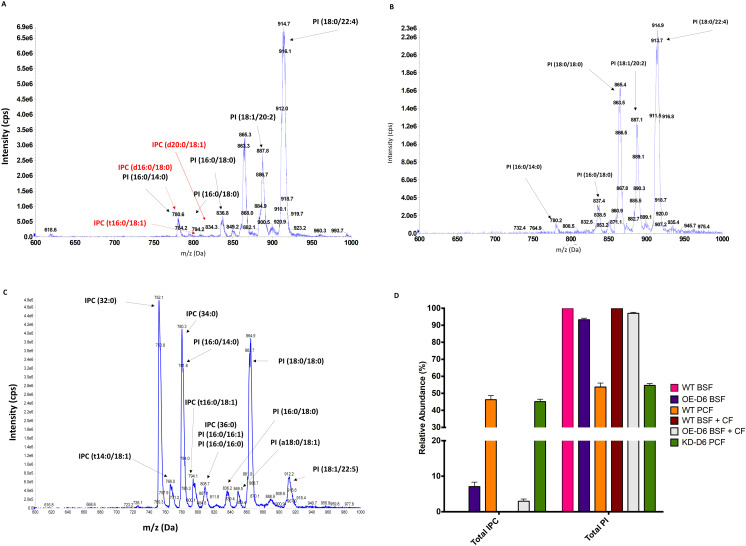
A.B,C) ESI-MS/MS of PI-containing lipids from genetically manipulated *T. brucei* BSF (A), *T. brucei* BSF WT (B) and *T. brucei* PCF WT (C) all grown in low-fat media. The spectra show PI-containing lipids and IPCs obtained by scanning for parent ion of 241 m/z for OE-D6 BSF grown for 48 h in HMI-11 with 5% FBS in the presence of Tet (A), for BSF WT grown for 48 h in HMI-11 with 5% FBS (B) and for PCF WT grown for 48 h in SDM-79 with 1.25% FBS (C). The species of interest are labelled as reported in the text and highlighted by arrows. Note: d, sphingosine; t, 1,2,3-trihydroxy sphingosine. (S4 Appendix) Spectra are representative of experiments conducted in three independent biological replicates (n = 3). **D) ESI-MS/MS quantification of total IPCs and total PIs fromTb-Δ6 genetically manipulated and WT *T. brucei* BSF and PCF in low-fat media.** The bar chart shows the relative abundance (%) (y axis) of the total amount of IPC and PI (X axis) detected in OE-D6 BSF, WT BSF, KD-D6 PCF and WT PCF. The bar chart also shows the relative abundance (%) (y axis) of the total amount of IPC and PI (X axis) found in OE-D6 BSF and WT BSF after treatment with EC_10_ (0.61 µM calculated with GraphPad from the EC_50_) of clemastine fumarate (CF), as shown in the legend. Tb-Δ6 genetically modified *T. brucei* BSF and PCF and WT control are cultured for 48 h in HMI-11 supplemented with 5% FBS and in SDM-79 with 1.25% FBS, respectively, in the presence of tetracycline where required. Values are the mean of three independent biological replicates (n = 3). Error bars represent the standard deviation of each mean. (S4 Appendix).

The differences in the levels of 20C and 22C PUFAs normally has an impact on the composition of the lipids within the cellular membrane, with an effect on its fluidity [32]. Thus, the parasites must adapt/modify their lipid pool to maintain the correct membrane fluidity, under these conditions. To investigate this, lipidomic studies were carried out by electrospray ionization tandem mass spectrometry (ESI-MS/MS) [14]. The differences in the lipid species between WT control and KD-D6 and OE-D6 mutants were analysed in both PCF and BSF at high- and low-fat media in the presence of Tet. Parent ion scanning of 241 m/z (inositol-1,2-cyclic phosphate collision induced fragment) revealed significant changes in the intensity of some peaks and some new peaks, attributed to IPCs in the range 780-840 m/z, in the induced OE-D6 BSF cells in low-fat media (Figs 5A and S16A–D) compared to the WT control (Fig 5B) and KD-D6 cells (S16A Fig). These were identified through fragmentation as IPC (d16:0/18:0) at 780 m/z (Figs 5A and S17A), IPC (t16:0/18:1) at 794 m/z (Figs 5A and S17B) and IPC (d20:0/18:1) at 834 m/z (Figs 5A and S17C). This was surprising, as IPC biosynthesis has only been observed in stumpy and PCF *T. brucei.* Therefore, increasing the expression level of Tb-Δ6, which alters the PUFAs composition, leads to changes in the phospholipid membrane composition, including the production of IPC. These are two biochemical alterations that are typical of stumpy BSF *T. brucei*.

Quantification of IPC and PI present in OE-D6 and WT BSF were compared with KD-D6 and WT PCF. As expected, WT PCF cells contain around 50% of IPC and 50% of PI (Fig 5C and 5D) and WT BSF contain only PI (100%) (Fig 5D) [14]. OE-D6 BSF mutants showed formation of IPC (7%), (while in stumpies, IPC accounts for ~20%) [28], and a reduction of PI (Fig 5D), in line with the redirection of the PI head group to facilitate IPC synthesis (S16E Fig). As IPC synthesis also requires ceramide, the levels of sphingomyelin (SM), was investigated for a reduction, as previously observed in PCF (S16E Fig) [14,28–30,38]. Parent ion scanning of 184 m/z (phosphocholine collision induced fragment), showed a reduction of SM (d16:0/18:0) at 706 m/z (identification confirmed by fragmentation S17D Fig) in OE-D6 mutants compared to the WT control (S18 Fig). Surprisingly, when knocking down Tb-Δ6 in PCF the 50:50 ratio between IPC and PI was maintained constant (Fig 5D), but an overall reduction of both IPC and PI species was observed (S16F Fig).

### Clemastine fumarate reduces IPC production in *T. brucei* BSF overexpressing Tb-Δ6 desaturase in reduced fat media

To assess whether the IPC production by OE-D6 BSF mutants in low-fat media could be reduced/inhibited, we utilised the leishmanial and *T. cruzi* IPC synthase inhibitor clemastine fumarate [39,40]. Clemastine fumarate was tested in a dose-response assay against BSF WT, EC_50_ 6.76 ± 0.15 µM (S19A Fig), and OE-D6 BSF induced and non-induced EC_50_ 5.45 ± 0.30 µM (S19B Fig) and EC_50_ 6.89 ± 0.26 µM (S19C Fig) respectively. Treatment of clemastine fumarate at a non-lethal dose (EC_10_ 0.61 µM) against Tet induced OE-D6 BSF showed that the level of IPC was significantly reduced towards no IPC as in WT BSF (Fig 5D). This clemastine fumarate inhibition of IPC formation in Tet induced OE-D6 BSF should allow the ceramide to be re-utilised for SM synthesis. However, this was not the case as the SM (d16:0/18:0) was 1.4-fold lower in treated and induced OE-D6 cells, and it was also 1.4-fold lower in treated WT cells compared to the WT untreated control (S18D Fig).

### The effect on cellular morphology due to overexpression of Tb-Δ6

**Fig 6:**
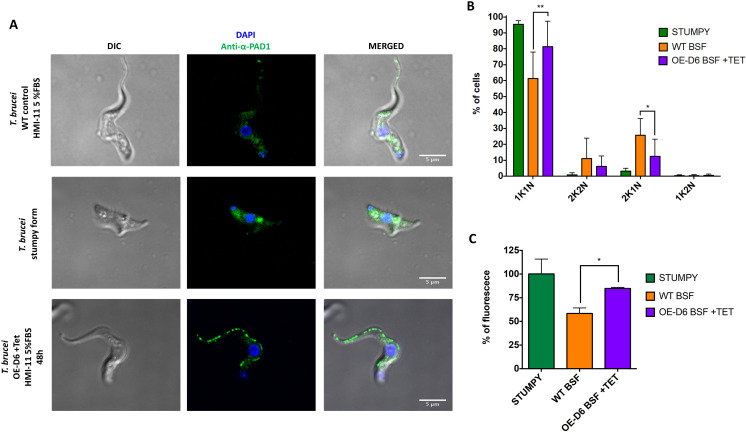
T. brucei BSF cell morphology changes when the cells overexpress Tb-Δ6 in low-fat and produce IPC. Immunofluorescence microscopy images of *T. brucei* BSF WT slender  form (first row), * T. brucei* stumpy form (positive control) (second row) and *T. brucei* BSF OE-D6 (third row) fixed on poly-lysine coated slides, stained with DAPI (blue signal) and anti-α-PAD1 antibody (green signal) and imaged with DeltaVision Imaging System microscope. The control cells were grown for 48 h in HMI-11 with 5% FBS. OE-D6 were grown in the same conditions in the presence of tetracycline for 48 h. Stumpy cells were grown in HMI-11 with 20% FBS and differentiated using 15% BHI for 48 h. Images were processed using ImageJ software. Quantification of the fluorescence signal was carried out using Volocity 6.0 software. All values are the mean of three (B, n = 3, number of cells = 50-80) or two (C, n = 2, number of cells = 50-120) independent biological replicates. Error bars represent the standard deviation of each mean (±). Statistical analysis was performed by GraphPad PRISM 6.0 using 2-ways multiple comparisons based on a Tukey t-test with a 95% confidence interval, where ** is p ≤ 0.01 and * is p ≤ 0.05. (S8 and S9 Appendices).

Thus far, the data implies that higher levels of Tb-Δ6 expression in BSF, under reduced fat conditions, affects the lipid composition of the membranes, resulting in the formation of IPC. These factors must be inter-connected by means of a cascade of biochemical responses, which are normally associated with the progression from one life-cycle stage to the other, i.e., BSF slender to stumpy. BSF overexpressing Tb-Δ6 also show distinct cellular morphology changes, appearing with a distinctly “plumpy” morphology at 48 hours, with an enlarged/flattened cell morphology and shortened flagellum, which again resembles/anticipate the stumpy morphology [28,41]. (Fig 6A).

Additionally, their cell growth shows that there is hardly any cell-division between the 24h and 36h timepoints (Fig 2E). This was confirmed by microscopy as nearly 80% of the cell population analysed (n = ~50) after 48h under Tet-induction were found to be in the non-dividing 1K1N stage, which is typical characteristic of the non-proliferative stumpy cells (Fig 6B) [42,43]. The other 8% of the cells were found to be 2K2N, and ~12% in 2K1N and less than 1% in 1K2N (Fig 6B) [42,43].

To investigate the possibility of the presence of other stumpy markers, immunofluorescence microscopy of the protein associated with differentiation 1 (PAD1), a known molecular transducers of differentiation signals known to be highly expressed in the stumpy forms, was performed [44]. In keeping with studies by Matthews *et al*., stumpy parasites (BHI induced to pleomorphs), were used as a positive control and showed their typical shorten and flattened shape, as well as an intense signal for PAD1 (Fig 6A and 6C) [44]. Interestingly, the WT negative control slender form of monomorphic trypanosomes also showed a weak, but still visible signal for PAD1, however significantly lower than the stumpy control (Fig 6A and 6C) [28,44]. The induced OE-D6 cells showed significantly higher level of fluorescence (peripheral areas) than the WT, but slightly lower than stumpies (Fig 6A and 6C). Interestingly, the staining for PAD1 revealed a tubular punctuated pattern in the induced OE-D6 cells, which is quite distinct from the more diffuse signal in stumpies (Fig 6A).

This strongly implies that the higher expression levels of Tb-Δ6 actively participate in a cascade of events that ultimately causes the morphological and biochemical transitions from BSF slender to plumpy “stumpy-like” cells, i.e., halfway between slender and stumpy morphologies.

### Proteomics analysis *T. brucei* BSF overexpressing Tb Δ6 desaturase in reduced fat media

**Fig 7:**
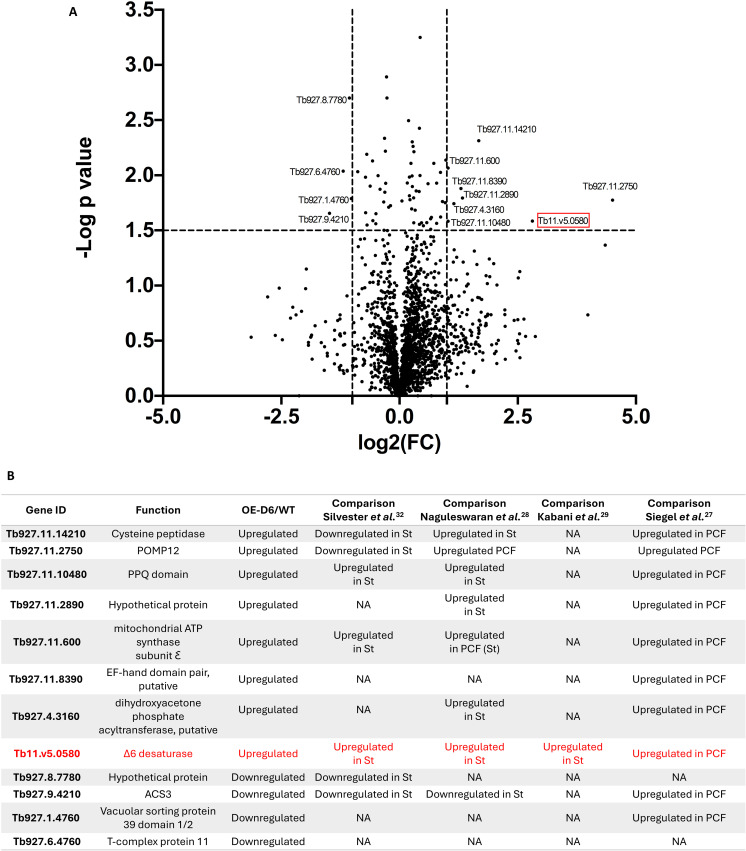
SWATH proteomics analysis between *T. brucei* BSF overexpressing Tb-Δ6 in low-fat media and the WT control. A) The volcano plot shows a range of proteins that are differentially expressed between *T. brucei* BSF overexpressing Tb-Δ6 (OE-D6) and the WT control cultured in HMI-11 supplemented with 5% FBS for 48 h, in the presence of tetracycline where required. log2FC is plotted on the x-axis and the −log10 of the p value on the y-axis. The vertical dotted lines indicate a fold change >1.0 and the horizontal dotted line a p value < 0.05. All values are the mean of three independent biological replicates (n = 3). Data were processed using Perseus software to obtain values of log2FC and −log10 of the p value using a Two-ways t-test. B) The table summarises the list of the most significantly up- and downregulated proteins (Gene ID) in OE-D6, their function, and a comparison with the level of their transcript expression in differentiation studies from Silvester *et al.*, Kabani *et al.*, Naguleswaran *et al.* and Siegel *et al*. [26–28,31] Note: NA, data comparison not applicable as gene ID was not found in the data set; St, stumpy; (S6 Appendix).

Quantitative SWATH whole cell proteomics analysis was carried out to investigate if other proteins that are known to be differentially expressed in the stumpy and PCFs (compared to BSFs) were also up-/ down-regulated in these OE-D6 BSF cells. A volcano plot (Fig 7A) clearly shows a range of proteins that are differentially expressed between WT and Tet induced OE-D6 BSF. Firstly, we looked for the presence of SLS1 (responsible for IPC synthesis) in OE-D6 BSF. It is important to highlight that all four sphingolipid synthases share a 95% sequence homology [29,45]. Through SWATH analysis, a highly conserved peptide among SLS1-4 (VTKPLPDLGFELLTK) was detected. Two peptides (SEELDMNGVLEGR; HGGVDGDEALMFK) specific to SLS4 (Tb927.9.9380) were also identified, confirming the presence of SM synthase in the BSF of *T. brucei* (S20A Fig). Furthermore, a peptide (EVTEDSQPVMVA) specific to SLS1 sequence (Tb927.9.9410) was also detected, confirming its presence, hence the formation of IPC (S20B Fig). However, due to their low-abundance in the cellular protein extracts, the data could not be efficiently used for comparative quantitative analysis between WT and OE-D6 BSF cells [45]. Nonetheless, we were able to confirm the successful overexpression of Tb-Δ6 (Tb11.v5.0580) (Fig 7A). Several other differential regulations of other proteins in OE-D6 BSF cells compared to the WT control were observed, that have also been observed from previous differentiation studies on stumpy form and/or PCF of pleomorphic *T. brucei* [26–28,31]. (Fig 7B). The putative cysteine peptidase (Tb927.11.14210) was upregulated in OE-D6 cells (Fig 7A and 7B). An outer mitochondrial membrane transport protein (POMP12) (Tb927.11.2750) was also significantly upregulated in OE-D6 cells (Fig 7A and 7B) [46]. Furthermore, the mitochondrial ATP synthase subunit Ɛ (Tb927.11.600), which is part of the F0F1-ATP machinery, which is highly expressed in the PCF, and was also upregulated in OE-D6 cells (Fig 7A and 7B) [47,48]. Furthermore, a dihydroxyacetone phosphate acyltransferase (Tb927.4.3160), which is highly expressed in stumpy and PCF, was also overexpressed in OE-D6 cells (Fig 7A and 7B) [49]. The PPQ protein (Tb927.11.10480), was also upregulated in OE-D6 cells (Fig 7A and 7B) [50], as was an EF-hand domain pair (Tb927.11.8390) [51–53]. Only four proteins were found to be significantly down regulated: an hypothetical protein (Tb927.8.7780), a T-complex protein 11 (Tb927.6.4760), a vacuolar sorting protein 39 domain 1/2 (Tb927.1.4760) [54] and the fatty acyl-CoA synthetase 3 (ACS3) (Tb927.9.4210) [33].

### Metabolomics analysis of *T. brucei* BSF overexpressing Tb Δ6 desaturase in reduced fat media

**Fig 8:**
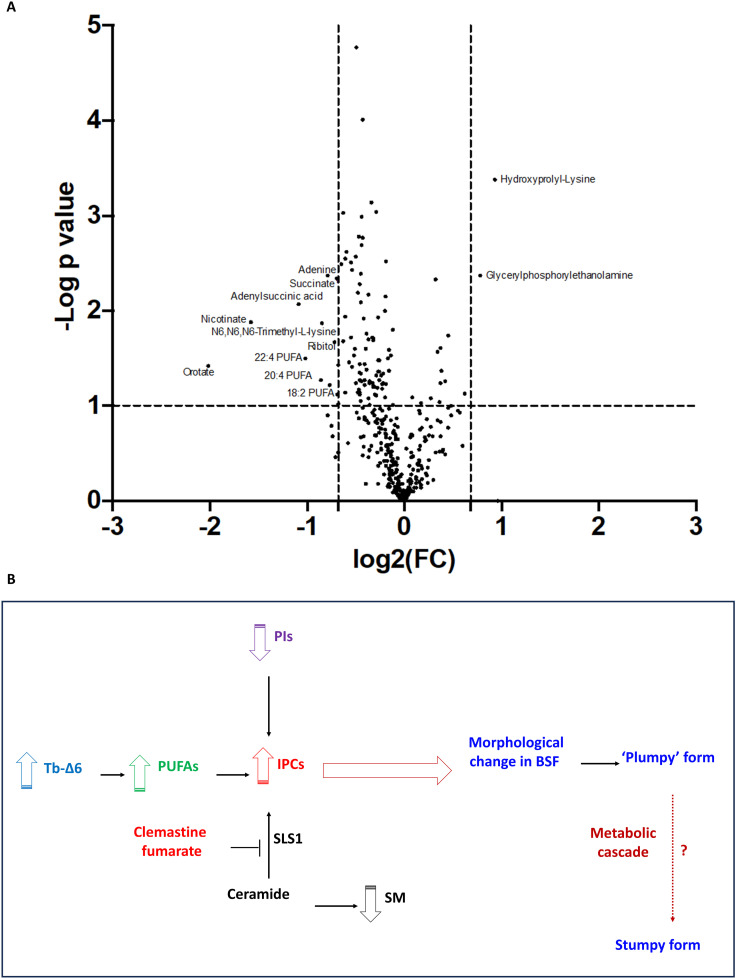
Metabolomics analysis and summary of mechanism of production of IPC in *T. brucei* BSF overexpressing Tb-Δ6 in low-fat media. A) The volcano plot shows a range of cellular metabolites that are differentially detected between *T. brucei* BSF overexpressing Tb-Δ6 (OE-D6) and the WT control cultured in HMI-11 supplemented with 5% FBS for 48 h, in the presence of tetracycline where required. log2FC is plotted on the x-axis and the −log10 of the p value on the y-axis. The vertical dotted lines indicate a fold change >1.0 and the horizontal dotted line a p value < 0.07. All values are the mean of three independent biological replicates (n = 3). Data analysis was performed using the Polyomics Integrated Metabolomics Pipeline (PiMP). Data were processed using Perseus software to obtain values of log2FC and −log10 of the p value using a Two-ways t-test. (S7 Appendix) B) The scheme shows the mechanism for which, in presence of higher expression level of Tb-Δ6 (light blue), OE-D6 BSF trypanosomes produce higher level of PUFAs (green). This causes production of IPC (red) by SLS1 and a consequent reduction on PIs (purple) and SM (black), while redirecting the ceramide anchors. The synthesis of IPC by SLS1 cascade can be inhibited by clemastine fumarate. These events determine an alteration in morphology, and a shift of the cells (empty brown arrows) towards a ‘plumpy’ form. A potential cascade of metabolic events (dotted arrows) might lead the morphological changes into the stumpy pre-infective form.

Untargeted metabolomic analysis was also carried out to analyse the cellular metabolites of Tet-induced OE-D6 cells compared to the WT control in low-fat media. A volcano plot (Fig 8A) shows the most significant metabolite changes. These metabolite changes include a range of PUFAs, as you might expect, but also a significant increase in glycerophosphorylethanolamine [14,38]. A significant reduction of nicotinate and orotate, which are involved in pyrimidine biosynthesis, along with a reduction of adenine and adenyl-succinate, impacting purine biosynthesis, were observed [55–57]. A significant decrease in N6, N6, N6-Trimethyl-L-lysine was also detected [58]. Interestingly, we observed a reduction of succinate [47] and an increase in dipeptide levels, such as those of hydroxyprolyl-lysine [7,14,38,59]

## Discussion

In this study, we elucidated the essential role of Tb-Δ6 in the biosynthesis of PUFAs in *T. brucei* PCF and BSF both *via* genetic manipulation and complementation of the level of essential fat sources in the media [60,61]. Importantly, we confirmed that Tb-Δ6 is differentially expressed (significantly higher in PCF than BSF) and used for FA remodelling in the two main life cycle stages of *T. brucei* [26–28,31]. When altering the level of expression of Tb-Δ6, particularly in the presence of reduced levels of FAs in the media, phenotypical responses, such as modulation of the growth rate and morphological changes, were revealed (Fig 8B). Here we demonstrated that trypanosomes finely tune the metabolism of FAs to achieve an appropriate redistribution of SAFAs and UFAs in the membranes, especially upon genetic manipulation of Tb-Δ6 and availability of extracellular FA sources [62,63]. In fact, we detected significant differences in the level of 20C and 22C PUFAs in our mutants, which ultimately allowed the true activity of Tb-Δ6 to be unveiled. This data shows that the desaturase has a key role in the synthesis of essential ω-3 VLC-PUFA, namely 22:5 and 22:6, by working as Δ4-ω-end acyl-CoA desaturase, and in concerted action with other endogenous desaturases and elongases (such as ELO4 present in the ER) (Figs 4A and 8B) [16,32]. Accordingly, the expected reduction of 22:4 and 20:4 substrates, and of 18:2, observed in the lipidomic data, was also confirmed in the metabolomics dataset (Fig 8A). Furthermore, the HA-tagged Tb-Δ6 was detected to be partially localised/associated with the mitochondria (Fig 2C and 2D). The patchy signal obtained may suggest that the desaturase is localised in the mitochondrial contact sites with the ER [64], which would explain how the desaturated products of Tb-Δ6 from the mitochondria comes in rapid contact with ELO 4 in the ER to be efficiently elongated to the very final product (Fig 4A) [17]. In line with the elucidated activity of Tb-Δ6, OE-D6 BSF showed downregulation of ACS3, which normally uses mid-chain FAs as preferential substrates (Fig 7). This is in fact understandable when considering the large abundance of PUFAs in OE-D6 cells, and a possible need for compensatory mechanism towards shorter chain FAs [65]. Alongside that, we observed down regulation of the vacuolar sorting protein 39 domain 1/2, which is part of the late endosomal/lysosomal homo-typic fusion and vacuole protein sorting (HOPS) complex (Fig 7), which may suggest an attempt of the cells to regulate the endocytic pathway, including the FAs uptake in OE-D6 cells [54].

These changes in the PUFA content in turns affects the lipid metabolism and therefore membrane composition, such that the cells respond to the changes in the cellular and external environments by maintaining membrane homeostasis (Fig 8B) [60,66]. This is key when considering that *T. brucei* parasites encounter both fat-rich and fat-poor environments, between the mammalian host and  insect vector [11]. Indeed, the regulation of the activity of Tb-Δ6 in the PUFA synthesis highlights the direct crosstalk with the lipid metabolism in *T. brucei* PCF and more evidently in BSF (Fig 8B) [38]. In fact, we demonstrated that the BSF trypanosomes can initiate a remodelling of their FA, phospholipid and sphingolipid pools, as a consequence of the change in the levels of 20C and 22C PUFAs, ultimately contributing to morphological changes in the cells. In fact, we were able to observe an increase in glycerophosphorylethanolamine (Fig 8A), which implies that phosphatidylethanolamine (PE) lipids are being catabolised, possibly in response to the high cellular level of 22:6 and 22:5 PUFAs, and/or lack of cell division, observed temporarily in our OE-D6 BSF, as copious amounts of PE are required for cell division [14,38]. Surprisingly, when the expression level of Tb-Δ6 was increased, therefore the amount of 22:5 and 22:6 was higher than normal, and upon scarce FA sources in the media, BSF trypanosomes started producing IPC (Fig 8B). This response is likely to control membrane fluidity caused by the consequential reduction of PUFAs and the redistribution of ceramide towards the synthesis of IPC (Fig 8B), similar to that previously observed in stumpy [14,28,38]. This evidently suggests an increase in the cell membrane rigidity, which may be key in the transition from slender to stumpy form, so that the cells can pre-adapt, allowing better survival rates in the harsher and more acidic environment of the Tsetse fly’s salivary glands and midgut [14,28,38]. These findings are very intriguing on several levels, considering that IPC is normally only produced in PCF and the pre-adaptive stumpy stage of BSF (long slender), in which both life cycle stages express Tb-Δ6 at a much higher level than in BSF [26,28,29]. Thus, by just over-expressing Tb-Δ6 in BSF to recapitulate the higher levels observed in stumpy form, we have observed a cascade of other metabolic and proteomic changes. These include the presence and activity of the mitochondrial IPC synthase (SLS1) (Tb927.9.9410), normally upregulated in the stumpy form (Fig 8B) [28,29]. When the expression of Tb-Δ6 is turned down in PCF, this results in small downregulation of SLS1 and a modest reduction in IPC synthesis, which causes the PCF parasites to slow their growth. When they are supplemented with higher serum levels, they return to normal growth, implying they scavenge the FAs from the higher serum level to achieve this.

Additionally, the clemastine fumarate inhibition experiment in the over-expressing Tb-Δ6 BSF *T. brucei* is clearly not selective for just IPC synthase, but also may hit SM synthesis and possibly ethanolaminephosphate-ceramide synthesis.

In keeping with recent studies by Machado *et al.* [67], our findings show that some environmental PUFAs can have a dramatic detrimental effect on the parasite cell growth, like shown for WT and KD-D6 PCF under quite low concentrations of DHA supplementation, unlike the addition of AA (20:4), which has no detrimental effect in either BSF or PCF, as observed in ongoing studies in our lab. Despite that, WT and KD-D6 PCF cells showed the typical full range of cellular FA species, where an increase of C18:0/C18:1 and C18:2 ratio was observed to ensure once again membrane fluidity homeostasis and cell survival. The effect of this lipid remodelling and downstream events on the cell morphology was very evident in the OE-D6 BSF cells grown in low-fat media. OE-D6 BSF cells shifted from an elongated slender to a ‘plumpy’ morphology, which resembles a “half-way point” towards the stumpy morphology, while also expressing an intermediate level of PAD1 at the timepoint where most of the cells were found in the non-dividing stage of their cell cycle (1K1N), before restoring their growth (Figs 6 and 8B) [41,44]. Interestingly, OE-D6 cells revealed a very distinct tubular punctuated pattern for PAD1, compared to a more diffuse signal typical of the stumpy form. This may suggest that when the cells are at the “half-way point” towards the stumpy morphology, the expression of PAD1 may be initially highly localised at the parasite’s surface (Fig 6A). Encouragingly, we were also able to show that several other proteins, which are normally overexpressed in the stumpy form of *T. brucei*, were also upregulated in OE-D6 BSF (Fig 7). Particularly a putative cysteine peptidase (Tb927.11.14210), which have been identified as taking part in the quorum sensing signal production, was upregulated, as well as the level of some oligopeptides, present in OE-D6 BSF cellular metabolites [7,59]. This shows that, similarly to the stumpy form, the peptidases are highly active in OE-D6 cells [59,68–70]. Despite that, we were not able to detect upregulation of pyroglutamyl (Tb927.4.2670) and prolyl (Tb927.10.8020) peptidases, and of the Golgi pH regulator protein (TGPR89, Tb927.8.1530), which all have major roles in the production and reception of SIF oligopeptides [7,59]. Furthermore, the upregulation of ATP synthase subunit Ɛ (Tb927.11.600), normally highly expressed in PCF, and POMP12 (Tb927.11.2750), as well as a reduction in level of succinate and adenyl-succinate, suggesting mitochondrial reprogramming, which are key aspects in stumpy differentiation [10,46–48,71]. Particularly, POMP12 is important for protein import into the mitochondria, and mitochondria function and morphology maintenance throughout cell division [46]. This plays a vital role in stumpy differentiation allowing reprogramming of the mitochondria to allow protein import of newly synthesised proteins involved in carbon source usage and alteration in the membrane potential [47].We also observed the upregulation of the glycosomal protein dihydroxyacetone phosphate acyltransferase (Tb927.4.3160). This protein is important for the biosynthesis of ether lipid precursors and has a key role in the resulting cellular morphology, signal transduction and cell cycle progression, which are fundamental processes in stumpy differentiation [49]. In the same vein, the upregulation of a PPQ protein (Tb927.11.10480), which plays roles in signal transduction, transcription regulation and cell cycle control was detected [50]. Interestingly, OE-D6 BSF revealed upregulation of an EF-hand domain pair (Tb927.11.8390), which normally bind to Ca^2+^, and are associated with proteins such as cytokinesis initiation factor 2, which localises to the flagellum attachment zone (FAZ) tip only during early cell cycle stages, i.e., 1N1K [53]. These domains are also identified in the cAMP Response Proteins (CARPs) to have a regulatory role to the cAMP response, which may be linked to the cAMP pathway in the cell cycle arrest in the stumpy form of *T. brucei* [51–53]. In support of that, the reduction of nicotinate and orotate, and of adenine and adenyl-succinate, as well as decrease in N, N, N-trimethyl-L-lysine were observed; an indication of reduced DNA/RNA formations as the cells exhibit slower division between 24h and 36h time point [55–57], and of a possible increase in histones methylation process (Fig 8A) [58]. Unsurprisingly, our data does not match perfectly the level of differential protein expression observed in previous differentiation studies, partly due to the use of monomorphic parasites (*T. brucei* strain 427) in our studies. Monomorphic parasites are unable to fully differentiate *in vitro*, unless induced under specific culture conditions (*i.e.,* glucose removal, *cis*-aconitate, temperature change etc.) [72,73]. Furthermore, monomorphic parasites are cultured under significantly different conditions compared to the pleomorphic *T. brucei* used in the previous differentiation studies [26–28,31]. Furthermore, as far as the authors are aware there is no published metabolite dataset of stumpy BSF cells to allow a comparison with our observed metabolite changes. Collectively, these data add an extra layer of information that suggests that the sole overexpression of Tb-Δ6 in BSF, and the consequential production of IPC, are linked through a cascade of metabolic events, observed in several metabolic and proteomic changes, that might contribute in part to the transition from the slender BSF to the pre-adaptive stumpy form and thus to the PCF (Fig 8B) [28].

At this point, we can highlight that, many of the proteomic, biochemical and morphological changes observed in this work, by over-expressing Tb-Δ6 in BSF would be downstream of the SIF and quorum sensing as part of the stumpy differentiation process [7]. It is important to add that these over-expressing Tb-Δ6 cells only stall their growth for a short period of time, before continuing to divide, so despite producing “stumpy” markers, these are not caused by the cell stalling [5].

On-going work in the lab on metabolomics and proteomics of pleomorphic *T. brucei* Antat1.1 is attempting to establish to what extent Tb-Δ6 expression levels are contributing to the shift from slender to stumpy and finally PCF forms, *via* morphological change processes. It will also be fundamental to investigate the role of proteins involved in cytoskeletal remodelling, we know to be differentially regulated in stumpy forms [28]. In fact, together with enzymes involved in lipid metabolism and membrane remodelling, they are key contributors to the morphological adaptation of the parasite transmissible forms [6,74,75]. There are many exciting questions still to be answered, but so far we clearly showed that FAs, lipids and their metabolic enzymes are key to trypanosomes’ cell growth and to their efficient adaptation to their hosts’ environments [10,60]. We demonstrated that trypanosomes are able to take advantage of those finely tuneable mechanisms to control their cell growth, based upon the level of extracellular fat sources available and the homeostasis of their membranes to meet their cellular requirements based upon the environment [10,60]. These are all mechanisms that actively contribute to the morphological and metabolic needs of the highly adaptable parasite *T. brucei* during different phases of its complex life cycle.^,^

## Materials and methods

Unless otherwise stated, all reagents and materials were purchased from Sigma, Promega, New England Bio Labs (NEB) or VWR. SPLASH LIPIDOMIX Mass Spec Standards was purchased from Avanti Polar Lipids, Gibco Iscove’s Modified Dulbecco’s Medium (IMDM) and Gibco Foetal Bovine Serum (FBS) were purchased from Thermo Fisher. SDM-79 powder was obtained from Life Technologies.

### Cell culture

*T. brucei* PCF (Lister 427) parasites were grown at pH 7.4, 28°C and under 5% CO_2_ in SDM-79 media supplemented with either 10% or 1.25% (v/v) of heat inactivated FBS, 11.5 μM haemin and 24 mM NaHCO_3_ [76]. PCF were kept under drug selection pressure from G418 (neomycin) (15 μg/mL) and hygromycin (50 μg/mL) [36]. *T. brucei* PCF RNAi cell lines were grown in the presence of 1.25 μg/mL phleomycin or *T. brucei* PCF overexpression cell line in the presence of 5 μg/mL blasticidin. *T. brucei* BSF (Lister 427) parasites were grown at pH 7.4, 37°C and under 5% CO_2_ [3]. in HMI-11 media containing 80% (v/v) IMDM, 1.25 mM pyruvic acid, 161 µM thymidine, 50 μM bathcuperoinedisulfonic acid, 1 mM hypoxanthine, 1.5 mM L-cysteine, 58 μM 1-thioglycerol and supplemented with either 10% or 5% (v/v) heat inactivated FBS. BSF were kept under drug pressure from G418 (2.5 μg/mL) [36]. *T. brucei* BSF RNAi cell line were grown in the presence of 2.5 μg/mL phleomycin and *T. brucei* BSF overexpression cell line in the presence of 5 μg/mL blasticidin. Addition of tetracycline to the media, when required, was at a final concentration of 1 μg/mL. Pleomorphic *T. brucei* BSF (strain EATRO 1125 AnTat1.1, kindly donated by Prof Keith Matthew) parasites were grown at pH 7.4, 37°C and under 5% CO_2_ in HMI-11 media containing 80% (v/v) IMDM, 1.25 mM pyruvic acid, 161 µM thymidine, 50 μM bathcuperoinedisulfonic acid, 1 mM hypoxanthine, 1.5 mM L-cysteine, 58 μM 1-thioglycerol and supplemented with 20% (v/v) heat inactivated FBS [76]. BSF were kept under drug pressure from G418 (2.5 μg/mL) and hygromycin (50 μg/mL) [36]. Pleomorphic *T. brucei* BSF were differentiated into stumpy by supplementing the culture media with 15% Brain Heart Infusion (BHI) for 48h induction as described in Rojas *et al*. [7] Cell counting was performed using a Model-TT CASY Cell Counter and Analyser system. Cells were maintained at mid-log phase and passaged every 3 days (PCF) or every 2 days (BSF strain 427 and EATRO 1125 AnTat1.1)*.*

### Chemical supplementation of *T. brucei* PCF and BSF culture media with docosahexaenoic acid

The media SDM-79 supplemented with 1.25% FBS and HMI-11 supplemented with 5% FBS in the presence of the appropriate selection drugs as described above and tetracycline at a final concentration of 1 μg/mL, were supplemented with DHA (22:6) to a final concentration in the flask of 10 μM (stock solution 1M in DMSO).

### Growth curves for *T. brucei* PCF and BSF

PCF trypanosomes were grown to mid-log phase in SDM-79 supplemented with 10% or 1.25% FBS and distributed into non-vented flasks at an equal density of 5 x 10^5^ cell/mL in the presence or absence of the appropriate concentration of selection drugs and tetracycline according to the cell line. The cells were grown for 72 h and counted every 24 h using a Model-TT CASY Cell Counter and Analyser system. BSF trypanosomes were grown to mid-log phase in HMI-11 supplemented with 10% or 5% FBS and distributed into vented flasks at an equal density of 1.5 x 10^5^ cell/mL in the presence or absence of appropriate concentration of selection drugs and tetracycline according to the cell line. The cells were grown for 48 h and counted every 12 h using a Model-TT CASY Cell Counter and Analyser system.

### Generation of Tb-Δ6 RNAi construct using p2T7-177 vector

The ORF of *Tb-*Δ*6* (Tb11.v5.0580.1) gene was amplified from *T. brucei brucei* lister 427 genomic DNA using KOD Hot Start DNA Polymerase (Novagen), and the following forward and reverse primers 5’-TTT TTG GAT CCA TGA GTT CGG TAA AGA GTA AAG G-3’and 5’-TTT TTC TCG AGC AAC CGT TTG TCT TCT ATG C-3’ containing *BamH*I and *Xho*I restriction sites (underlined regions). The purified product was digested with *BamH*I and *Xho*I and ligated into appropriately digested p2T7-177, containing the phleomycin (Phleo) resistance cassette. The plasmid obtained was defined as p2T7-177-Tb-Δ6-Phleo and the presence of the insert was confirmed by PCR colony screening using the following sets of forward and reverse primers 5’-ATAGAGATCTAGCCGCGGTGG–3’ and 5’–CATGATGAATGATCCAACTGATGC–3’ (expected product size 630 bp), and 5’-CCCTATCAGTGATAGAGATCTCC-3’ and 5’–CAAACGAACCTCATCATGACTTGG–3’ (expected product size 702 bp), using GoTaq DNA polymerase (Promega), and sequencing at Eurofins Genomic.

### Generation of Tb-Δ6 overexpression construct using pLew-100-C-term-HA vector

The ORF of *Tb-*Δ*6* (Tb11.v5.0580.1) gene was amplified from *T. brucei brucei* lister 427 genomic DNA using KOD Hot Start DNA Polymerase, using the following forward and reverse primers 5’-ATA GTA CAA GCT TAT GAG TTC GGT AAA GAG TAA AGG -3’ and 5’- GAT AGC TTA ATT AAC AAC CGT TTG TCT TCT ATG C-3’ containing *Hind*III and *Pac*I restriction sites (underlined regions). The purified product was digested with *Hind*III and *Pac*I and ligated into appropriately digested pLew-100-C-term-HA, containing the blasticidin (BSD) resistance cassette. The plasmid obtained was defined as pLew-100-Tb-Δ6-C-term-HA-BSD and the presence of the insert was confirmed by PCR colony screening (expected size product 1499 bp), with the following forward 5’-CTC GTC CCG GGC TGC ACG CGC CTTCCG-3’ and reverse 5’-CCT GCA GGC GCA CCT CCC TGC TGT G-3’ primers, using GoTaq DNA polymerase (Promega), and sequencing at Eurofins Genomic.

### Transfection of *T. brucei* PCF and BSF

1 x 10^7^ PCF and BSF mid-log phase cells were harvested and transfected using Lonza T-cell nucleofector kit and Amaxa-electroporator using program X-014 (PCF) and X-001 (BSF) as reported in Burkard *et al*. [77] The transfected trypanosomes were transferred into a 24 well/plate and incubated at 28°C (PCF) or 37°C (BSF) under 5% CO_2_ for 24 h. At this point, 2.5 μg/mL (PCF) or 5 μg/mL (BSF) of phleomycin, and 10 μg/mL (PCF and BSF) of blasticidin were added. After several days, the cells that showed the highest growth rate were selected and OE-Δ6 BSF and PCF, KD-Δ6 BSF and PCF and OK-Δ6 PCF genotypes were verified by PCR analysis of genomic DNA isolated from parental and RNAi (primers used 5’-TTT TTG GAT CCA TGA GTT CGG TAA AGA GTA AAG G-3’ and 5’-ATG GCC AAG TTG ACC AGT GC-3’), overexpression (primers used 5’-GTC TCA AGA AGA ATC CAC CCT C-3’and 5’- GAT AGC TTA ATT AAC AAC CGT TTG TCT TCT ATG C-3’) and add-back (both pairs of primers listed above) cell lines.

### Total RNA extraction and qRT-PCR

Total RNA was isolated from 1x10^8^ mid-log BSF and PCF *T. brucei* cells, cultured in the presence or absence of Tet for 48h, using the RNeasy mini kit (Qiagen). RNA was treated with Precision DNase Kits (Primer Design) to remove any excess of gDNA carried over from the extraction of total RNA. A *Tb-*Δ*6*-specific cDNA was generated and amplified using a mini-exon-specific forward primer 5’-ACG CTA TTA TTA GAA CAG TTT CTG TAC TAT ATT GAC TTT C AT GAG TTC-3’, in combination with an ORF-specific reverse primer 5’-TGT AGT GTC GGG TTC ATC GG-3’, specific for the sequence of *Tb-*Δ6 [78,79]. The *T. brucei* actin gene was used as endogenous control (*Tb-Act*) using a mini-exon-specific forward primer 5’-GCG GAG ACT TCG AAC GCT ATT ATT AGA ACA GTT TCT GTA -3’ and ORF-specific reverse primer 5’-CTT CAT GAG ATA TTC CGT CAG GTC-3’, specific for the sequence of *Tb*Actin [78,79]. cDNA synthesis and qRT-PCR were performed in a one-pot reaction using Luna Universal One-Step RT-qPCR Kit (NEB). A QuantStudio Real-Time PCR System (ThermoFisher) thermocycler was used following standard cycling conditions. The data were analysed by the 2^−ΔΔCT^ method normalising with *Tb*Actin.

### Lipid extraction

2 x 10^7^ cells of mid log phase *T. brucei* PCF and BSF, cultured for 48 h in the presence or absence of Tet, were collected by centrifugation at 800 x *g* for 10 min at room temperature. The cell pellet was washed with either phosphate-buffered saline (PBS) (for PCF) or trypanosome dilution buffer (TDB) (5 mM KCl, 80 mM NaCl, 1 mM MgSO_4_, 20 mM Na_2_HPO_4_, 2 mM NaH_2_PO_4_, and 20 mM glucose at pH 7.7) (for BSF), after which they were re-suspended in 100 μL of PBS and transferred to a glass vial. 375 μL 2:1 (v/v) of MeOH:CHCl_3_ solution was added for biphasic separation based on the following method described by Bligh-Dyer [80]. The samples were vigorously agitated at 4°C overnight. The internal standards Avanti-SPLASH were prepared by transferring the 1mL MeOH solution in the vial and suspending in 1 mL 1:1 (v/v) of MeOH:CHCl_3_. 20 μL of this were added to the samples at the point of extraction. Samples were made biphasic by the addition of 125 μL CHCl_3_ and 125 μL distilled sterile water, followed by vigorous agitation after each addition. The samples were centrifuged at 1000 x *g* for 5 min to allow the aqueous and the organic layers to separate. The organic phase was transferred into a new glass vial and dried under nitrogen gas stream to obtain the total lipid extract ready to use and stored at 4°C until further analysis.

### Electrospray ionization mass spectrometry and tandem mass spectrometry (ESI-MS/MS)

Lipid samples were analysed by electrospray ionization tandem MS (ESI-MS/MS) with an AB-Sciex Qtrap 4000 triple quadrupole mass spectrometer fitted with an Advion TriVersa NAnoMAte nanoelectrospray. Survey scans in negative mode were used for the detection of PE, EPC, PI, IPC, PS, PG and PA (cone voltage = 1.25 kV). Positive ion mode survey scans were used to detect PC and SM (cone voltage = 1.25 kV). In negative ion mode, a precursor of m/z 196 scans were used to detect PE and EPC species, a precursor of m/z 241 scans for IPC and PI species, a precursor of m/z 153 scans for PG and PA (collision energy, CE 60 eV). In positive ion mode, a precursor of m/z 184 scans were used to detect PC and SM species (CE 60 eV). Neutral loss of a precursor of m/z 87 scans were used for PS (CE 60 eV). Spectra were acquired over or within a range of 120-1000 m/z, and each spectrum represents a minimum of 30 consecutive scans with nitrogen collision gas. Samples were run using a 1:1 solvent mixture of 2:1 (v/v) MeOH:CHCl_3_ and 6:7:2 (v/v) acetonitrile:isopropanol:dH_2_O. Individual lipid species were annotated according to their acyl composition determined by daughter ion scans produced and compared to previous lipid identification by Richmond *et al.* [14], and to theoretical values contained in the Lipid Metabolites and Pathways Strategy consortium database (LIPID MAPS, http://www.lipidmaps.org/).

### Fatty acids transmethylation and gas chromatography – Mass spectroscopy (GC-MS) samples preparation

Transmethylation of the fatty acids was performed on the dry total lipid extracts [81]. The reaction (total volume 1 mL) was conducted in a glass vial. 100 μL of toluene were added, followed by 750 μL of MeOH and 150 μL of 8% solution of HCl in MeOH:H_2_O (85:15 (v/v)) to allow the formation of fatty acid methyl esters (FAMEs). The reaction was left to react at 45°C overnight. Upon drying under a nitrogen gas stream, the FAMEs were extracted with a 1:1 (v/v) hexane:H_2_O solution. The FAME extracts in hexane were dried under a nitrogen gas stream and dissolved in dichloromethane, typically 15 μL, of which 1-3 μL were analysed by GC-MS on an Agilent Technologies GC-6890N gas chromatograph coupled to an MS detector‐5973. Separation by GC was performed using a Phenomenex ZB-5 column (30 m x 25 mm x 25 mm), with a temperature program of 70°C for 10 min, followed by a gradient to 220°C, at 5°C/min, which was maintained at 220°C for a further 15 min. Mass spectra were acquired from 50-500 amu [82]. FAME species were assigned by comparison of the retention time, fragmentation pattern, use of FAME standards, and the online FAMEs mass spectrometry database (http://www.lipidhome.co.uk/ms/methesters/me-arch/index.htm).

### Western blot probed with an anti-HA antibody and samples preparation

1 x 10^7^ mid-log-phase PCF and BSF parasites, cultured for 48 h in the presence or absence of Tet, were harvested by centrifugation at 800 x g for 10 min. The cells were washed in PBS twice and re-suspended in 50 μL of 2 x SDS sample buffer and incubated at 95°C for 10 min. SDS-PAGE was performed on 12% acrylamide gel. The protein gel was then transferred onto a membrane (GE Healthcare Life Science UK) and the membrane blocked in 5% semi skimmed milk powder in PBS. The membrane was incubated for 1 h at room temperature with Odyssey blocking buffer and 0.2% PBS-T (PBS containing 0.2% (v/v) Tween-20) with rat anti-HA clone 3F primary antibody (Clontech) at 1:1,000 dilution. The membrane was washed for 3 x 10 min with 0.2% PBS-T buffer. Subsequently, the membrane was incubated for 1 h at room temperature with secondary goat anti-rat conjugated with DyLight 800 (Licor IRDye 800) antibody at 1:10,000 dilution. The membrane was washed as above, before the fluorescence was detected with an Odyssey western blot detection system (Licor Odyssey).

### Immunofluorescence microscopy for *T. brucei* PCF and BSF

1 x 10^6^ mid-log phase PCF, BSF and stumpy (only used for PAD-1 detection) trypanosomes, cultured for 48 h in the presence or absence of Tet, where required, were harvested at 800 x g for 10 min and washed with PBS. (i) For mitochondrial visualisation, cells were incubated for 30 min at 28°C or 37°C, for PCF and BSF respectively, with 100 nM of MitoTracker red CMZRos in 100 μL of fresh SDM-79 or HMI-11. The cells were spun at 3,000 rpm for 2 min and re-suspended in 100 μL of fresh media and incubated at 28°C or 37°C for 15 min, to allow the MitoTracker to enter the mitochondria. The cells were spun again as above and washed with PBS. The cells were resuspended in 100 μL of 4% paraformaldehyde (PFA) in PBS and incubated at room temperature for 15 min. After this time, the cells were spun at 3000 rpm for 2 min, the supernatant removed, and the cells washed with PBS and re-suspended in 100 μL fresh PBS. 50 μL of cells were added to Polysine slides (VWR 631-0107) and allowed to adhere overnight at room temperature in a sealed container on a damp tissue to prevent evaporation. The cells were re-hydrated with 100 μL of dH_2_O for 5 min and washed with 100 μL of PBS for 2 x 5 min using a pipette. The cells on the slide were incubated with 100 mM glycine in PBS for 5 min. The cells were washed for 3 x 5 min with 100 μL of PBS. At this point 50 μL of 0.1% TritonX-100 solution in PBS were added to permeabilise the cells for 10 min. The cells were washed for 3 x 5 min with 100 μL of PBS and blocked for 20 min with 1% BSA in PBS. The cells were washed for 1 x 5 min with 100 μL of PBS. (ii) For HA-tag localisation, the primary rat anti-HA antibody was diluted to 1:500 in 1% BSA-PBS, and the cells were incubated with this solution at room temperature for 2 h. The cells were washed with 100 μL of 1% BSA-PBS for 3 x 5 min. The secondary chicken anti-rat Alexa Fluor 594 antibody (Thermo Fisher) was diluted to 1:1,000 in 1% BSA-PBS, and the cells were incubated with this solution at room temperature for 2 h. The slides were washed for 3 x 5 min with PBS in a Coplin jar. The slides were thoroughly dried with the use of a pipette. (iii) For PAD1 visualisation, the cells were blocked for 45 min with 2% BSA in PBS in a humidity chamber at 37°C. The cells were washed for 1 x 5 min with 100 μL of PBS. Primary rabbit anti-α-PAD1 antibody (kindly given by Prof Keith Matthews, The University of Edinburgh) was diluted to 1:1,000 in 2% BSA-PBS, and the cells were incubated with this solution at 37°C in a humidity chamber for 45 min. The cells were washed with 100 μL of 2% BSA-PBS for 3 x 5 min. The secondary chicken anti-rabbit FITC antibody (Thermo Fisher) was diluted to 1:250 in 2% BSA-PBS, and the cells were incubated with this solution at 37°C in a humidity chamber for 45 min. The slides were washed for 3 x 5 min with PBS in a Coplin jar. (iv)To stain the DNA, the cells were incubated with 50 μL DAPI (4,6-diamidino-2-phenylindole) (2 μg/mL, in PBS) for 5 min in the dark. The slides were washed for 3 x 5 min with PBS. A drop of antifade agent was added to each sample on the slide, covered with a coverslip and let dry at room temperature overnight, before sealing the coverslips with nail polish. Images were acquired with a fluorescence confocal microscope (DeltaVision Imaging System), connected to a digital camera system, and processed by softWoRx Explorer 1.3 and ImageJ image analysis software. Quantitative analysis was carried out using Volocity 6.0 software.

### Live/dead dose-response cytotoxicity assay in *T. brucei* BSF for clemastine fumarate

The Alamar Blue cell viability assay was performed by incubating 1 × 10^3^ cells/well (final volume 200 μL) mid-log-phase *T. brucei* BSF cells with the clemastine fumarate (DMSO stock of 50 mM) and 2-fold serially diluted in HMI-11 in a concentration range from 200-0.20 μM) at 37˚C with 5% v/v CO_2_ for 72 h. After this, 10 μL Alamar Blue (1.1 mg/mL resazurin sodium salt in PBS) was added to all wells and the cells incubated for a further 7 h before recording fluorescence [83]. Fluorescence was recorded using a FLx 800 plate reader (BioTek) with excitation wavelength 530–535 nm and emission wavelength at 590–610 nm and data were processed using Gen5 Reader Control 2.0 Software (BioTek). EC_50_ values were determined using a 4-parameter non-linear logistic regression equation using GraFit 5.0 (Erithacus Software). SD values were calculated based on curve fitting to 4 biological replicates performed in parallel.

### Proteomics sample preparation

2 x 10^7^ mid-log-phase BSF parasites, cultured for 48 h in the presence or absence of Tet, were harvested by centrifugation at 800 x g for 10 min at 4°C. The cells were transferred to pre-cooled Eppendorf tubes, then pelleted by centrifugation (3000 rpm, 3  min, 4°C) and the supernatant removed. The cells were washed with 500 µL of TDB twice (3000 rpm, 3  min, 4°C) and the supernatant removed. Cell pellets were lysed with aqueous urea (aq. 5 M, 20 μL) and stored at -80°C until analysis. The protein content of each sample was determined by Bicinchoninic acid (BCA) assay as per manufacturer instructions. The samples were then snap frozen in liquid N_2_ and stored at -80˚C until further analysis.

### Mass spectrometry analysis of cellular protein samples

Sequential window acquisition of all theoretical fragment ion spectra mass spectrometry (SWATH MS) analysis was performed on the cellular protein samples. The samples were treated *via* disulfide reduction (dithiothreitol), cystine alkylation (iodoacetamide) and digestion by trypsin protease, and peptide fragments were eluted through Easy Spray Pepmap RSLC 75 μm × 50 cm, C18, 2μm, 100A analytical column and a Trap cartridge Pepmap100 100 μm × 2 cm, C18, 5μm, 100A. Three different buffers were using for loading and elution: loading buffer (100% water, 0.05% TFA), mobile phase A (100% water, 0.1% formic acid) and mobile phase B (80% acetonitrile, 20% water, 0.1% formic acid). The samples were analysed using Orbitrap Fusion Lumos Tribrid (Thermofisher) with Easyspray source and a NanoLC system (Ultimate 3000 Nano RSLC system). Results were analysed by Mascot and Skyline software and compared to the *T. brucei* 927 database. Statistical analysis was performed using Perseus software.

### Metabolomics sample preparation

1×10^8^ mid-log-phase BSF parasites, cultured for 48 h in the presence or absence of Tet, were harvested, then their metabolism was quenched by rapid cooling to 4°C using a CO_2_/ethanol bath. Cells were stored on ice for the remainder of the procedure. Cells were pelleted by centrifugation at 800 x g for 10  min at 4°C. Cells were resuspended in 500 μL PBS and transferred to pre-cooled Eppendorf tubes, then pelleted by centrifugation at 3000 rpm for 5 min at 4°C and the supernatant removed. Cells were then washed with 500 μL PBS, pelleted by centrifugation at 3000 rpm for 5 min at 4°C and the supernatant removed. The cell pellet samples were resuspended in 200 μL of a cooled mixture of chloroform:methanol:water (1:3:1) followed by shaking for 1 h at 4°C. Insoluble debris was then removed from the extraction mixture by centrifugation at 15000 g for 15 min at 4°C and the supernatant collected. A quality control sample was created to ensure accurate metabolite analysis by combining 20 μL of each sample within the study. Samples were stored under N_2_ and frozen at -80°C until analysis.

### Mass spectrometry analysis of cellular metabolite samples

Metabolomics samples were analysed *via* liquid chromatography-mass spectrometry (LC-MS) based on methods previously described [84]. Hydrophilic interaction liquid chromatography (HILIC) was performed using a Dionex UltiMate 3000 RSLC system (Thermo Fisher Scientific, Hemel Hempstead, UK) using a ZIC-pHILIC column (150 mm x 4.6 mm, 5 μm column, Merck Sequant). The column was maintained at 25°C and samples were eluted with a linear gradient using the solvent system A) 20 mM (NH4)2CO3 in H2O and B) C2H3N over 26 minutes (flow rate 0.3 ml/min). Samples were injected (10 μl) and were maintained at 5°C prior to injection. MS analysis was performed using a Thermo Orbitrap QExactive (Thermo Fisher Scientific) operated in polarity switching mode (Resolution = 70000; AGC = 1e6; m/z = 70–1050; Sheath gas = 40, Auxiliary gas = 5, Sweep gas = 1; Probe temperature = 150°C, Capillary temperature = 320°C). For positive mode ionisation: source voltage +3.8 kV, S-Lens RF Level 30.00, S-Lens Voltage -25.00 (V), Skimmer Voltage -15.00 (V), Inject Flatopole Offset -8.00 (V), Bent Flatapole DC -6.00 (V). For negative mode ionisation: source voltage -3.8 kV. Pooled samples were employed for quality control purposes to assess reproducibility of the instrument, being analysed at 5-sample intervals throughout the run. Data analysis was performed using the Polyomics Integrated Metabolomics Pipeline (PiMP) [85]. Statistical analysis was performed using Perseus software.

### Statistical analysis

For all growth curves, FAMEs, lipids and qRT-PCR experiments values are the mean of three independent biological replicates (n = 3). Error bars represent the standard deviation of each mean calculated by GraphPad PRISM 6.0. Statistical analysis, where required, was also performed by GraphPad PRISM 6.0 using One-way or 2-ways ANOVA multiple comparisons or based on a Tukey or Dunnet t-test or unpaired t-test with a 95% confidence interval. Volcano plot data were obtained through a Two-ways student T-test analysis using Perseus software and GraphPad PRISM 6.0. Microscopy fluorescence quantification analysis was carried out using Volocity 6.0 software, and statistical analysis was carried out using GraphPad PRISM 6.0. as above.

## Supporting information

S1 Appendix FAME FBS-FAs. (CSV)

S2 Appendix *T. brucei* BSF-FAs. (XLSX)

S3 Appendix *T. brucei* PCF-FAs. (XLSX)

S4 Appendix *T. brucei* BSF and PCF lipid content. (XLSX)

S5 Appendix Tb-D6-qRT-PCR. (XLSX)

S6 Appendix Proteomics data. (CSV)

S7 Appendix Metabolomics data. (XLSX)

S8 Appendix PAD1 quantification data. (XLSX)

S9 Appendix Nuclei and kinetoplasts count. (XLSX)

S1 Table Fatty acid content of foetal bovine serum (FBS) used to supplement SDM-79 and HMI-11 media. The table shows a summary of the relative abundance and the retention times of the FAMEs or FAs after GC-MS analysis of samples obtained from FBS. Values are the mean of three independent biological replicates (n = 3). SD is standard deviation of each mean (±) (S1 Appendix).
(DOCX)

S1 Fig GC-MS analysis of the FAMEs in *T. brucei* PCF and BSF. GC-MS chromatogram analysis of *T. brucei* PCF WT cultured for 48 h in SDM-79 with 1.25% FBS (A) and *T. brucei* BSF WT cultured in HMI-11 with 5% FBS (B). Peaks eluted at different retention times (X axis) and with different abundance (Y axis), are assigned to specific FAs. The C20 (green (A) and light blue (B) brackets and inserts) and C22 PUFAs (orange (A) and purple (B) bracket and insert) are expanded. The red lines indicate the major species of FAs. Note: ‘= first eluted isomer; “= second eluted isomer; first and second eluted isomers are FAs with same number of C on the alkyl chain, and the same number of double bonds in different position along the alkyl chain The chromatograms are representative of experiment results conducted in three independent biological replicates (n = 3).
(DOCX)

S2 Fig GC-MS analysis of the fatty acids in Tb-Δ6 genetically manipulated *T. brucei* PCF in high-fat media. A) The bar chart shows the different PUFAs (X axis, the order follows increasing retention time) and the relative abundance (Y axis) found in Tb-Δ6 genetically modified *T. brucei* PCF (KD-D6, OE-D6, OK-D6) and WT control, when the cells are cultured for 48 h in SDM-79 supplemented with 10% FBS, in the presence of tetracycline as shown in the legend. B) The bar chart is an expansion of the region including 20:4 PUFAs from A. C) The bar chart is an expansion of the region including 22:4, 22:5, 22:6 PUFAs from A. Values are the mean of three independent biological replicates (n = 3). Error bars represent the standard deviation of each mean. All FAs were identified using GC-MS based upon retention time, fragmentation, and comparison with standards. Statistical analysis was performed by GraphPad PRISM 6.0 using 2-way ANOVA multiple comparisons based on a Tukey t-test with a 95% confidence interval, where **** is p ≤ 0.0001, *** is p ≤ 0.001, ** is p ≤ 0.01 and * is p ≤ 0.05. Note: ‘ = first eluted isomer; “ = second eluted isomer (S3 Appendix).
(DOCX)

S3 Fig GC-MS analysis of the fatty acids in Tb-Δ6 genetically manipulated *T. brucei* PCF in low-fat media. A) The bar chart shows the different PUFAs (X axis, the order follows increasing retention time) and the relative abundance (Y axis) found in Tb-Δ6 genetically modified *T. brucei* PCF (KD-D6, OE-D6, OK-D6) and WT control, when the cells are cultured for 48 h in SDM-79 supplemented with 1.25% FBS, in the presence of tetracycline as shown in the legend. B) The bar chart is an expansion of the region including 20:4 PUFAs from A. C) The bar chart is an expansion of the region including 22:4, 22:5, 22:6 PUFAs from A. Values are the mean of three independent biological replicates (n = 3). Error bars represent the standard deviation of each mean (±). All FAs were identified using GC-MS based upon retention time, fragmentation, and comparison with standards. Statistical analysis was performed by GraphPad PRISM 6.0 using 2-way ANOVA multiple comparisons based on a Tukey t-test with a 95% confidence interval, where **** is p ≤ 0.0001, *** is p ≤ 0.001, ** is p ≤ 0.01 and * is p ≤ 0.05. Note: ‘ = first eluted isomer; “ = second eluted isomer (S3 Appendix).
(DOCX)

S4 Fig GC-MS analysis of the fatty acids in Tb-Δ6 genetically manipulated *T. brucei* BSF in high-fat media. A) The bar chart shows the different PUFAs (X axis, the order follows increasing retention time) and the relative abundance (Y axis) found in Tb-Δ6 genetically modified *T. brucei* BSF (KD-D6 and OE-D6) and WT control, when the cells are cultured for 48 h in HMI-11 supplemented with 10% FBS, in the presence of tetracycline as shown in the legend. B) The bar chart is an expansion of the region including 20:4 PUFAs from A. C) The bar chart is an expansion of the region including 22:4 and 22:5 PUFAs from A. D) The bar chart is an expansion of the region including 22:6 PUFAs from A. Values are the mean of three independent biological replicates (n = 3). Error bars represent the standard deviation of each mean (±). All FAs were identified using GC-MS based upon retention time, fragmentation, and comparison with standards. Statistical analysis was performed by GraphPad PRISM 6.0 using 2-way ANOVA multiple comparisons based on a Tukey t-test with a 95% confidence interval, where **** is p ≤ 0.0001, *** is p ≤ 0.001, ** is p ≤ 0.01 and * is p ≤ 0.05. Note: ‘ = first eluted isomer; “ = second eluted isomer. (S2 Appendix).
(DOCX)

S5 Fig GC-MS analysis of the fatty acids in Tb-Δ6 genetically manipulated *T. brucei* BSF in low-fat media. A) The bar chart shows the different PUFAs (X axis, the order follows increasing retention time) and the relative abundance (Y axis) found in Tb-Δ6 genetically modified *T. brucei* BSF (KD-D6 and OE-D6) and WT control, when the cells are cultured for 48 h in HMI-11 supplemented with 5% FBS, in the presence of tetracycline as shown in the legend. B) The bar chart is an expansion of the region including 20:4 PUFAs from A. C) The bar chart is an expansion of the region including 22:4 PUFAs from A. D) The bar chart is an expansion of the region including 22:5 PUFAs from A. E) The bar chart is an expansion of the region including 22:6 PUFAs from A. Values are the mean of three independent biological replicates (n = 3). Error bars represent the standard deviation of each mean (±). All FAs were identified using GC-MS based upon retention time, fragmentation, and comparison with standards. Statistical analysis was performed by GraphPad PRISM 6.0 using 2-way ANOVA multiple comparisons based on a Tukey t-test with a 95% confidence interval, where **** is p ≤ 0.0001, *** is p ≤ 0.001, ** is p ≤ 0.01 and * is p ≤ 0.05. Note: ‘ = first eluted isomer; “ = second eluted isomer. (S2 Appendix).
(DOCX)

S6 Fig GC-MS analysis of the fatty acids in Tb-Δ6 genetically manipulated *T. brucei* PCF in high- and low-fat media in the absence of tetracycline. The bar charts show the different PUFAs (X axis, the order follows increasing retention time) and the relative abundance (Y axis) found in Tb-Δ6 genetically modified *T. brucei* PCF (KD-D6, OE-D6, OK-D6) and WT control, when the cells are cultured for 48 h in SDM-79 supplemented with 10% FBS (A) and with 1.25% FBS (B), in the absence of tetracycline as shown in the legend. Values are the mean of three independent biological replicates (n = 3). Error bars represent the standard deviation of each mean (±). All FAs were identified using GC-MS based upon retention time, fragmentation, and comparison with standards. Statistical analysis was performed by GraphPad PRISM 6.0 using 2-way or One-way ANOVA multiple comparisons based on a Tukey t-test with a 95% confidence interval. Note: ‘ = first eluted isomer; “ = second eluted isomer. (S3 Appendix).
(DOCX)

S7 Fig GC-MS analysis of the fatty acids in Tb-Δ6 genetically manipulated *T. brucei* BSF in high- and low-fat media in the absence of tetracycline. The bar charts show the different PUFAs (X axis, the order follows increasing retention time) and the relative abundance (Y axis) found in Tb-Δ6 genetically modified *T. brucei* BSF (KD-D6 and OE-D6) and WT control, when the cells are cultured for 48 h in HMI-11 supplemented with 10% FBS (A) and with 5% FBS (B), in the absence of tetracycline as shown in the legend. Values are the mean of three independent biological replicates (n = 3). Error bars represent the standard deviation of each mean (±). All FAs were identified using GC-MS based upon retention time, fragmentation, and comparison with standards. Statistical analysis was performed by GraphPad PRISM 6.0 using 2-way or One-way ANOVA multiple comparisons based on a Tukey t-test with a 95% confidence interval. Note: ‘ = first eluted isomer; “ = second eluted isomer. (S2 Appendix).
(DOCX)

S8 Fig PCR screening confirms cloning of the gene encoding Tb-Δ6 in p2T7-177-Phleo (A-B) and pLew100-C-term-HA-BSD vectors (C-D). A) The cartoon shows the PCR strategy used to confirm the cloning strategy of the gene encoding Tb-Δ6 in p2T7-177-Phleo. B) Lanes 1-17 are a PCR amplification of Tb-Δ6 from the p2T7-177-Tb-Δ6-Phleo plasmid from *E. coli* single colonies, compared to *E. coli* single colonies containing empty vectors (C1 and C2). Two different primer pairs were used for the amplification. The first product (lanes 1-3-5) is an intense band slightly above 600 bp (expected size 630 bp, orange primers in A). The second product (lanes 8-17) is an intense band between 600-800 bp (expected size 702 bp, green primers in A). C) The cartoon shows the PCR strategy used to confirm the cloning of the gene encoding Tb-Δ6 in pLew100-C-term-HA-BSD. B) Lanes 1-12 are a PCR amplification of the gene encoding Tb-Δ6 from the pLew100-Tb-Δ6-C-term-HA-BSD plasmid from *E. coli* single colonies, compared to *E. coli* single colony containing empty vector (C1). The product (lanes 2-3-4-5) is an intense band at around 1500 bp (expected size 1499 bp).
(DOCX)

S9 Fig PCR amplifications of p2T7-177-Tb-Δ6-Phleo and pLew100-Tb-Δ6-C-term-HA-BSD confirm their integration in the gDNA of *T. brucei* BSF and PCF and change in the level of expression. A, C) PCR amplification of pLew100-Tb-Δ6-C-term-HA-BSD from *T. brucei* BSF and PCF gDNA using primers targeting the gene encoding Tb-Δ6 and BSD. The product is a bright band between 2000-3000 bp (expected size 2257 bp) present in the positive control (P), absent in the wild type (W) and integrated in Δ6-OE BSF (A, lane 1), in Δ6-OE PCF (C, lane 2) and in Δ6-OK PCF (C, lane 3). B, D) PCR amplification of p2T7-177-Tb-Δ6-Phleo from *T. brucei* BSF and PCF gDNA using primers targeting the gene encoding Tb-Δ6 and Phleo. The product is a bright band between 2000-3000 bp (expected size 2605 bp) present in the positive control (P), absent in the wild type (W) and integrated in Δ6-KD BSF (B, lane 1), in Δ6-KD PCF (D, lane 2) and in Δ6-OK PCF (section 3.2.3) (D, lane 1). E) The cartoon shows the PCR strategy used to confirm the integration in the gDNA of p2T7-177-Tb-Δ6-Phleo. F) The cartoon shows the PCR strategy used to confirm the integration in the gDNA of pLew100-Tb-Δ6-C-term-HA-BSD.
(DOCX)

S10 Fig Western blots probed with an anti-HA tag antibody confirm Tb-Δ6 overexpression in *T. brucei* PCF and BSF and immunofluorescence microscopy. A) The figure represents the western blot, probed with an anti-HA tag antibody, of the protein extract from *T. brucei* PCF WT control and *T. brucei* PCF Δ6-desaturase knock down (Δ6-KD), Δ6-desaturase overexpression (Δ6-OE) and Δ6-desaturase add-back (Δ6-OK) cells. The cells were grown for 48 h in SDM-79 with 10% FBS in the presence or absence of Tet and harvested at 1 x 10^7^ cells per sample. The red arrow highlights the confirmed overexpression of Tb-Δ6 in Δ6-OE with a mass of ~49 kDa as predicted. There is no protein detected for Δ6-KD and WT control. B) The figure represents the western blot, probed with an anti-HA tag antibody, of the protein extract from *T. brucei* BSF WT control and *T. brucei* BSF Tb-Δ6 overexpression (Δ6-OE). The cells were grown for 48 h in HMI-11 with 10% FBS in presence or absence of tetracycline and harvested at 1 x 10^7^ cells per sample. The red arrow highlights the confirmed overexpression of Tb-Δ6, ~49 kDa as predicted in Δ6-OE. M, protein marker. Note: Ref. Marker, the markers from lower exposure images of the same western blots are reported to allow better visualisation of M image obtained at higher exposure. C, D) Immunofluorescence microscopy images of *T. brucei* PCF OE-D6 (C) and *T. brucei* BSF OE-D6 (D) fixed on poly-lysine coated slides stained with DAPI (blue signal), MitoTracker Red (red signal), anti-HA tag (green signal) and imaged with DeltaVision Imaging System confocal microscope. The cells were grown for 48 h in HMI-11 with 10% FBS in the absence of tetracycline. Images were processed using softWoRx Explorer 1.3.
(DOCX)

S11 Fig A, B) Growth curves of *T. brucei* BSF in high-fat media. The graphs represent the growth curves over 48 h of *T. brucei* BSF WT control and *T. brucei* BSF Tb-Δ6 knock-down (Δ6-KD), and Tb-Δ6 overexpression (Δ6-OE) cells, when they are cultured in HMI-11 supplemented with 10% FBS in the presence (A) or absence (B) of tetracycline as shown in the legend. C, D) Growth curves of *T. brucei* PCF in high-fat media. The graphs represent the growth curves over 72 h of *T. brucei* PCF WT control and *T. brucei* PCF Tb-Δ6 knock-down (Δ6-KD), Tb-Δ6 overexpression (Δ6-OE) and Tb-Δ6 add-back (Δ6-OK) cells, when they are cultured in SDM-79 supplemented with 10% FBS in the presence (C) or absence (D) of tetracycline, as shown in the legend. E) Growth curves of *T. brucei* BSF in low-fat media. The graphs represent the growth curves over 48 h of *T. brucei* BSF WT control and *T. brucei* BSF Tb-Δ6 knock-down (Δ6-KD), and Tb-Δ6 overexpression (Δ6-OE) cells, when they are cultured in HMI-11 supplemented with 5% FBS in the absence of tetracycline. F) Growth curves of *T. brucei* PCF in low-fat media. The graphs represent the growth curves over 72 h of *T. brucei* PCF WT control and *T. brucei* PCF Tb-Δ6 knock-down (Δ6-KD), Tb-Δ6 overexpression (Δ6-OE) and Tb-Δ6 add-back (Δ6-OK) cells, when they are cultured in SDM-79 supplemented with 1.25% FBS in the absence of tetracycline. For all growth curves values are the mean of three independent biological replicates (n = 3). Error bars represent the standard deviation of each mean. Statistical analysis was performed by GraphPad PRISM 6.0 using One-way ANOVA multiple comparisons based on a Tukey t-test with a 95% confidence interval.
(DOCX)

S12 Fig Growth curves of Tb-Δ6 genetically modified *T. brucei* PCF and BSF supplemented with DHA in the absence of tetracycline. The graphs represent the growth curves over 48 h of *T. brucei* PCF (A) and BSF (B) WT control and *T. brucei* Δ6-desaturase knock down (KD-D6), when they are cultured in SDM-79 supplemented with 1.25% FBS (A) and HMI-11 supplemented with 5% FBS (B) both added with 10 µM DHA (22:6) (dotted/dashed lines), in the absence of tetracycline as shown in the legend. Values are the mean of three independent biological replicates (n = 3). Error bars represent the standard deviation of each mean (±). All FAs were identified using GC-MS based upon retention time, fragmentation, and comparison with standards. Statistical analysis was performed by GraphPad PRISM 6.0 using One-way ANOVA multiple comparisons based on a Tukey t-test with a 95% confidence interval. Note: the solid lines represent data taken from S11E and S11F Fig used here for a more complete comparison.
(DOCX)

S13 Fig GC-MS analysis of the fatty acids in Tb-Δ6 genetically manipulated *T. brucei* PCF in low-fat media supplemented with DHA. A) The bar chart shows the different PUFAs (X axis, the order follows increasing retention time) and the relative abundance (Y axis) found in Tb-Δ6 genetically modified *T. brucei* PCF (KD-D6) and WT control, when the cells are cultured for 48 h in SDM-79 with 1.25% FBS supplemented with 10 µM DHA, in the presence or absence of Tet as shown in the legend. B) The bar chart is an expansion of the region including 22:6 PUFAs from A. C) The bar chart is an expansion of the region including 20:4 PUFAs from A. D) The bar chart is an expansion of the region including 22:4 from A. E) The bar chart is an expansion of the region including 22:5 PUFAs from A. Values are the mean of three independent biological replicates (n = 3). Error bars represent the standard deviation of each mean (±). All FAs were identified using GC-MS based upon retention time, fragmentation, and comparison with standards. Statistical analysis was performed by GraphPad PRISM 6.0 using 2-way or One-way ANOVA multiple comparisons based on a Tukey t-test with a 95% confidence interval, where **** is p ≤ 0.0001, *** is p ≤ 0.001, ** is p ≤ 0.01 and * is p ≤ 0.05. Note: ‘ = first eluted isomer; “ = second eluted isomer. (S3 Appendix).
(DOCX)

S14 Fig GC-MS analysis of the fatty acids in Tb-Δ6 genetically manipulated *T. brucei* BSF in low-fat media supplemented with DHA. A) The bar chart shows the different PUFAs (X axis, the order follows increasing retention time) and the relative abundance (Y axis) found in Tb-Δ6 genetically modified *T. brucei* BSF (KD-D6) and WT control, when the cells are cultured for 48 h in HMI-11 with 5% FBS supplemented with 10 µM DHA, in the presence or absence of Tet as shown in the legend. B) The bar chart is an expansion of the region including 22:6 PUFAs from A. C) The bar chart is an expansion of the region including 20:4 PUFAs from A. D) The bar chart is an expansion of the region including 22:4 from A. E) The bar chart is an expansion of the region including 22:5 PUFAs from A. Values are the mean of three independent biological replicates (n = 3). Error bars represent the standard deviation of each mean (±). All FAs were identified using GC-MS based upon retention time, fragmentation, and comparison with standards. Statistical analysis was performed by GraphPad PRISM 6.0 using 2-way or One-way ANOVA multiple comparisons based on a Tukey t-test with a 95% confidence interval, where **** is p ≤ 0.0001, *** is p ≤ 0.001, ** is p ≤ 0.01 and * is p ≤ 0.05. Note: ‘ = first eluted isomer; “ = second eluted isomer. (S2 Appendix).
(DOCX)

S15 Fig GC-MS analysis of the 18C fatty acids in *T. brucei* BSF and PCF WT in low-fat media supplemented with DHA. A) The bar chart shows the different 18C FAs (X axis, the order follows increasing retention time) and the relative abundance (Y axis) found in *T. brucei* BSF (A, B and C) and PCF (D, E and F) WT controls, when the cells are cultured for 48 h in HMI-11 with 5% FBS (A, B and C) or SDM-79 with 1.25% FBS (D, E and F) supplemented with 10 µM DHA, as shown in the legend, and compared to *T. brucei* BSF and PCF WT in HMI-11 or SDM-79 with 10% FBS. Values are the mean of three independent biological replicates (n = 3). Error bars represent the standard deviation of each mean (±). All FAs were identified using GC-MS based upon retention time, fragmentation, and comparison with standards. Statistical analysis was performed by GraphPad PRISM 6.0 using One-way ANOVA multiple comparisons based on a Tukey t-test with a 95% confidence interval, where **** is p ≤ 0.0001, ** is p ≤ 0.01 and * is p ≤ 0.05. Note: ‘ = first eluted isomer; “ = second eluted isomer. (S2 and S3 Appendices).
(DOCX)

S16 Fig A–D) ESI-MS/MS spectra of PI-containing lipids for Tb-Δ6 genetically manipulated *T. brucei* BSF grown in high- and low-fat media. The spectra show PI-containing lipids obtained by scanning for parent ion of 241 m/z for Δ6-KD BSF (A) grown for 48 h in HMI-11 with 5% FBS in the presence of tetracycline, and WT control BSF (B), Δ6-KD BSF (C) and Δ6-OE BSF (D) grown in HMI-11 with 10% FBS. The species of interest are labelled (PIs black) as reported in the text and highlighted by arrows (PIs black). Spectra are representative of experiments conducted in three independent biological replicates (n = 3). E) ESI-MS/MS quantification of PIs in the knock-down of Tb-Δ6 in *T. brucei* PCF in low-fat media. The bar charts show the difference in PI and IPC species (X axis) and the normalised intensity (Y axis, cps) found in *T. brucei* PCF KD-D6 and WT control, when the cells are cultured for 48 h in HMI-11 supplemented with 1.25% FBS, in the presence of tetracycline as shown in the legend. The relative intensities of PIs were normalised against the intensity of PI (15:0/18:1(d7)) at 847.13 m/z contained in SPLASH internal standard. Values are the mean of three independent biological replicates (n = 3). Standard deviation of each mean (±) is calculated for the normalised intensities. Statistical analysis was performed by GraphPad PRISM 6.0 using One-way ANOVA multiple comparisons based on a Tukey t-test with a 95% confidence interval, where ** is p ≤ 0.01 and * is p ≤ 0.05. (S4 Appendix) E) Inositol-phosphoryl ceramide (IPC) and sphingomyelin (SM) synthetic pathway in *T. brucei*. Schematic representation of the synthesis of IPC from ceramide and PI by inositol-phosphoryl ceramide synthase (SLS1) and of SM from ceramide and PC by sphingomyelin synthase (SLS4), through release of diacylglycerol (DAG). R1 and R2 represent the fatty chain.
(DOCX)

S17 Fig A) Fragmentation pattern of PI (16:0/14:0) and IPC (d16:0/18:0). Daughter ion scans from Δ6-OE BSF (grown in low fat media) in negative mode for PI and IPC at m/z 780. B) Fragmentation pattern of IPC (t16:0/18:1). Daughter ion scans from Δ6-OE BSF (grown in low fat media) in negative mode for IPC at m/z 794. C) Fragmentation patterns of PI (16:0/18:0) and IPC (d20:0/18:1). Daughter ion scans from Δ6-OE BSF (grown in low fat media) in negative mode for PI and IPC at m/z 835/837. D) Fragmentation pattern of SM (d16:0/18:0). Daughter ion scans from Δ6-OE BSF (grown in low fat media) in positive mode for SM at m/z 706. The structures have coloured portions to highghlight the correspondent fragments in the spectra indicated with circles of the same colour.
(DOCX)

S18 Fig A-C) ESI-MS/MS spectra of PC-containing lipids for Tb-Δ6 genetically manipulated *T. brucei* BSF grown in low-fat media. The spectra show choline-phosphate-containing lipids obtained by scanning for parent ion of 184 m/z for WT control BSF (A) and Δ6-KD BSF (B) and Δ6-OE BSF (C) grown for 48 h in HMI-11 with 5% FBS, in the presence of Tet. The SM of interest is labelled as reported in the text and highlighted by an arrow (red). The major SM and PC species are also annotated. a, alkylacyl. Spectra are representative of experiments conducted in three independent biological replicates (n = 3). D) ESI-MS/MS quantification of sphingomyelin in Tb-Δ6 genetically manipulated *T. brucei* BSF in low-fat media. The bar chart shows the difference in SM species at 706 m/z (X axis) and the normalised intensity (Y axis, cps) found in Tb-Δ6 genetically modified *T. brucei* BSF (OE-D6) and WT control, when the cells are cultured for 48 h in HMI-11 supplemented with 5% FBS, in the presence of tetracycline and treated or not with EC_10_ of clemastine fumarate, as shown in the legend. The relative intensities of IPCs were normalised against the intensity of PI (15:0/18:1(d7)) at 847.13 m/z contained in SPLASH internal standard. Values are the mean of three independent biological replicates (n = 3). Standard deviation of each mean (±) is calculated for the normalised intensities. Statistical analysis was performed by GraphPad PRISM 6.0 using One-way ANOVA multiple comparisons based on a Tukey t-test with a 95% confidence interval. (S4 Appendix).
(DOCX)

S19 Fig Dose-response assay for clemastine fumarate against Tb-Δ6 genetically manipulated *T. brucei* BSF grown in low-fat media. Dose-response curve and correspondent table of EC_50_ values obtained for clemastine fumarate against WT *T. brucei* BSF (A), Δ6-OE *T. brucei* BSF induced with Tet for 48 h (B) and Δ6-OE *T. brucei* BSF non-induced with Tet (C) grown in HMI-11 with 5% of FBS. Data analysis was carried using GraFit 5.0 (Erithacus Software).
(DOCX)

S20 Fig SLS1 and SLS4 sequences identification via SWATH MS. SLS4 (A) and SLS1 (B) protein sequence are reported as presented in the proteomics analytical software MASCOT. Red highlights represent the identified peptides.
(DOCX)
