# Supplementary material for: Exploring the activity of the putative Δ6-desaturase and its role in bloodstream form life-cycle transitions in Trypanosoma brucei
Source: PLoS Pathog. 2025 Feb 18;21(2):e1012691. doi: 10.1371/journal.ppat.1012691 (PMC11867338; doi:10.1371/journal.ppat.1012691)
Supplement: S1 Table — The table shows a summary of the relative abundance and the retention times of the FAMEs or FAs after GC-MS analysis of samples obtained from FBS. Values are the mean of three independent biological replicates (n = 3). SD is standard deviation of each mean (±) (S1 Appendix). (DOCX) [file ppat.1012691.s010.docx]

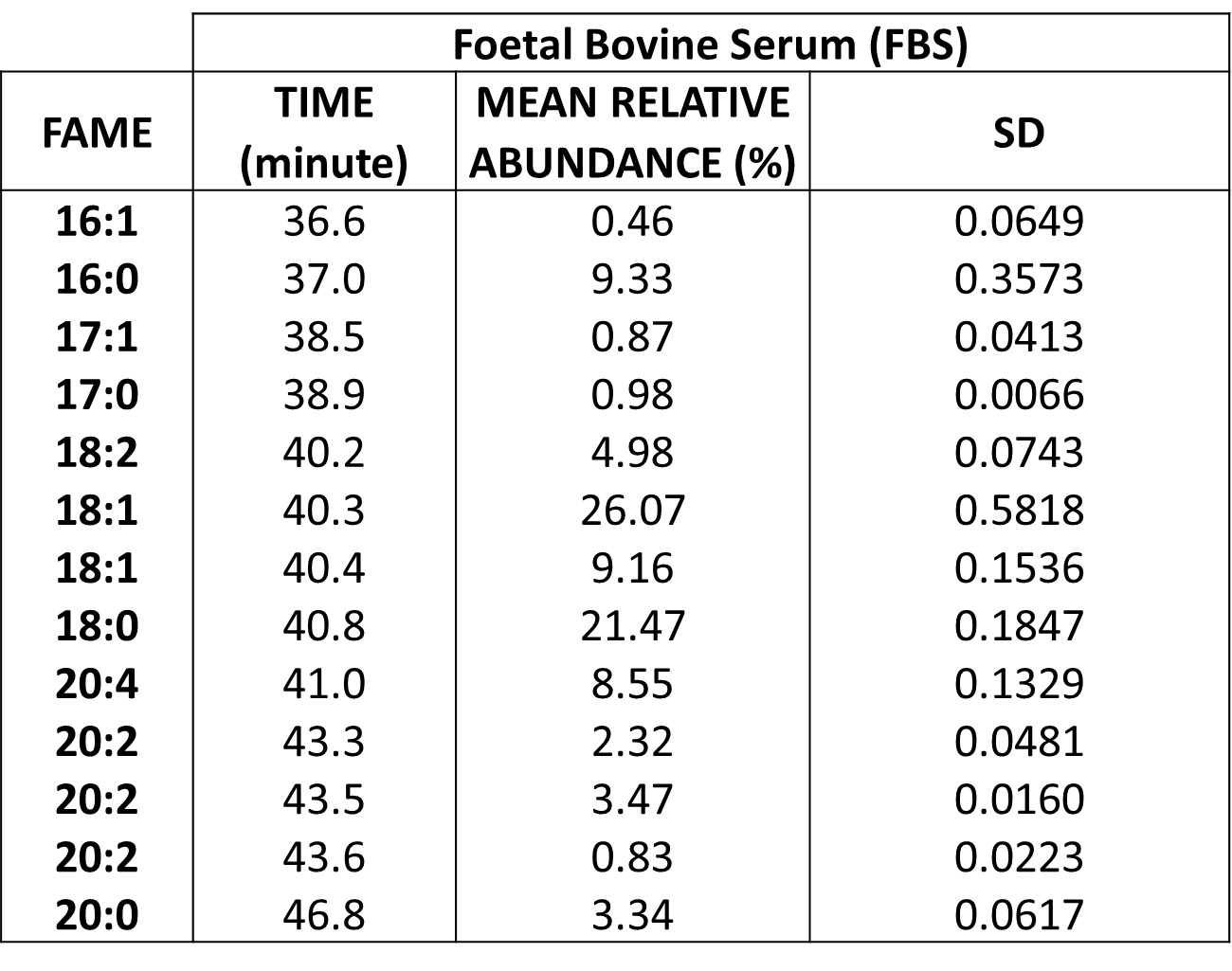


S1 Table. Fatty acid content of foetal bovine serum (FBS) used to supplement SDM-79 and HMI-11 media. The table shows a summary of the relative abundance and the retention times of the FAMEs or FAs after GC-MS analysis of samples obtained from FBS. Values are the mean of three independent biological replicates (n=3). SD is standard deviation of each mean (±) (Appendix A).
