# Supplementary material for: Exploring the activity of the putative Δ6-desaturase and its role in bloodstream form life-cycle transitions in Trypanosoma brucei
Source: PLoS Pathog. 2025 Feb 18;21(2):e1012691. doi: 10.1371/journal.ppat.1012691 (PMC11867338; doi:10.1371/journal.ppat.1012691)
Supplement: S1 Fig — GC-MS chromatogram analysis of T. brucei PCF WT cultured for 48 h in SDM-79 with 1.25% FBS (A) and T. brucei BSF WT cultured in HMI-11 with 5% FBS (B). Peaks eluted at different retention times (X axis) and with different abundance (Y axis), are assigned to specific FAs. The C20 (green (A) and light blue (B) brackets and inserts) and C22 PUFAs (orange (A) and purple (B) bracket and insert) are expanded. The red lines indicate the major species of FAs. Note: ‘= first eluted isomer; “= second eluted isomer; first and second eluted isomers are FAs with same number of C on the alkyl chain, and the same number of double bonds in different position along the alkyl chain The chromatograms are representative of experiment results conducted in three independent biological replicates (n = 3). (DOCX) [file ppat.1012691.s011.docx]

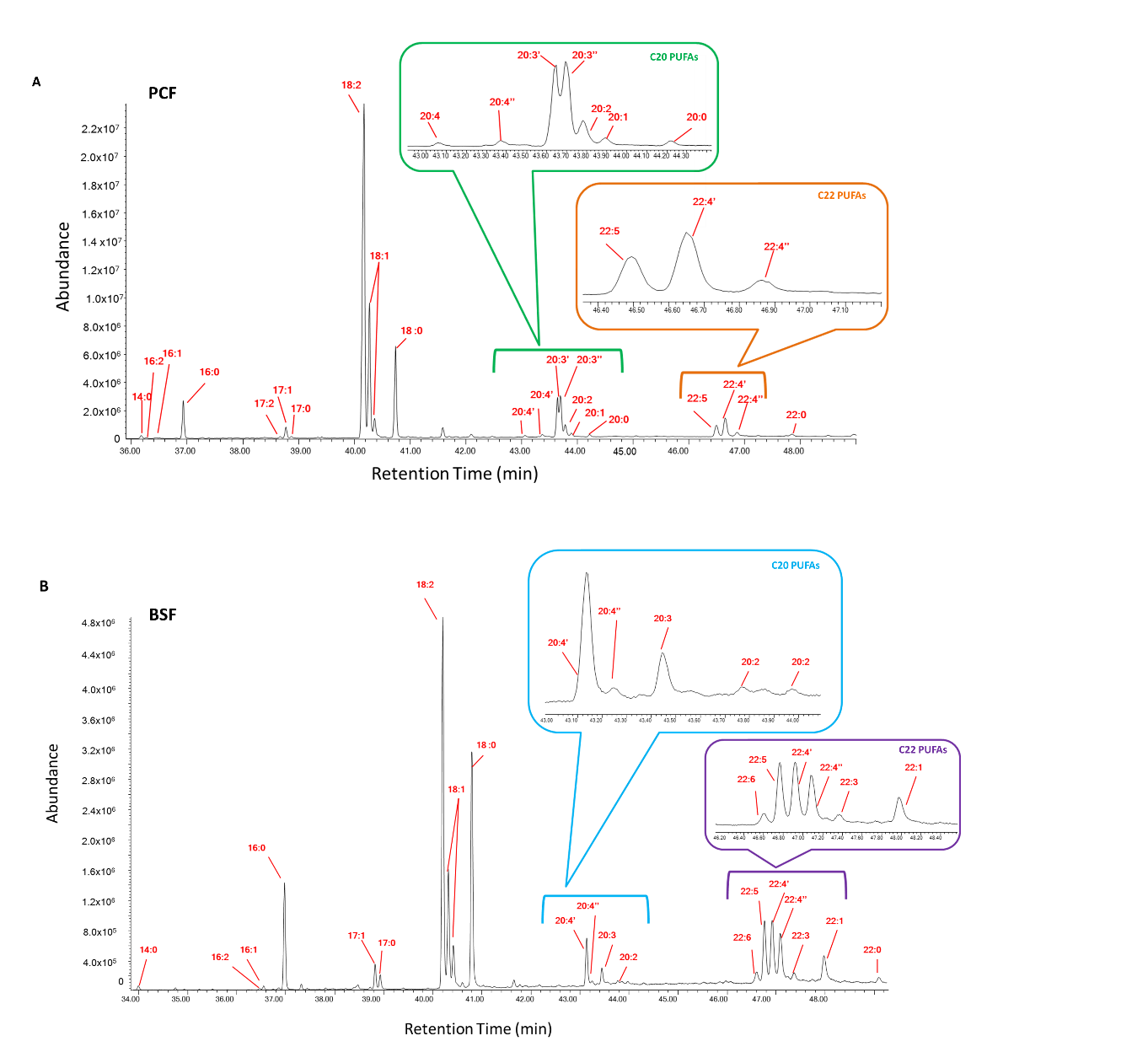


S1 Fig. GC-MS analysis of the FAMEs in *T. brucei* PCF and BSF. GC-MS chromatogram analysis of *T. brucei* PCF WT cultured for 48 h in SDM-79 with 1.25% FBS (A) and *T. brucei* BSF WT cultured for 48 h in HMI-11 with 5% FBS (B). Peaks eluted at different retention times (X axis) and with different abundance (Y axis), are assigned to specific FAs. The C20 (green (A) and light blue (B) brackets and inserts) and C22 PUFAs (orange (A) and purple (B) bracket and insert) are expanded. The red lines indicate the major species of FAs. Note: ‘ = first eluted isomer; “ = second eluted isomer; first and second eluted isomers are FAs with same number of C on the alkyl chain, and the same number of double bonds in different position along the alkyl chain. The chromatograms are representative of experiment results conducted in three independent biological replicates (n = 3).
