# Supplementary material for: Exploring the activity of the putative Δ6-desaturase and its role in bloodstream form life-cycle transitions in Trypanosoma brucei
Source: PLoS Pathog. 2025 Feb 18;21(2):e1012691. doi: 10.1371/journal.ppat.1012691 (PMC11867338; doi:10.1371/journal.ppat.1012691)
Supplement: S3 Fig — A) The bar chart shows the different PUFAs (X axis, the order follows increasing retention time) and the relative abundance (Y axis) found in Tb-Δ6 genetically modified T. brucei PCF (KD-D6, OE-D6, OK-D6) and WT control, when the cells are cultured for 48 h in SDM-79 supplemented with 1.25% FBS, in the presence of tetracycline as shown in the legend. B) The bar chart is an expansion of the region including 20:4 PUFAs from A. C) The bar chart is an expansion of the region including 22:4, 22:5, 22:6 PUFAs from A. Values are the mean of three independent biological replicates (n = 3). Error bars represent the standard deviation of each mean (±). All FAs were identified using GC-MS based upon retention time, fragmentation, and comparison with standards. Statistical analysis was performed by GraphPad PRISM 6.0 using 2-way ANOVA multiple comparisons based on a Tukey t-test with a 95% confidence interval, where **** is p ≤ 0.0001, *** is p ≤ 0.001, ** is p ≤ 0.01 and * is p ≤ 0.05. Note: ‘ = first eluted isomer; “ = second eluted isomer (S3 Appendix). (DOCX) [file ppat.1012691.s013.docx]

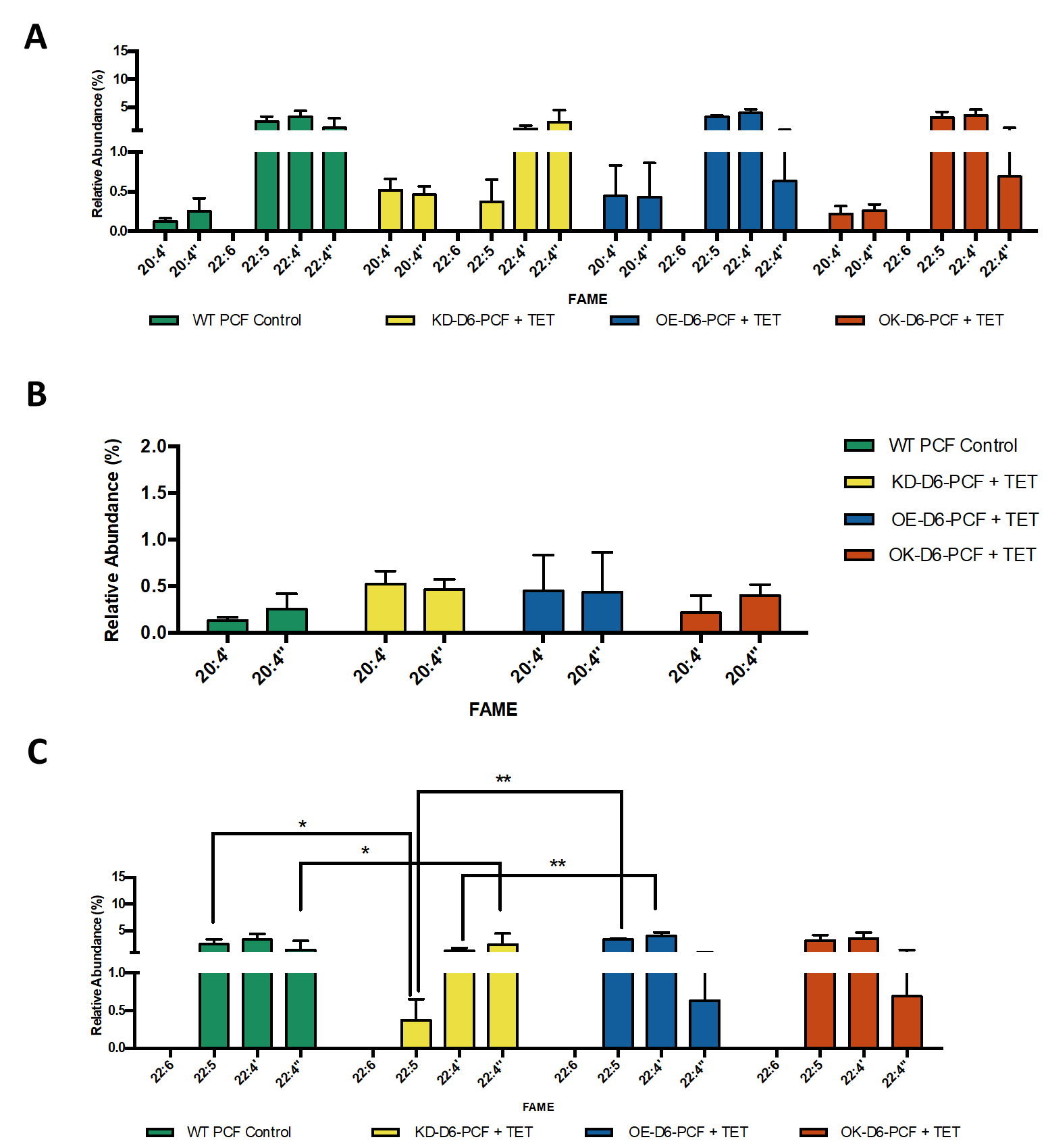


S3 Fig. GC-MS analysis of the fatty acids in Tb-Δ6 genetically manipulated *T. brucei* PCF in low-fat media.  A) The bar chart shows the different PUFAs (X axis, the order follows increasing retention time) and the relative abundance (Y axis) found in Tb-Δ6 genetically modified *T. brucei* PCF (KD-D6, OE-D6, OK-D6) and WT control, when the cells are cultured for 48 h in SDM-79 supplemented with 1.25% FBS, in the presence of tetracycline as shown in the legend. B) The bar chart is an expansion of the region including 20:4 PUFAs from A. C) The bar chart is an expansion of the region including 22:4, 22:5, 22:6 PUFAs from A. Values are the mean of three independent biological replicates (n=3). Error bars represent the standard deviation of each mean (±). All FAs were identified using GC-MS based upon retention time, fragmentation, and comparison with standards. Statistical analysis was performed by GraphPad PRISM 6.0 using 2-way ANOVA multiple comparisons based on a Tukey t-test with a 95% confidence interval, where **** is p ≤ 0.0001, *** is p ≤ 0.001, ** is p ≤ 0.01 and * is p ≤ 0.05. Note: ‘ = first eluted isomer; “ = second eluted isomer (Appendix C).
