# Supplementary material for: Exploring the activity of the putative Δ6-desaturase and its role in bloodstream form life-cycle transitions in Trypanosoma brucei
Source: PLoS Pathog. 2025 Feb 18;21(2):e1012691. doi: 10.1371/journal.ppat.1012691 (PMC11867338; doi:10.1371/journal.ppat.1012691)
Supplement: S6 Fig — The bar charts show the different PUFAs (X axis, the order follows increasing retention time) and the relative abundance (Y axis) found in Tb-Δ6 genetically modified T. brucei PCF (KD-D6, OE-D6, OK-D6) and WT control, when the cells are cultured for 48 h in SDM-79 supplemented with 10% FBS (A) and with 1.25% FBS (B), in the absence of tetracycline as shown in the legend. Values are the mean of three independent biological replicates (n = 3). Error bars represent the standard deviation of each mean (±). All FAs were identified using GC-MS based upon retention time, fragmentation, and comparison with standards. Statistical analysis was performed by GraphPad PRISM 6.0 using 2-way or One-way ANOVA multiple comparisons based on a Tukey t-test with a 95% confidence interval. Note: ‘ = first eluted isomer; “ = second eluted isomer. (S3 Appendix). (DOCX) [file ppat.1012691.s016.docx]

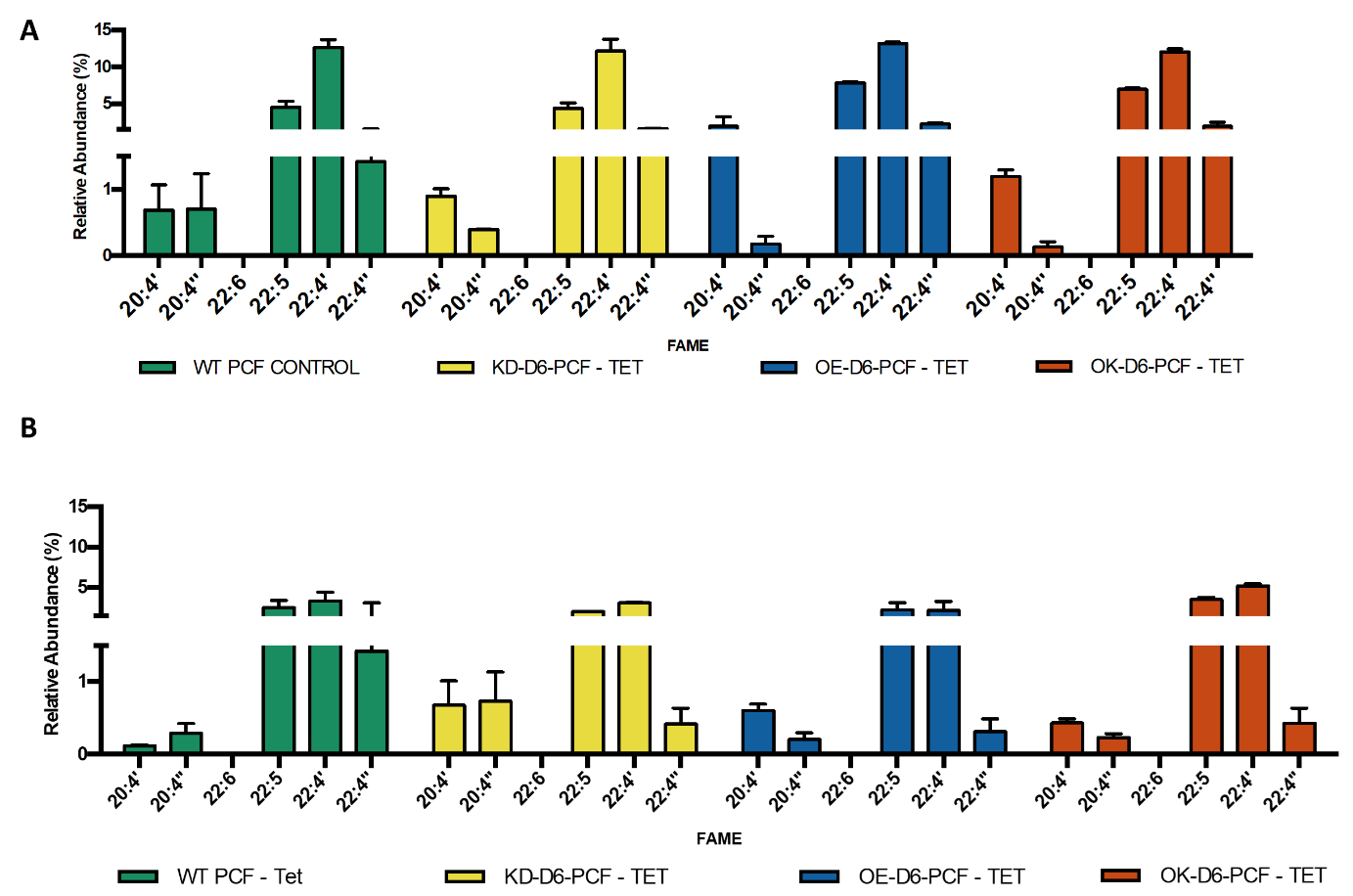


S6 Fig. GC-MS analysis of the fatty acids in Tb-Δ6 genetically manipulated *T. brucei* PCF in high- and low-fat media in the absence of tetracycline.  The bar charts show the different PUFAs (X axis, the order follows increasing retention time) and the relative abundance (Y axis) found in Tb-Δ6 genetically modified *T. brucei* PCF (KD-D6, OE-D6, OK-D6) and WT control, when the cells are cultured for 48 h in SDM-79 supplemented with 10% FBS (A) and with 1.25% FBS (B), in the absence of tetracycline as shown in the legend. Values are the mean of three independent biological replicates (n=3). Error bars represent the standard deviation of each mean (±). All FAs were identified using GC-MS based upon retention time, fragmentation, and comparison with standards. Statistical analysis was performed by GraphPad PRISM 6.0 using 2-way or One-way ANOVA multiple comparisons based on a Tukey t-test with a 95% confidence interval. Note: ‘ = first eluted isomer; “ = second eluted isomer. (Appendix C)
