# Supplementary material for: Exploring the activity of the putative Δ6-desaturase and its role in bloodstream form life-cycle transitions in Trypanosoma brucei
Source: PLoS Pathog. 2025 Feb 18;21(2):e1012691. doi: 10.1371/journal.ppat.1012691 (PMC11867338; doi:10.1371/journal.ppat.1012691)
Supplement: S8 Fig — A) The cartoon shows the PCR strategy used to confirm the cloning strategy of the gene encoding Tb-Δ6 in p2T7-177-Phleo. B) Lanes 1-17 are a PCR amplification of Tb-Δ6 from the p2T7-177-Tb-Δ6-Phleo plasmid from E. coli single colonies, compared to E. coli single colonies containing empty vectors (C1 and C2). Two different primer pairs were used for the amplification. The first product (lanes 1-3-5) is an intense band slightly above 600 bp (expected size 630 bp, orange primers in A). The second product (lanes 8-17) is an intense band between 600-800 bp (expected size 702 bp, green primers in A). C) The cartoon shows the PCR strategy used to confirm the cloning of the gene encoding Tb-Δ6 in pLew100-C-term-HA-BSD. B) Lanes 1-12 are a PCR amplification of the gene encoding Tb-Δ6 from the pLew100-Tb-Δ6-C-term-HA-BSD plasmid from E. coli single colonies, compared to E. coli single colony containing empty vector (C1). The product (lanes 2-3-4-5) is an intense band at around 1500 bp (expected size 1499 bp). (DOCX) [file ppat.1012691.s018.docx]

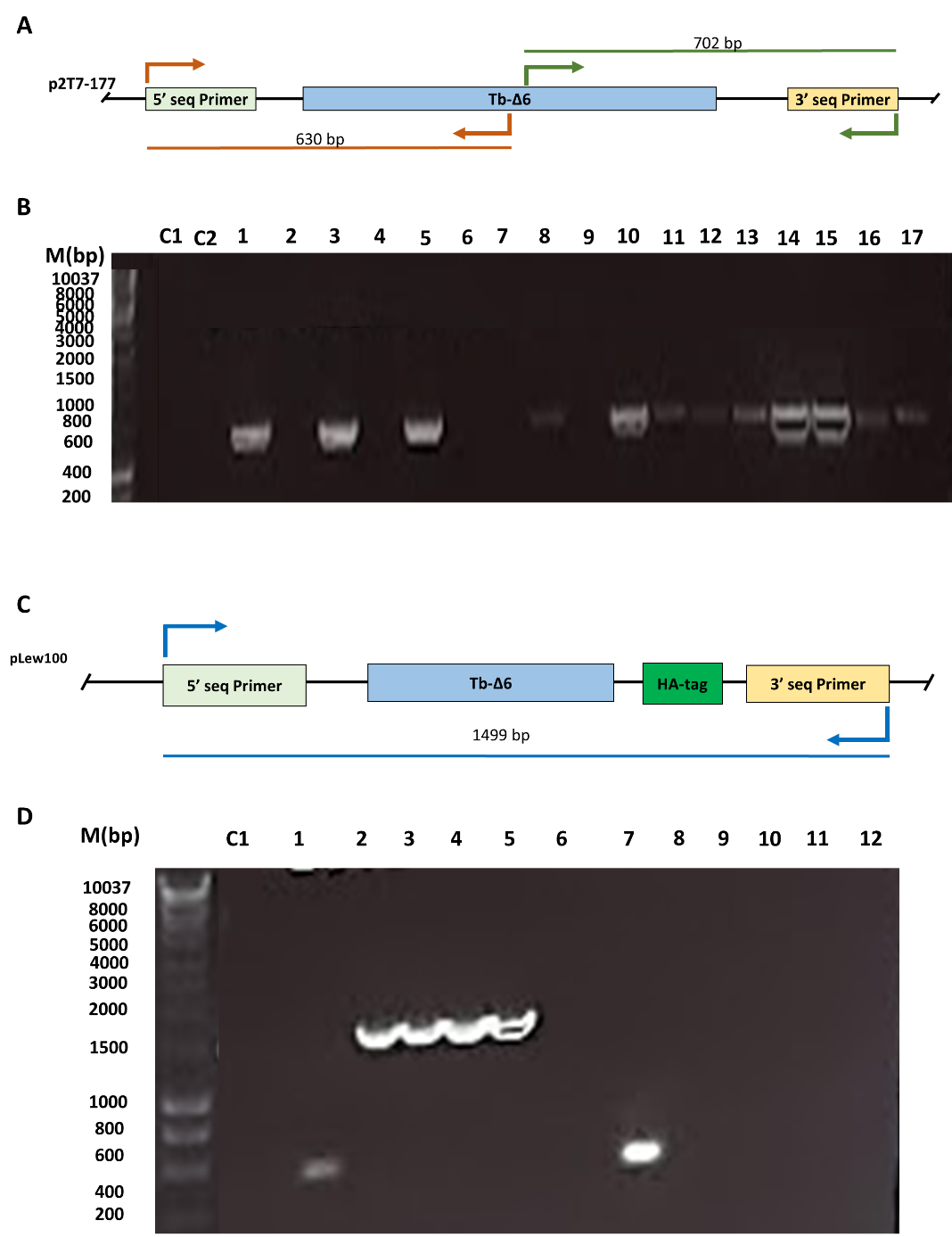


S8 Fig: PCR screening confirms cloning of the gene encoding Tb-Δ6 in p2T7-177-Phleo (A-B) and pLew100-C-term-HA-BSD vectors (C-D).  A) The cartoon shows the PCR strategy used to confirm the cloning strategy of the gene encoding Tb-Δ6 in p2T7-177-Phleo. B) Lanes 1-17 are a PCR amplification of Tb-Δ6 from the p2T7-177-Tb-Δ6-Phleo plasmid from *E. coli* single colonies, compared to *E. coli* single colonies containing empty vectors (C1 and C2). Two different primer pairs were used for the amplification. The first product (lanes 1-3-5) is an intense band slightly above 600 bp (expected size 630 bp, orange primers in A). The second product (lanes 8-17) is an intense band between 600-800 bp (expected size 702 bp, green primers in A). C) The cartoon shows the PCR strategy used to confirm the cloning of the gene encoding Tb-Δ6 in pLew100-C-term-HA-BSD. B) Lanes 1-12 are a PCR amplification of the gene encoding Tb-Δ6 from the pLew100-Tb-Δ6-C-term-HA-BSD plasmid from *E. coli* single colonies, compared to *E. coli* single colony containing empty vector (C1). The product (lanes 2-3-4-5) is an intense band at around 1500 bp (expected size 1499 bp).
