# Supplementary material for: Exploring the activity of the putative Δ6-desaturase and its role in bloodstream form life-cycle transitions in Trypanosoma brucei
Source: PLoS Pathog. 2025 Feb 18;21(2):e1012691. doi: 10.1371/journal.ppat.1012691 (PMC11867338; doi:10.1371/journal.ppat.1012691)
Supplement: S9 Fig — A, C) PCR amplification of pLew100-Tb-Δ6-C-term-HA-BSD from T. brucei BSF and PCF gDNA using primers targeting the gene encoding Tb-Δ6 and BSD. The product is a bright band between 2000-3000 bp (expected size 2257 bp) present in the positive control (P), absent in the wild type (W) and integrated in Δ6-OE BSF (A, lane 1), in Δ6-OE PCF (C, lane 2) and in Δ6-OK PCF (C, lane 3). B, D) PCR amplification of p2T7-177-Tb-Δ6-Phleo from T. brucei BSF and PCF gDNA using primers targeting the gene encoding Tb-Δ6 and Phleo. The product is a bright band between 2000-3000 bp (expected size 2605 bp) present in the positive control (P), absent in the wild type (W) and integrated in Δ6-KD BSF (B, lane 1), in Δ6-KD PCF (D, lane 2) and in Δ6-OK PCF (section 3.2.3) (D, lane 1). E) The cartoon shows the PCR strategy used to confirm the integration in the gDNA of p2T7-177-Tb-Δ6-Phleo. F) The cartoon shows the PCR strategy used to confirm the integration in the gDNA of pLew100-Tb-Δ6-C-term-HA-BSD. (DOCX) [file ppat.1012691.s019.docx]

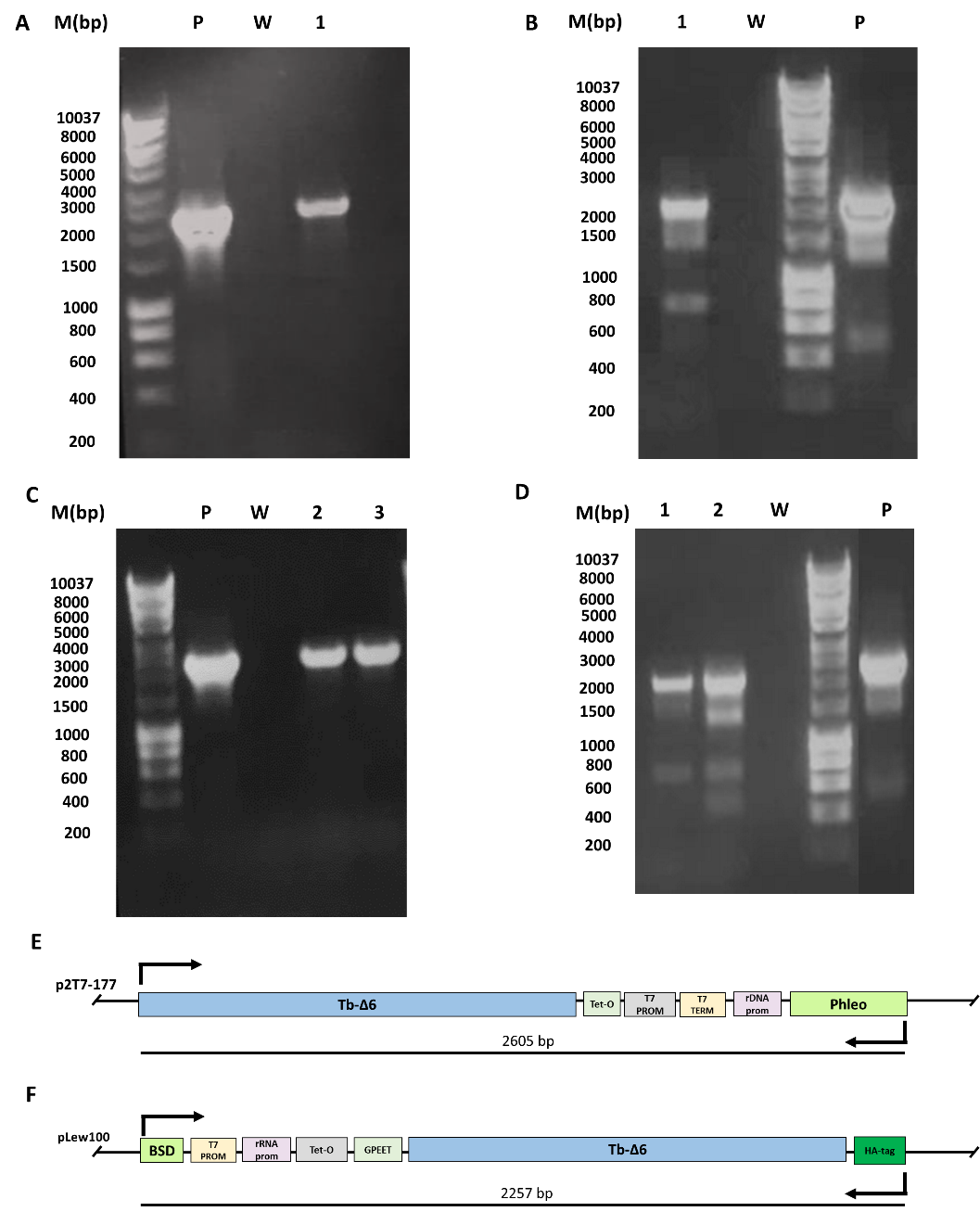


S9 Fig. PCR amplifications of p2T7-177-Tb-Δ6-Phleo and pLew100-Tb-Δ6-C-term-HA-BSD confirm their integration in the gDNA of *T. brucei* BSF and PCF and change in the level of expression.  A, C) PCR amplification of pLew100-Tb-Δ6-C-term-HA-BSD from *T. brucei* BSF and PCF gDNA using primers targeting the gene encoding Tb-Δ6 and BSD. The product is a bright band between 2000-3000 bp (expected size 2257 bp) present in the positive control (P), absent in the wild type (W) and integrated in Δ6-OE BSF (A, lane 1), in Δ6-OE PCF (C, lane 2) and in Δ6-OK PCF (C, lane 3). B, D) PCR amplification of p2T7-177-Tb-Δ6-Phleo from *T. brucei* BSF and PCF gDNA using primers targeting the gene encoding Tb-Δ6 and Phleo. The product is a bright band between 2000-3000 bp (expected size 2605 bp) present in the positive control (P), absent in the wild type (W) and integrated in Δ6-KD BSF (B, lane 1), in Δ6-KD PCF (D, lane 2) and in Δ6-OK PCF (section 3.2.3) (D, lane 1). E) The cartoon shows the PCR strategy used to confirm the integration in the gDNA of p2T7-177-Tb-Δ6-Phleo. F) The cartoon shows the PCR strategy used to confirm the integration in the gDNA of pLew100-Tb-Δ6-C-term-HA-BSD.
