# Supplementary material for: Exploring the activity of the putative Δ6-desaturase and its role in bloodstream form life-cycle transitions in Trypanosoma brucei
Source: PLoS Pathog. 2025 Feb 18;21(2):e1012691. doi: 10.1371/journal.ppat.1012691 (PMC11867338; doi:10.1371/journal.ppat.1012691)
Supplement: S10 Fig — A) The figure represents the western blot, probed with an anti-HA tag antibody, of the protein extract from T. brucei PCF WT control and T. brucei PCF Δ6-desaturase knock down (Δ6-KD), Δ6-desaturase overexpression (Δ6-OE) and Δ6-desaturase add-back (Δ6-OK) cells. The cells were grown for 48 h in SDM-79 with 10% FBS in the presence or absence of Tet and harvested at 1 x 10^7^ cells per sample. The red arrow highlights the confirmed overexpression of Tb-Δ6 in Δ6-OE with a mass of ~49 kDa as predicted. There is no protein detected for Δ6-KD and WT control. B) The figure represents the western blot, probed with an anti-HA tag antibody, of the protein extract from T. brucei BSF WT control and T. brucei BSF Tb-Δ6 overexpression (Δ6-OE). The cells were grown for 48 h in HMI-11 with 10% FBS in presence or absence of tetracycline and harvested at 1 x 10^7^ cells per sample. The red arrow highlights the confirmed overexpression of Tb-Δ6, ~49 kDa as predicted in Δ6-OE. M, protein marker. Note: Ref. Marker, the markers from lower exposure images of the same western blots are reported to allow better visualisation of M image obtained at higher exposure. C, D) Immunofluorescence microscopy images of T. brucei PCF OE-D6 (C) and T. brucei BSF OE-D6 (D) fixed on poly-lysine coated slides stained with DAPI (blue signal), MitoTracker Red (red signal), anti-HA tag (green signal) and imaged with DeltaVision Imaging System confocal microscope. The cells were grown for 48 h in HMI-11 with 10% FBS in the absence of tetracycline. Images were processed using softWoRx Explorer 1.3. (DOCX) [file ppat.1012691.s020.docx]

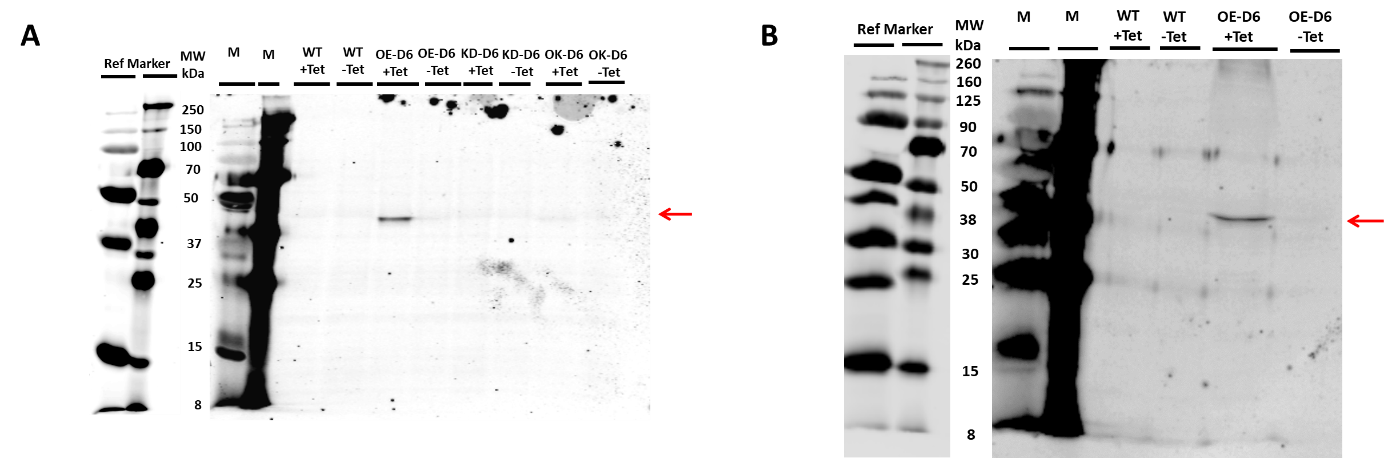


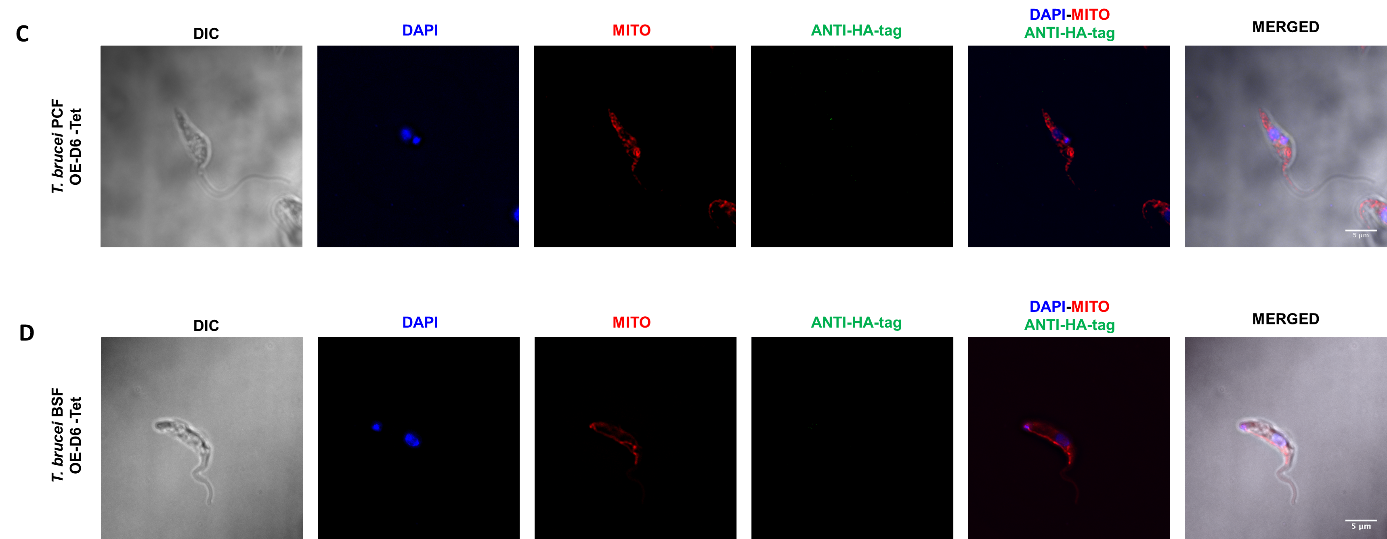


S10 Fig. Western blots probed with an anti-HA tag antibody confirm Tb-Δ6 overexpression in *T. brucei* PCF and BSF and immunofluorescence microscopy.  A) The figure represents the western blot, probed with an anti-HA tag antibody, of the protein extract from *T. brucei* PCF WT control and *T. brucei* PCF Δ6-desaturase knock down (Δ6-KD), Δ6-desaturase overexpression (Δ6-OE) and Δ6-desaturase add-back (Δ6-OK) cells. The cells were grown for 48 h in SDM-79 with 10% FBS in the presence or absence of Tet and harvested at 1 x 10^7^ cells per sample. The red arrow highlights the confirmed overexpression of Tb-Δ6 in Δ6-OE with a mass of ̴ 49 kDa as predicted. There is no protein detected for Δ6-KD and WT control. B) The figure represents the western blot, probed with an anti-HA tag antibody, of the protein extract from *T. brucei* BSF WT control and *T. brucei* BSF Tb-Δ6 overexpression (Δ6-OE). The cells were grown for 48 h in HMI-11 with 10% FBS in presence or absence of tetracycline and harvested at 1 x 10^7^ cells per sample. The red arrow highlights the confirmed overexpression of Tb-Δ6 , ~49 kDa as predicted in Δ6-OE. M, protein marker. Note: Ref. Marker, the markers from lower exposure images of the same western blots are reported to allow better visualisation of M image obtained at higher exposure. C, D) Immunofluorescence microscopy images of *T. brucei* PCF OE-D6 (C) and *T. brucei* BSF OE-D6 (D) fixed on poly-lysine coated slides stained with DAPI (blue signal), MitoTracker Red (red signal), anti-HA tag (green signal) and imaged with DeltaVision Imaging System confocal microscope. The cells were grown for 48 h in HMI-11 with 10% FBS in the absence of tetracycline. Images were processed using softWoRx Explorer 1.3.
