# Supplementary material for: Exploring the activity of the putative Δ6-desaturase and its role in bloodstream form life-cycle transitions in Trypanosoma brucei
Source: PLoS Pathog. 2025 Feb 18;21(2):e1012691. doi: 10.1371/journal.ppat.1012691 (PMC11867338; doi:10.1371/journal.ppat.1012691)
Supplement: S11 Fig — The graphs represent the growth curves over 48 h of T. brucei BSF WT control and T. brucei BSF Tb-Δ6 knock-down (Δ6-KD), and Tb-Δ6 overexpression (Δ6-OE) cells, when they are cultured in HMI-11 supplemented with 10% FBS in the presence (A) or absence (B) of tetracycline as shown in the legend. C, D) Growth curves of T. brucei PCF in high-fat media. The graphs represent the growth curves over 72 h of T. brucei PCF WT control and T. brucei PCF Tb-Δ6 knock-down (Δ6-KD), Tb-Δ6 overexpression (Δ6-OE) and Tb-Δ6 add-back (Δ6-OK) cells, when they are cultured in SDM-79 supplemented with 10% FBS in the presence (C) or absence (D) of tetracycline, as shown in the legend. E) Growth curves of T. brucei BSF in low-fat media. The graphs represent the growth curves over 48 h of T. brucei BSF WT control and T. brucei BSF Tb-Δ6 knock-down (Δ6-KD), and Tb-Δ6 overexpression (Δ6-OE) cells, when they are cultured in HMI-11 supplemented with 5% FBS in the absence of tetracycline. F) Growth curves of T. brucei PCF in low-fat media. The graphs represent the growth curves over 72 h of T. brucei PCF WT control and T. brucei PCF Tb-Δ6 knock-down (Δ6-KD), Tb-Δ6 overexpression (Δ6-OE) and Tb-Δ6 add-back (Δ6-OK) cells, when they are cultured in SDM-79 supplemented with 1.25% FBS in the absence of tetracycline. For all growth curves values are the mean of three independent biological replicates (n = 3). Error bars represent the standard deviation of each mean. Statistical analysis was performed by GraphPad PRISM 6.0 using One-way ANOVA multiple comparisons based on a Tukey t-test with a 95% confidence interval. (DOCX) [file ppat.1012691.s021.docx]

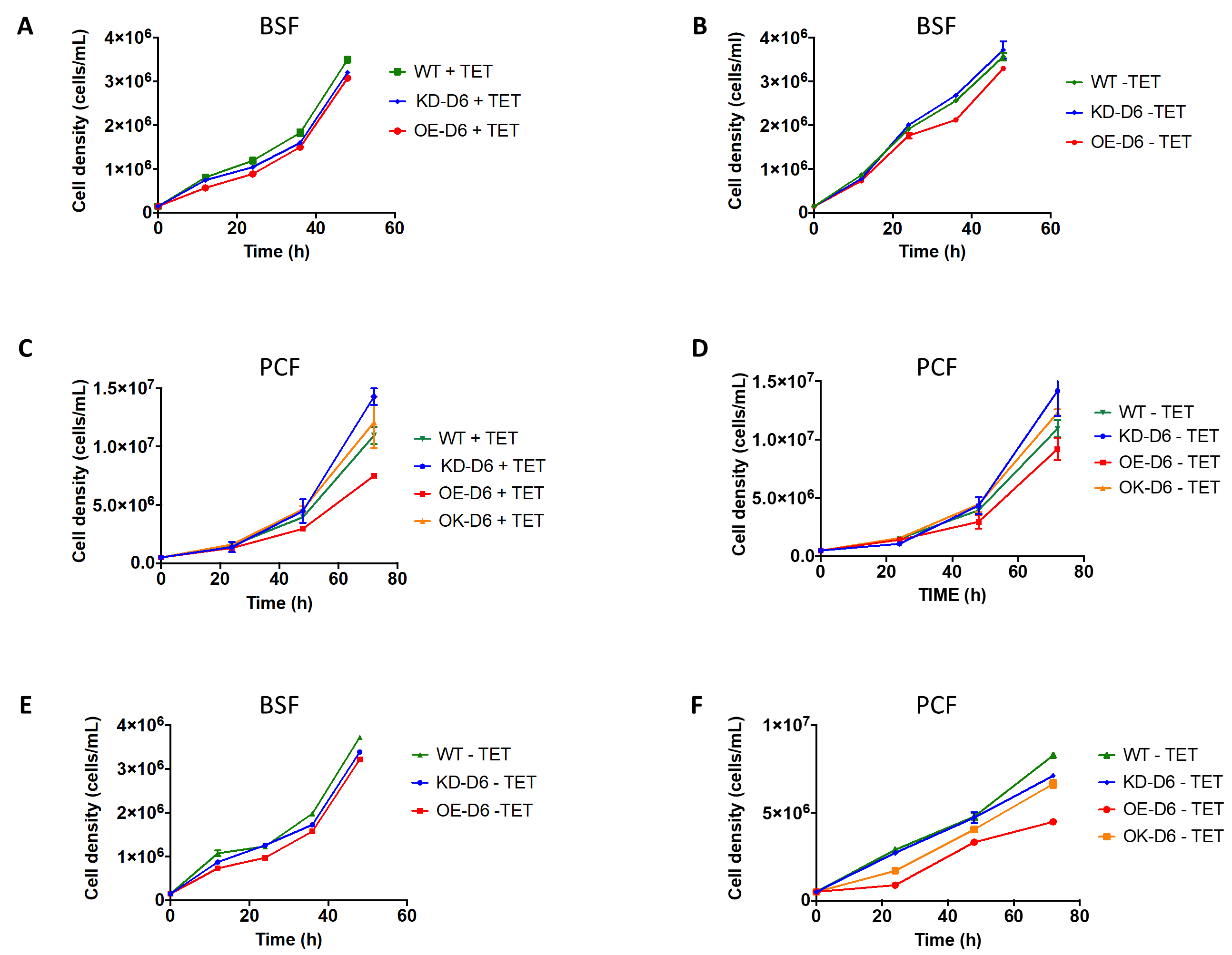


S11 Fig. A, B) Growth curves of *T. brucei* BSF in high-fat media. The graphs represent the growth curves over 48 h of *T. brucei* BSF WT control and *T. brucei* BSF Tb-Δ6 knock-down (Δ6-KD), and Tb-Δ6 overexpression (Δ6-OE) cells, when they are cultured in HMI-11 supplemented with 10% FBS in the presence (A) or absence (B) of tetracycline as shown in the legend. C, D) Growth curves of *T. brucei* PCF in high-fat media. The graphs represent the growth curves over 72 h of *T. brucei* PCF WT control and *T. brucei* PCF Tb-Δ6 knock-down (Δ6-KD), Tb-Δ6 overexpression (Δ6-OE) and Tb-Δ6 add-back (Δ6-OK) cells, when they are cultured in SDM-79 supplemented with 10% FBS in the presence (C) or absence (D) of tetracycline, as shown in the legend. E) Growth curves of *T. brucei* BSF in low-fat media. The graphs represent the growth curves over 48 h of *T. brucei* BSF WT control and *T. brucei* BSF Tb-Δ6 knock-down (Δ6-KD), and Tb-Δ6 overexpression (Δ6-OE) cells, when they are cultured in HMI-11 supplemented with 5% FBS in the absence of tetracycline. F) Growth curves of *T. brucei* PCF in low-fat media. The graphs represent the growth curves over 72 h of *T. brucei* PCF WT control and *T. brucei* PCF Tb-Δ6 knock-down (Δ6-KD), Tb-Δ6 overexpression (Δ6-OE) and Tb-Δ6 add-back (Δ6-OK) cells, when they are cultured in SDM-79 supplemented with 1.25% FBS in the absence of tetracycline. For all growth curves values are the mean of three independent biological replicates (n=3). Error bars represent the standard deviation of each mean. Statistical analysis was performed by GraphPad PRISM 6.0 using One-way ANOVA multiple comparisons based on a Tukey t-test with a 95% confidence interval.
