# Supplementary material for: Exploring the activity of the putative Δ6-desaturase and its role in bloodstream form life-cycle transitions in Trypanosoma brucei
Source: PLoS Pathog. 2025 Feb 18;21(2):e1012691. doi: 10.1371/journal.ppat.1012691 (PMC11867338; doi:10.1371/journal.ppat.1012691)
Supplement: S12 Fig — The graphs represent the growth curves over 48 h of T. brucei PCF (A) and BSF (B) WT control and T. brucei Δ6-desaturase knock down (KD-D6), when they are cultured in SDM-79 supplemented with 1.25% FBS (A) and HMI-11 supplemented with 5% FBS (B) both added with 10 µM DHA (22:6) (dotted/dashed lines), in the absence of tetracycline as shown in the legend. Values are the mean of three independent biological replicates (n = 3). Error bars represent the standard deviation of each mean (±). All FAs were identified using GC-MS based upon retention time, fragmentation, and comparison with standards. Statistical analysis was performed by GraphPad PRISM 6.0 using One-way ANOVA multiple comparisons based on a Tukey t-test with a 95% confidence interval. Note: the solid lines represent data taken from S11E and S11F Fig used here for a more complete comparison. (DOCX) [file ppat.1012691.s022.docx]

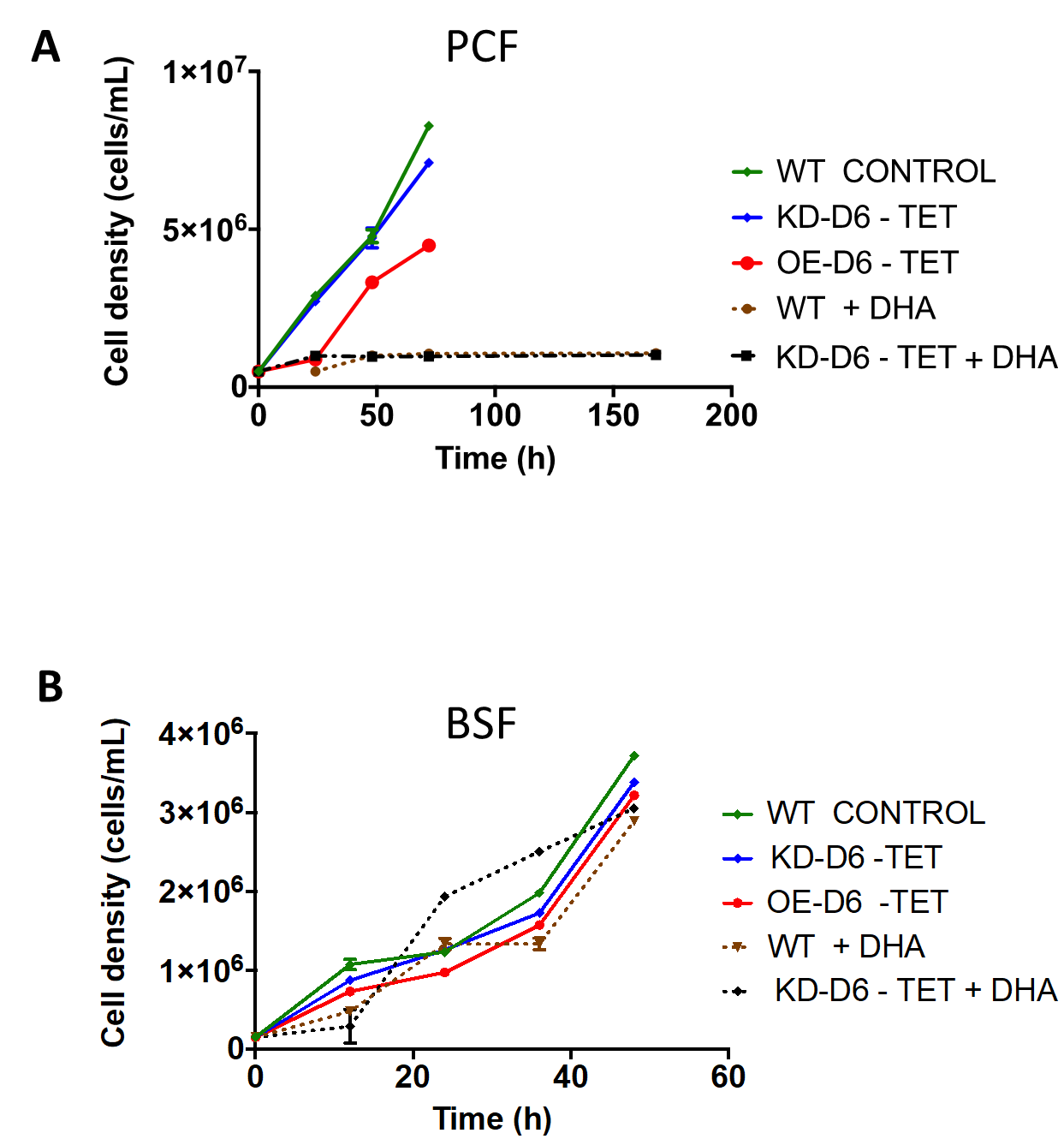


S12 Fig. Growth curves of Tb-Δ6 genetically modified *T. brucei* PCF and BSF supplemented with DHA in the absence of tetracycline. The graphs represent the growth curves over 48 h of *T. brucei* PCF (A) and BSF (B) WT control and *T. brucei* Δ6-desaturase knock down (KD-D6), when they are cultured in SDM-79 supplemented with 1.25% FBS (A) and HMI-11 supplemented with 5% FBS (B) both added with 10 µM DHA (22:6) (dotted/dashed lines), in the absence of tetracycline as shown in the legend. Values are the mean of three independent biological replicates (n=3). Error bars represent the standard deviation of each mean (±). All FAs were identified using GC-MS based upon retention time, fragmentation, and comparison with standards. Statistical analysis was performed by GraphPad PRISM 6.0 using One-way ANOVA multiple comparisons based on a Tukey t-test with a 95% confidence interval. Note: the solid lines represent data taken from Figure S11E and S11F used here for a more complete comparison.
