# Supplementary material for: Exploring the activity of the putative Δ6-desaturase and its role in bloodstream form life-cycle transitions in Trypanosoma brucei
Source: PLoS Pathog. 2025 Feb 18;21(2):e1012691. doi: 10.1371/journal.ppat.1012691 (PMC11867338; doi:10.1371/journal.ppat.1012691)
Supplement: S15 Fig — A) The bar chart shows the different 18C FAs (X axis, the order follows increasing retention time) and the relative abundance (Y axis) found in T. brucei BSF (A, B and C) and PCF (D, E and F) WT controls, when the cells are cultured for 48 h in HMI-11 with 5% FBS (A, B and C) or SDM-79 with 1.25% FBS (D, E and F) supplemented with 10 µM DHA, as shown in the legend, and compared to T. brucei BSF and PCF WT in HMI-11 or SDM-79 with 10% FBS. Values are the mean of three independent biological replicates (n = 3). Error bars represent the standard deviation of each mean (±). All FAs were identified using GC-MS based upon retention time, fragmentation, and comparison with standards. Statistical analysis was performed by GraphPad PRISM 6.0 using One-way ANOVA multiple comparisons based on a Tukey t-test with a 95% confidence interval, where **** is p ≤ 0.0001, ** is p ≤ 0.01 and * is p ≤ 0.05. Note: ‘ = first eluted isomer; “ = second eluted isomer. (S2 and S3 Appendices). (DOCX) [file ppat.1012691.s025.docx]

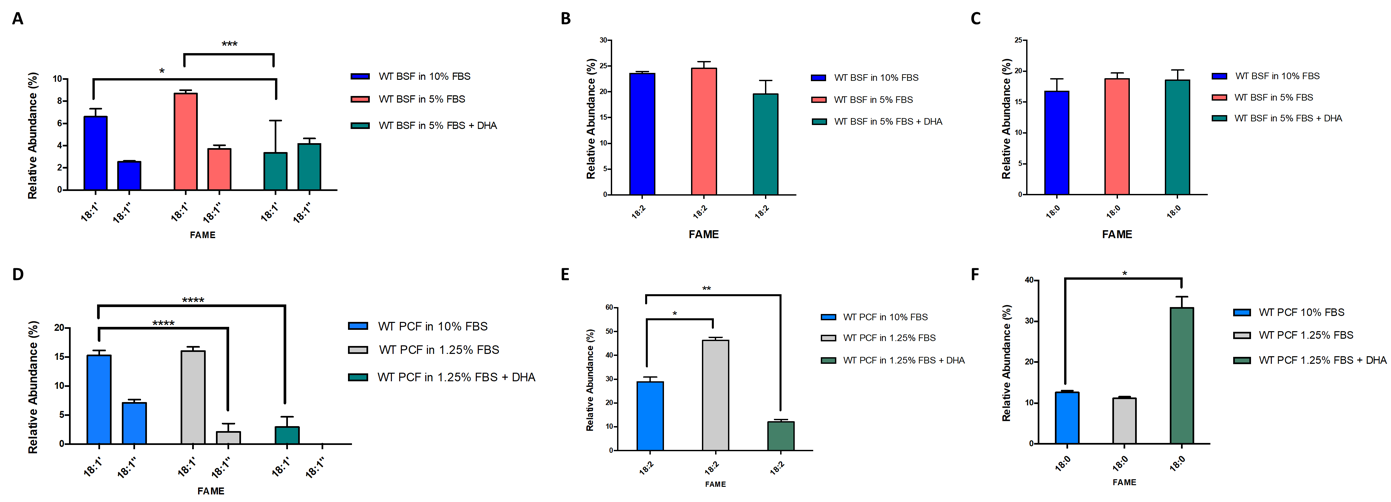


S15 Fig. GC-MS analysis of the 18C fatty acids in *T. brucei* BSF and PCF WT in low-fat media supplemented with DHA. A) The bar chart shows the different 18C FAs (X axis, the order follows increasing retention time) and the relative abundance (Y axis) found in *T. brucei* BSF (A, B and C) and PCF (D, E and F) WT controls, when the cells are cultured for 48 h in HMI-11 with 5% FBS (A, B and C) or SDM-79 with 1.25% FBS (D, E and F) supplemented with 10 µM DHA, as shown in the legend, and compared to *T. brucei* BSF and PCF WT in HMI-11 or SDM-79 with 10% FBS. Values are the mean of three independent biological replicates (n=3). Error bars represent the standard deviation of each mean (±). All FAs were identified using GC-MS based upon retention time, fragmentation, and comparison with standards. Statistical analysis was performed by GraphPad PRISM 6.0 using One-way ANOVA multiple comparisons based on a Tukey t-test with a 95% confidence interval, where **** is p ≤ 0.0001, ** is p ≤ 0.01 and * is p ≤ 0.05. Note: ‘ = first eluted isomer; “ = second eluted isomer. (Appendix B and C)
