# Supplementary material for: Exploring the activity of the putative Δ6-desaturase and its role in bloodstream form life-cycle transitions in Trypanosoma brucei
Source: PLoS Pathog. 2025 Feb 18;21(2):e1012691. doi: 10.1371/journal.ppat.1012691 (PMC11867338; doi:10.1371/journal.ppat.1012691)
Supplement: S16 Fig — The spectra show PI-containing lipids obtained by scanning for parent ion of 241 m/z for Δ6-KD BSF (A) grown for 48 h in HMI-11 with 5% FBS in the presence of tetracycline, and WT control BSF (B), Δ6-KD BSF (C) and Δ6-OE BSF (D) grown in HMI-11 with 10% FBS. The species of interest are labelled (PIs black) as reported in the text and highlighted by arrows (PIs black). Spectra are representative of experiments conducted in three independent biological replicates (n = 3). E) ESI-MS/MS quantification of PIs in the knock-down of Tb-Δ6 in T. brucei PCF in low-fat media. The bar charts show the difference in PI and IPC species (X axis) and the normalised intensity (Y axis, cps) found in T. brucei PCF KD-D6 and WT control, when the cells are cultured for 48 h in HMI-11 supplemented with 1.25% FBS, in the presence of tetracycline as shown in the legend. The relative intensities of PIs were normalised against the intensity of PI (15:0/18:1(d7)) at 847.13 m/z contained in SPLASH internal standard. Values are the mean of three independent biological replicates (n = 3). Standard deviation of each mean (±) is calculated for the normalised intensities. Statistical analysis was performed by GraphPad PRISM 6.0 using One-way ANOVA multiple comparisons based on a Tukey t-test with a 95% confidence interval, where ** is p ≤ 0.01 and * is p ≤ 0.05. (S4 Appendix) E) Inositol-phosphoryl ceramide (IPC) and sphingomyelin (SM) synthetic pathway in T. brucei. Schematic representation of the synthesis of IPC from ceramide and PI by inositol-phosphoryl ceramide synthase (SLS1) and of SM from ceramide and PC by sphingomyelin synthase (SLS4), through release of diacylglycerol (DAG). R1 and R2 represent the fatty chain. (DOCX) [file ppat.1012691.s026.docx]

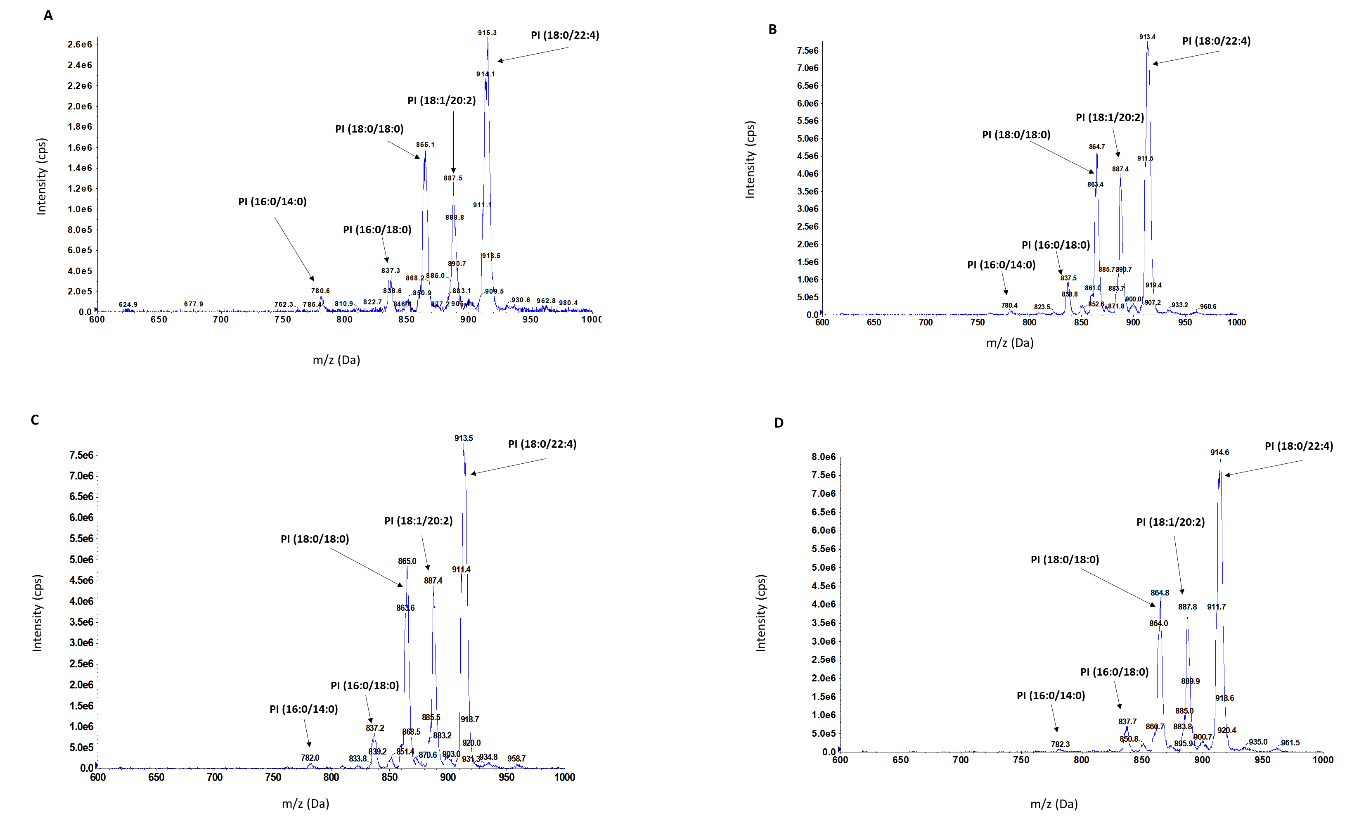


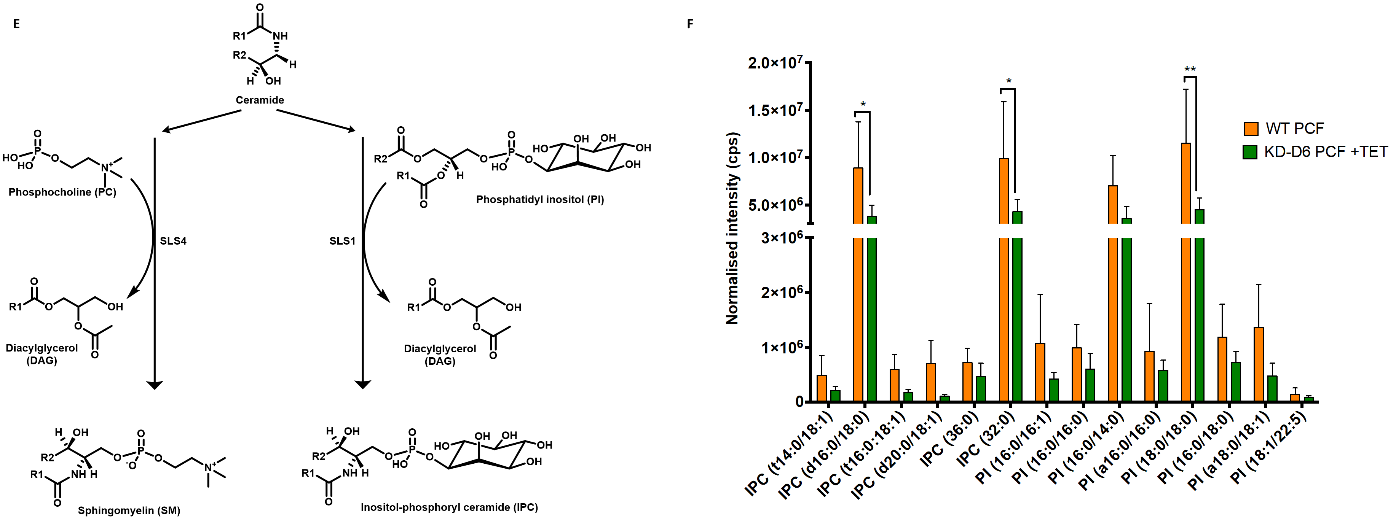


S16 Fig. A-D) ESI-MS/MS spectra of PI-containing lipids for Tb-Δ6 genetically manipulated *T. brucei* BSF grown in high- and low-fat media. The spectra show PI-containing lipids obtained by scanning for parent ion of 241 m/z for Δ6-KD BSF (A) grown for 48 h in HMI-11 with 5% FBS in the presence of tetracycline, and WT control BSF (B), Δ6-KD BSF (C) and Δ6-OE BSF (D) grown in HMI-11 with 10% FBS. The species of interest are labelled (PIs black) as reported in the text and highlighted by arrows (PIs black). Spectra are representative of experiments conducted in three independent biological replicates (n = 3). E) Inositol-phosphoryl ceramide (IPC) and sphingomyelin (SM) synthetic pathway in *T. brucei*. Schematic representation of the synthesis of IPC from ceramide and PI by inositol-phosphoryl ceramide synthase (SLS1) and of SM from ceramide and PC by sphingomyelin synthase (SLS4), through release of diacylglycerol (DAG). R1 and R2 represent the fatty acid chains. F) ESI-MS/MS quantification of PIs in the knock-down of Tb-Δ6 in *T. brucei* PCF in low-fat media. The bar charts show the difference in PI and IPC species (X axis) and the normalised intensity (Y axis, cps) found in *T. brucei* PCF KD-D6 and WT control, when the cells are cultured for 48 h in HMI-11 supplemented with 1.25% FBS, in the presence of tetracycline as shown in the legend. The relative intensities of PIs were normalised against the intensity of PI (15:0/18:1(d7)) at 847.13 m/z contained in SPLASH internal standard. Values are the mean of three independent biological replicates (n=3). Standard deviation of each mean (±) is calculated for the normalised intensities. Statistical analysis was performed by GraphPad PRISM 6.0 using One-way ANOVA multiple comparisons based on a Tukey t-test with a 95% confidence interval, where ** is p ≤ 0.01 and * is p ≤ 0.05. (Appendix D)
