# Supplementary material for: Exploring the activity of the putative Δ6-desaturase and its role in bloodstream form life-cycle transitions in Trypanosoma brucei
Source: PLoS Pathog. 2025 Feb 18;21(2):e1012691. doi: 10.1371/journal.ppat.1012691 (PMC11867338; doi:10.1371/journal.ppat.1012691)
Supplement: S17 Fig — Daughter ion scans from Δ6-OE BSF (grown in low fat media) in negative mode for PI and IPC at m/z 780. B) Fragmentation pattern of IPC (t16:0/18:1). Daughter ion scans from Δ6-OE BSF (grown in low fat media) in negative mode for IPC at m/z 794. C) Fragmentation patterns of PI (16:0/18:0) and IPC (d20:0/18:1). Daughter ion scans from Δ6-OE BSF (grown in low fat media) in negative mode for PI and IPC at m/z 835/837. D) Fragmentation pattern of SM (d16:0/18:0). Daughter ion scans from Δ6-OE BSF (grown in low fat media) in positive mode for SM at m/z 706. The structures have coloured portions to highghlight the correspondent fragments in the spectra indicated with circles of the same colour. (DOCX) [file ppat.1012691.s027.docx]

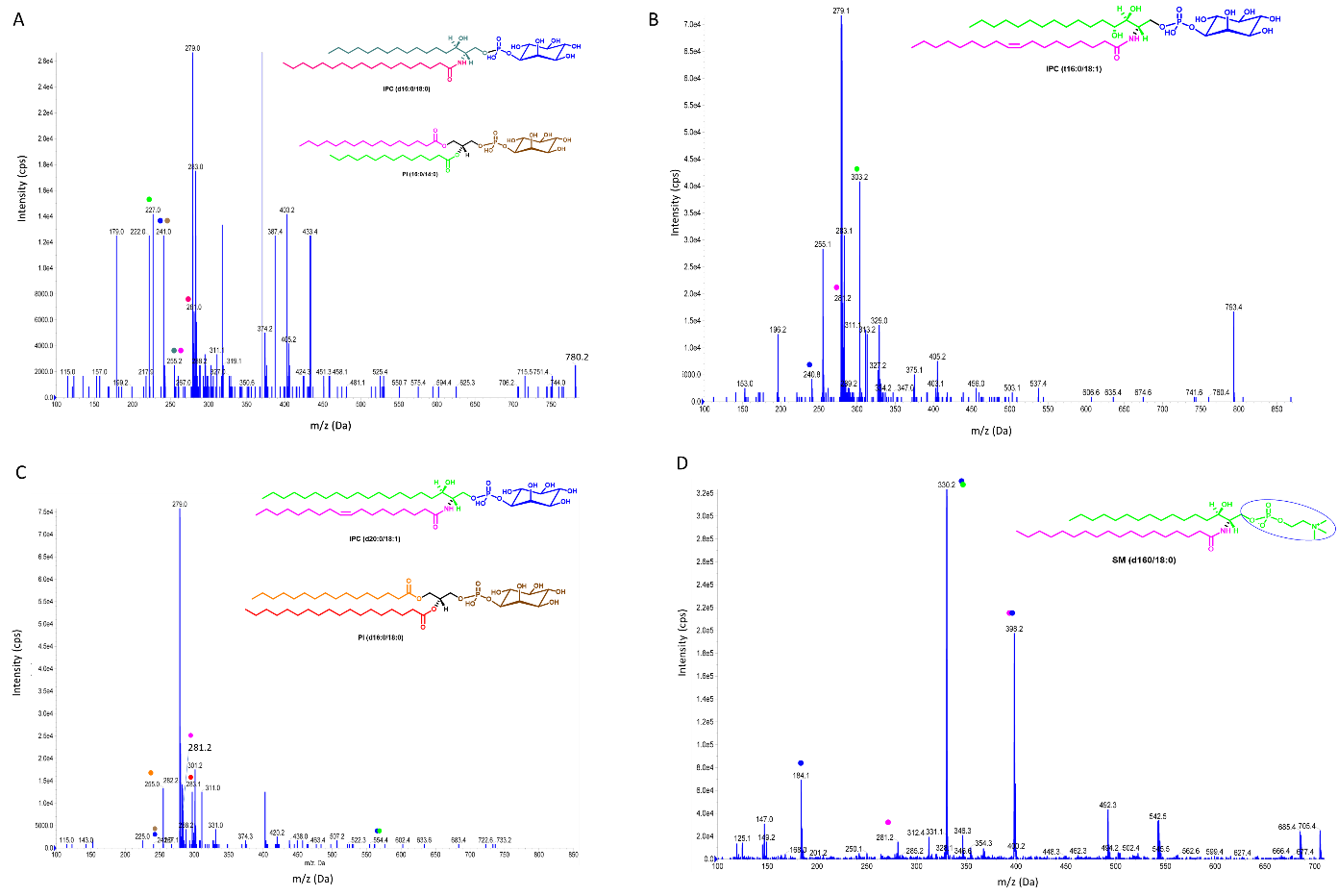


S17 Fig. A) Fragmentation pattern of PI (16:0/14:0) and IPC (d16:0/18:0). Daughter ion scans from Δ6-OE BSF (grown in low fat media) in negative mode for PI and IPC at m/z 780. B) Fragmentation pattern of IPC (t16:0/18:1). Daughter ion scans from Δ6-OE BSF (grown in low fat media) in negative mode for IPC at m/z 794. C) Fragmentation patterns of PI (16:0/18:0) and IPC (d20:0/18:1). Daughter ion scans from Δ6-OE BSF (grown in low fat media) in negative mode for PI and IPC at m/z 835/837. D) Fragmentation pattern of SM (d16:0/18:0). Daughter ion scans from Δ6-OE BSF (grown in low fat media) in positive mode for SM at m/z 706. The structures have coloured portions to highghlight the correspondent fragments in the spectra indicated with circles of the same colour.
