# Supplementary material for: Exploring the activity of the putative Δ6-desaturase and its role in bloodstream form life-cycle transitions in Trypanosoma brucei
Source: PLoS Pathog. 2025 Feb 18;21(2):e1012691. doi: 10.1371/journal.ppat.1012691 (PMC11867338; doi:10.1371/journal.ppat.1012691)
Supplement: S18 Fig — The spectra show choline-phosphate-containing lipids obtained by scanning for parent ion of 184 m/z for WT control BSF (A) and Δ6-KD BSF (B) and Δ6-OE BSF (C) grown for 48 h in HMI-11 with 5% FBS, in the presence of Tet. The SM of interest is labelled as reported in the text and highlighted by an arrow (red). The major SM and PC species are also annotated. a, alkylacyl. Spectra are representative of experiments conducted in three independent biological replicates (n = 3). D) ESI-MS/MS quantification of sphingomyelin in Tb-Δ6 genetically manipulated T. brucei BSF in low-fat media. The bar chart shows the difference in SM species at 706 m/z (X axis) and the normalised intensity (Y axis, cps) found in Tb-Δ6 genetically modified T. brucei BSF (OE-D6) and WT control, when the cells are cultured for 48 h in HMI-11 supplemented with 5% FBS, in the presence of tetracycline and treated or not with EC10 of clemastine fumarate, as shown in the legend. The relative intensities of IPCs were normalised against the intensity of PI (15:0/18:1(d7)) at 847.13 m/z contained in SPLASH internal standard. Values are the mean of three independent biological replicates (n = 3). Standard deviation of each mean (±) is calculated for the normalised intensities. Statistical analysis was performed by GraphPad PRISM 6.0 using One-way ANOVA multiple comparisons based on a Tukey t-test with a 95% confidence interval. (S4 Appendix). (DOCX) [file ppat.1012691.s028.docx]

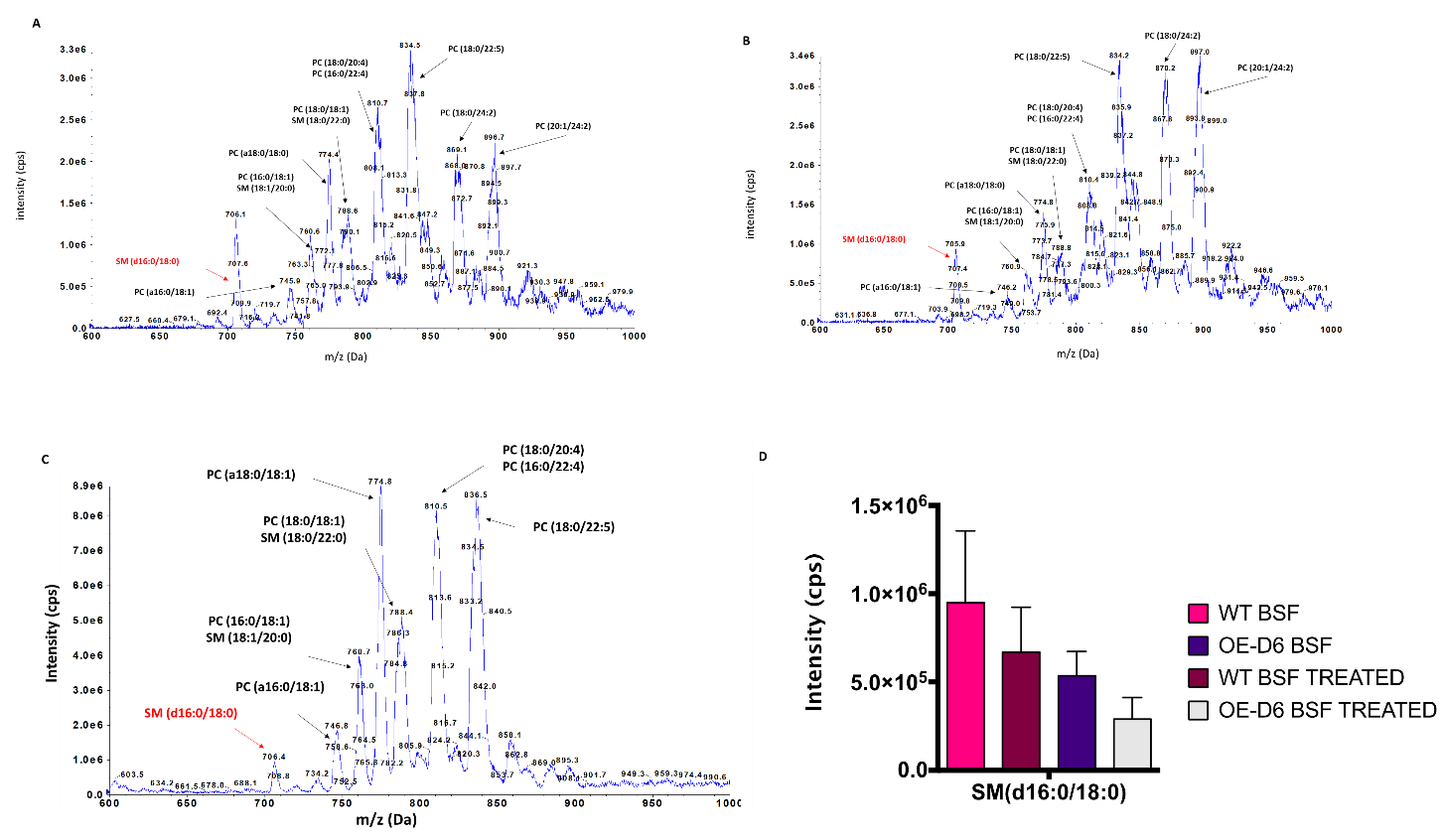


S18 Fig. A-C) ESI-MS/MS spectra of PC-containing lipids for Tb-Δ6 genetically manipulated *T. brucei* BSF grown in low-fat media. The spectra show choline-phosphate-containing lipids obtained by scanning for parent ion of 184 m/z for WT control BSF (A) and Δ6-KD BSF (B) and Δ6-OE BSF (C) grown for 48 h in HMI-11 with 5% FBS, in the presence of Tet. The SM of interest is labelled as reported in the text and highlighted by an arrow (red). The major SM and PC species are also annotated. a, alkylacyl. Spectra are representative of experiments conducted in three independent biological replicates (n = 3). D) ESI-MS/MS quantification of sphingomyelin in Tb-Δ6 genetically manipulated *T. brucei* BSF in low-fat media. The bar chart shows the difference in SM species at 706 m/z (X axis) and the normalised intensity (Y axis, cps) found in Tb-Δ6 genetically modified *T. brucei* BSF (OE-D6) and WT control, when the cells are cultured for 48 h in HMI-11 supplemented with 5% FBS, in the presence of tetracycline and treated or not with EC_10_ of clemastine fumarate, as shown in the legend. The relative intensities of IPCs were normalised against the intensity of PI (15:0/18:1(d7)) at 847.13 m/z contained in SPLASH internal standard. Values are the mean of three independent biological replicates (n=3). Standard deviation of each mean (±) is calculated for the normalised intensities. Statistical analysis was performed by GraphPad PRISM 6.0 using One-way ANOVA multiple comparisons based on a Tukey t-test with a 95% confidence interval. (Appendix D)
