# Supplementary material for: Exploring the activity of the putative Δ6-desaturase and its role in bloodstream form life-cycle transitions in Trypanosoma brucei
Source: PLoS Pathog. 2025 Feb 18;21(2):e1012691. doi: 10.1371/journal.ppat.1012691 (PMC11867338; doi:10.1371/journal.ppat.1012691)
Supplement: S19 Fig — Dose-response curve and correspondent table of EC50 values obtained for clemastine fumarate against WT T. brucei BSF (A), Δ6-OE T. brucei BSF induced with Tet for 48 h (B) and Δ6-OE T. brucei BSF non-induced with Tet (C) grown in HMI-11 with 5% of FBS. Data analysis was carried using GraFit 5.0 (Erithacus Software). (DOCX) [file ppat.1012691.s029.docx]

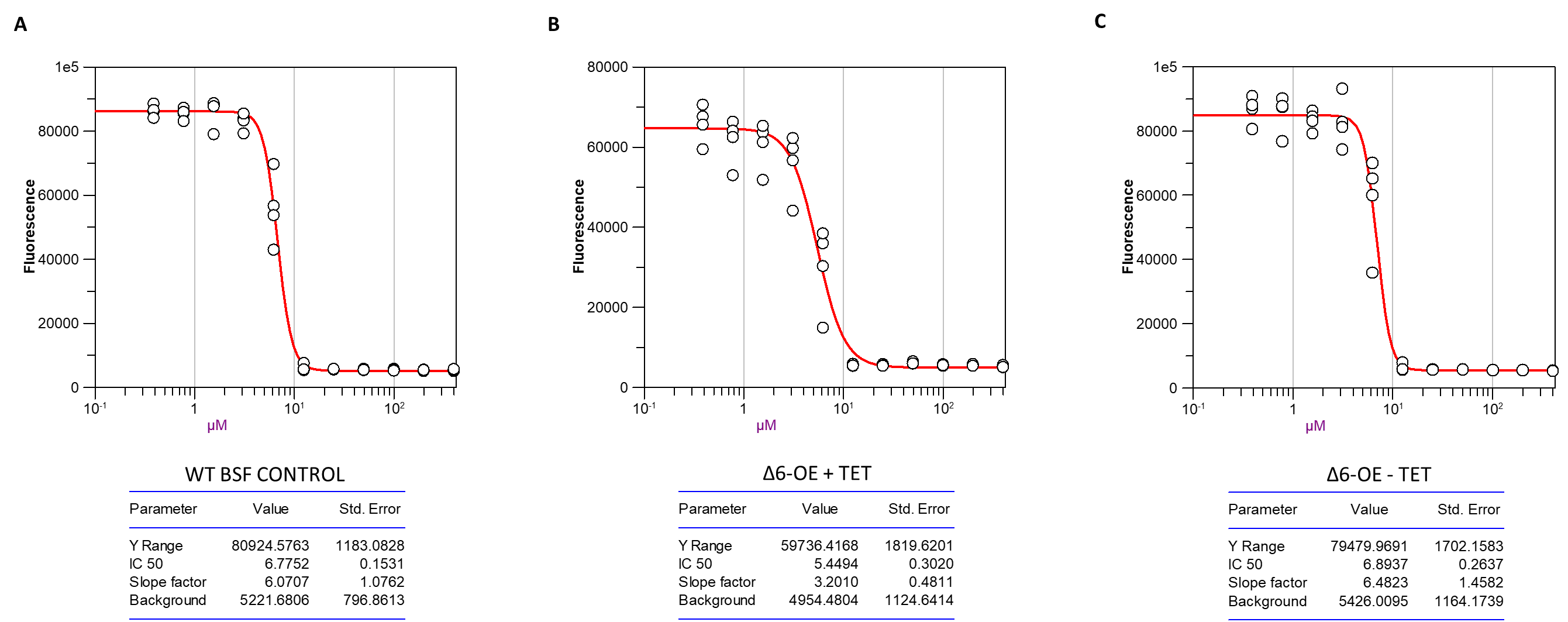


S19 Fig. Dose-response assay for clemastine fumarate against Tb-Δ6 genetically manipulated *T. brucei* BSF grown in low-fat media. Dose-response curve and correspondent table of EC_50_ values obtained for clemastine fumarate against WT *T. brucei* BSF (A), Δ6-OE *T. brucei* BSF induced with Tet for 48 h (B) and Δ6-OE *T. brucei* BSF non-induced with Tet (C) grown in HMI-11 with 5% of FBS. Data analysis was carried using GraFit 5.0 (Erithacus Software).
