# Supplementary material for: Exploring the activity of the putative Δ6-desaturase and its role in bloodstream form life-cycle transitions in Trypanosoma brucei
Source: PLoS Pathog. 2025 Feb 18;21(2):e1012691. doi: 10.1371/journal.ppat.1012691 (PMC11867338; doi:10.1371/journal.ppat.1012691)
Supplement: S20 Fig — Red highlights represent the identified peptides. (DOCX) [file ppat.1012691.s030.docx]

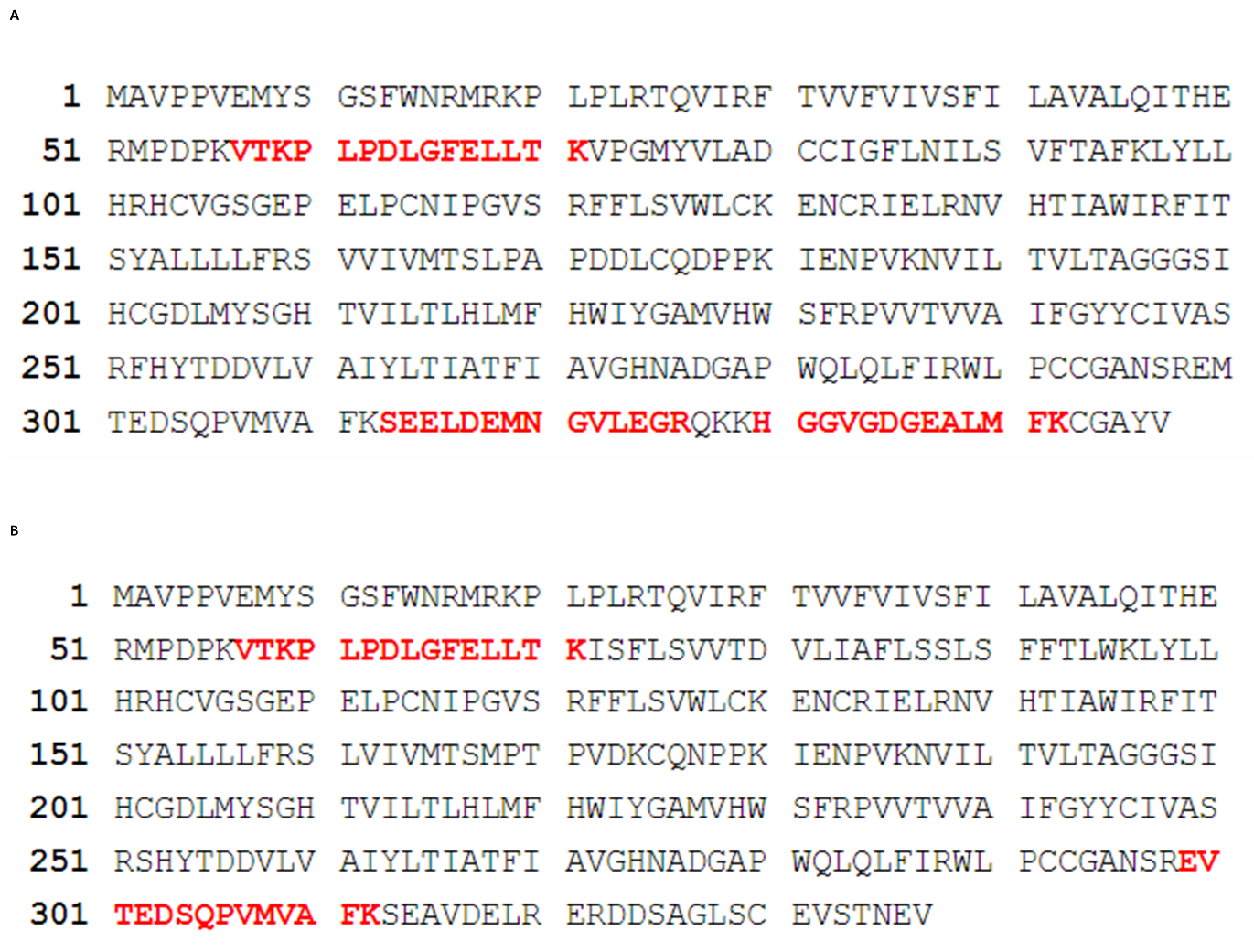


S20 Fig. SLS1 and SLS4 sequences identification via SWATH MS. SLS4 (A) and SLS1 (B) protein sequence are reported as presented in the proteomics analytical software MASCOT. Red highlights represent the identified peptides.
